# Accurate programmed multifunctional nano-missiles for self-promoted deep delivery and synergistic cascade tumor therapy: Tactfully collaborating chemosynthesis with tumor microenvironment remodeling

**DOI:** 10.7150/thno.74550

**Published:** 2022-07-04

**Authors:** Runxin Lu, Siqi Wang, Zhongzhen Yang, Lin Zhou, Chunyan Yang, Yong Wu

**Affiliations:** 1 Department of Pharmacy, Evidence-Based Pharmacy Center, Key Laboratory of Birth Defects and Related Diseases of Women and Children (Sichuan University), West China Second University Hospital, Sichuan University, Chengdu, China.; 2 Key Laboratory of Drug-Targeting and Drug Delivery System of the Education Ministry and Sichuan Province, Sichuan Engineering Laboratory for Plant-Sourced Drug and Sichuan Research Center for Drug Precision Industrial Technology, West China School of Pharmacy, Sichuan University, Chengdu, China.

**Keywords:** programmed nanodrug, chemosynthesis, tumor microenvironment remodeling, self-promoted drug delivery, multimodal synergistic therapy

## Abstract

**Rationale:** Triple-negative breast cancer (TNBC) is considered one of the highest-risk subtypes of breast cancer and has dismal prognosis. The management of aggressive TNBC remains a formidable challenge. Tumor microenvironment (TME), with the unique features, which can serve as the “soil” for the growth and survival of tumor cells (the “seeds”), plays an important regulatory role in the occurrence, proliferation and metastasis of tumors. Catalytic tumor therapy, which can destroy the homeostasis of TME, affect the occurrence and progress of tumors in an all-round way and further magnify chemotherapy, is a quite potential tactic for TNBC-treatment.

**Methods:** Herein, accurate programmed multifunctional cascade nano-missiles (GOx+L-Arg-NM/PTX-NM) composed of novel intelligent all-in-one “nano-rocket” (the drug delivery system) and “ammunitions” (the therapeutic agents) are innovatively constructed by mimicking the functionalities of military precision-guided missiles. Ammunitions can be precisely and effectively transported to the core region of TNBC (the “battlefield”) by organic modification on the surface of nano-rocket *via* chemical means. Once successfully internalized by TNBC cells, the nano-missiles can automatically trigger relevant cascade reactions without external stimulation, prominently disrupt the homeostasis of TME, and produce a “bomb-like” attack on tumors, further promoting the chemotherapy.

**Results:** Both *in vitro* and *in vivo* investigations indicated that the innovative nano-missiles could deliver ammunitions to the core area of TNBC to the utmost extent, dramatically ablate tumor and restrain tumor metastasis* via* orchestrated multimodal synergistic starvation/oxidation/gas/chemotherapy.

**Conclusion:** The well-designed multifunctional nano-missiles may emerge as a new paradigm to suppress the malignant proliferation and metastasis of TNBC, offering a promising approach for the next generation cancer therapy.

## Introduction

Breast cancer has surpassed lung cancer as the most commonly diagnosed cancer based on the GLOBOCAN estimates produced by the International Agency for Research on Cancer in 2020 1. Triple-negative breast cancer (TNBC) is considered heterogeneous and the most aggressive breast cancer subtype, particularly prone to metastasis 2, 3. Tragically, conventional chemotherapy is the only established therapeutic option for TNBC patients to improve disease outcomes ascribed to the lack of definitive targets. Patients with early stages of TNBC are cured with chemotherapy, whereas the median OS (overall survival) is only 13-18 months when occur tumor metastasis 4. In consequence, new treatment options which can effectively restrain TNBC proliferation and metastasis simultaneously are urgently needed.

Tumor microenvironment (TME), with the unique features, which can serve as the “soil” for the growth and survival of tumor cells (the “seeds”), plays an important regulatory role in the occurrence, proliferation and metastasis of tumors 5-7. Indeed, conventional chemotherapy has shown limited success against cancers due to the complexity and heterogeneity of TME. And drug delivery in tumors is restricted by several biological barriers in TME 7, 8. Destroying the homeostasis of TME, such as glucose consumption or redox unequilibrium, rather than directly killing tumor cells, is a promising strategy which can improve the therapeutic benefits in cancer treatment and affect the occurrence and progress of tumors in an all-round way 9. Catalytic tumor therapy, based on enzymatic reactions in the TME, has recently aroused great interest 10. Glucose oxidase (GOx), as an endogenous biocatalyst, can effectively catalyze the oxidization of intratumoral glucose into gluconic acid and toxic hydrogen peroxide (H_2_O_2_) 11, 12, having a great application prospect in the field of synergistic cancer therapy 13-17. Our previous study has demonstrated that GOx can efficiently destroy the homeostasis of TME, amplify chemotherapy and achieve enhanced tumor ablation 18.

Nitric oxide (NO), a “star” gasotransmitter, plays a prominent role in regulating physiological/pathophysiological activities in terms of vasodilation, angiogenesis, vascular permeability and chemosensitizing effect 19. Sufficient NO can cause DNA/mitochondria damage, apoptotic enhancement, metastatic suppression, hypoxic remittence, and so on 20. Furthermore, NO also shows great potential in modulating the TME to enhance EPR effect, elevate drug delivery into tumor and consequently strengthen the curative effect of chemotherapy 21. However, due to its poor stability, short half-life (< 5 s) and short diffusion distances (20-160 μm), NO is difficult to be directly utilized to kill tumor cells, greatly restricting its application 19, 22. Ulteriorly, NO delivery technologies remain severely limited in clinical application attributed to the existence of toxic groups, spontaneously and excessively rapid release of NO, limited loading, and imprecise tumor targeting, though a series of NO donor drugs 23, 24 or NO delivery vectors 25, 26 have been developed. Therefore, setting up a smart NO release platform which can be triggered *in situ* and generate abundant NO in the tumor area is eagerly anticipated. Enhanced tumor-specific delivery is a prerequisite for drugs to successfully achieve superb tumor therapy.

**Figure 1:**
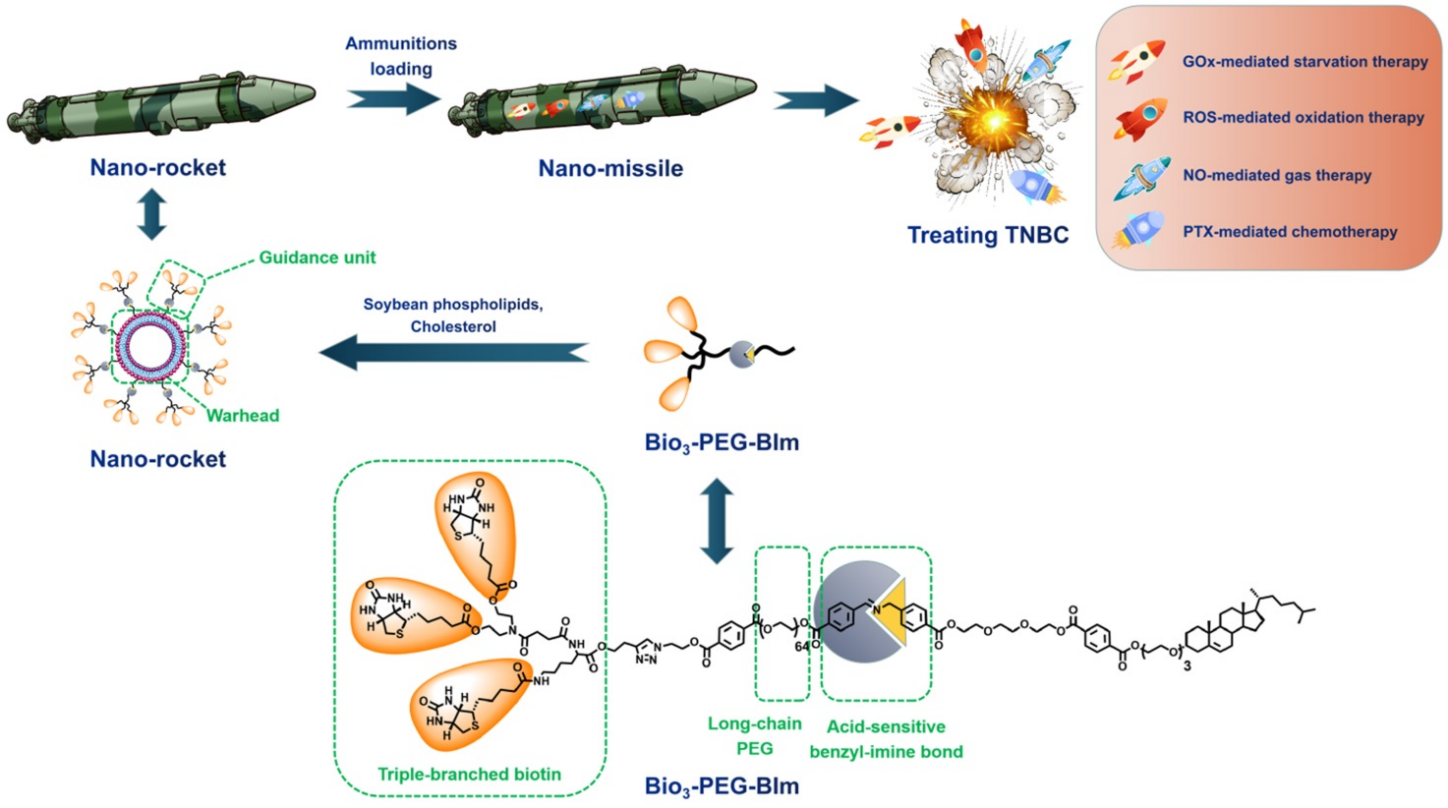
The design concept of novel accurate programmed TNBC-targeting nano-missile.

Herein, we constructed a novel accurate programmed TNBC-targeting “nano-missile (NM)” by mimicking the functionalities of military precision-guided missiles, to accomplish accurate and satisfactory antagonism of TNBC through a multidimensional therapy that can simultaneously suppress tumor proliferation and metastasis. This newly developed nano-missile mainly consists of two parts: the launch vehicle (“nano-rocket”) and the therapeutic agent (“ammunitions”) (Figure 1), wherein the all-in-one nano-rocket is capable of delivering the ammunitions to the core region of TNBC (the “battlefield”) with maximum accuracy, thus generating powerful “bomb-like” attacks.

Guidance unit, as the most important component of the newly developed nano-rocket, is designed by integrating the functions of triple-branched biotin fragment, long-chain polyethylene glycol (PEG) and acid-sensitive benzyl imine bond (Figure 1). Biotin, a water-soluble vitamin, can specifically bind to the sodium-dependent multivitamin transporter (SMVT), which has been shown to be over-expressed in a variety of malignant cancers cells (such as 4T1 and MDA-MB-231) 27. Previously, our group proved for the first time that increasing the density of biotin on the surface of nanocarriers can significantly enhance the breast cancer targeting ability, and the branching structure of biotin fragments also has an important influence on the affinity to SMVT 28. To be more specific, compared with other branch types, such as single, double and tetra, the triple-branched biotin fragment used in the guidance unit has the optimum TNBC targeting ability, thereby granting the nano-rocket excellent “strike precision”. Long chain PEG can endow the nano-rocket strong “stealth” and “long-range combat” capabilities 29-31. However, there is a contradictory balance between long circulation and cellular internalization restriction when PEGylation 32, 33. An acid-sensitive bond participation can overcome this dilemma, enabling PEG chains to play a role in systemic long circulation and stealth, trigger the shedding when the nano-rocket reached TME (pH 6.5). Benzyl imine bond stand out and is selected as the acid-responsive part of the guidance unit after systematic screening based on the following considerations: (i) stable enough under physiological conditions (pH 7.4) and more sensitive to the TME (pH 6.5) than other traditional acid-sensitive bonds, such as hydrazone bond 34; (ii) the amino group exposed from the hydrolysis of the benzyl imine bond can be protonized in the TME to realize charge reversal, further promoting the deep penetration of the nano-rocket in the TNBC. Skillfully using chemical means, a new smart ligand (Bio_3_-PEG-BIm) of nano-rocket was successfully designed and synthesized for the first time (Figure 1). In this way, the above-mentioned multiple functions can be reflected on the Bio_3_-PEG-BIm concurrently, successfully maximizing the function of a single ligand, which also makes the preparation of the nano-missile easier. The well-devised intelligent nano-rocket enables the precise programmed delivery of ammunitions into TNBC, and hopefully only triggers the therapeutic process within the tumorous tissues with negligible damages to normal tissues.

**Figure 2:**
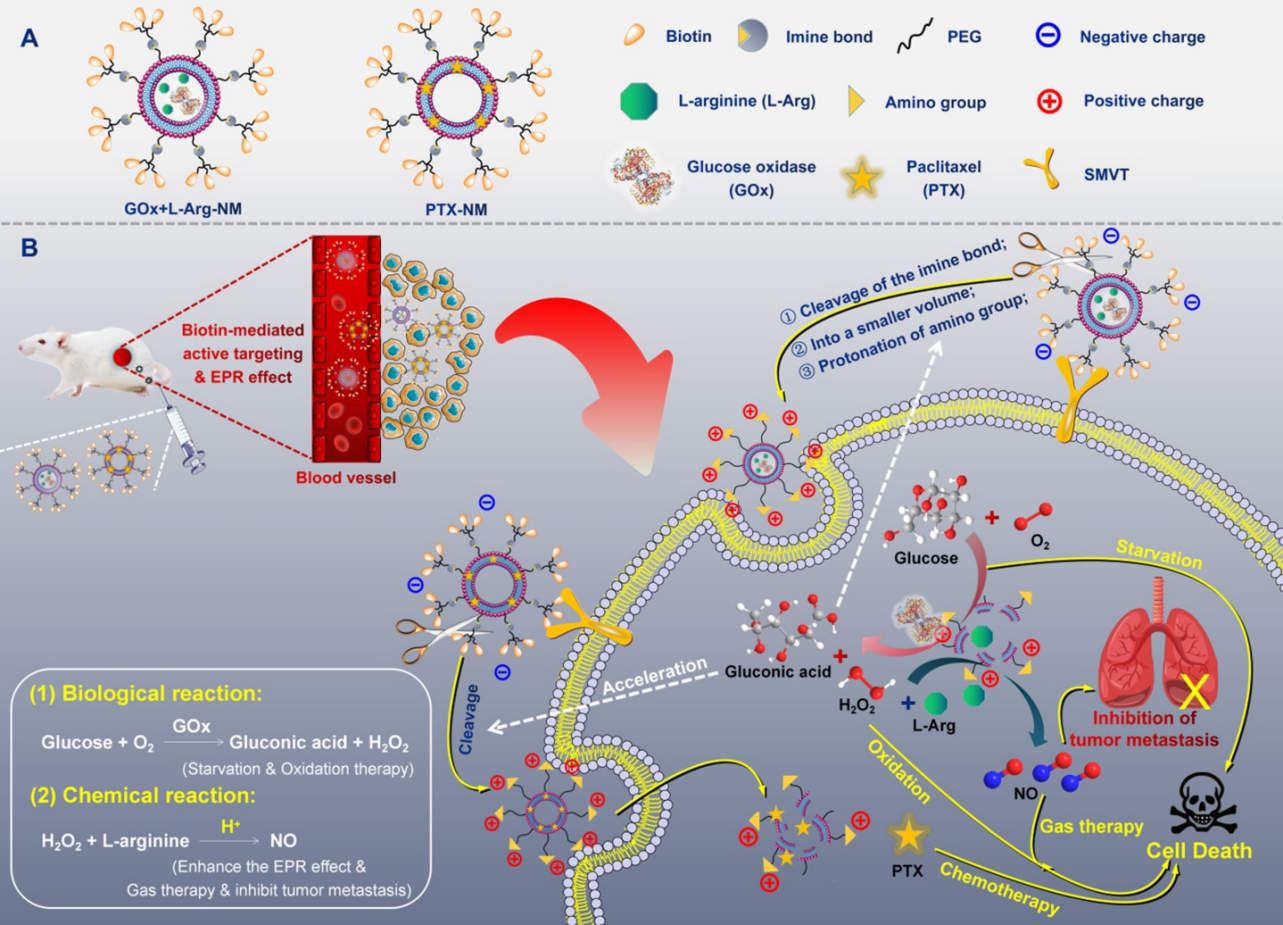
(**A**) Structural sketches of accurate programmed multifunctional cascade nano-missiles. (**B**) Schematic showing the working principles of GOx+L-Arg-NM&PTX-NM mediated self-promoted deep delivery and synergistic cascade tumor therapy. Firstly, GOx+L-Arg-NM and PTX-NM were intravenously injected and then efficiently accumulated at the tumor site due to the cooperation between biotin-mediated active targeting and the EPR effect. Subsequently, the nano-missiles could shrink their particle size and reverse surface charge from negative to positive after the acid-promoted cleavage of the imine bond, effectively transporting into the tumor cells. Under the catalysis of GOx, the intracellular glucose could be oxidized into gluconic acid and poisonous H_2_O_2_, simultaneously achieving starvation and oxidation therapy. Then, the H_2_O_2_ could further oxidize L-Arg into abundant NO, thereby efficiently inhibiting the proliferation and metastasis of tumors (NO-mediated gas therapy). More importantly, the decreased pH arising from the generated gluconic acid could in turn accelerate the cleavage of the imine bond, achieving self-promoted deep delivery. Finally, the nano-missiles could dramatically ablate tumor and restrain tumor metastasis *via* orchestrated multimodal synergistic starvation/oxidation/gas/chemotherapy.

Ammunitions, the core components of the “nano-missile (NM)”, are the pivotal factor in defeating the enemy. Finally, by equipping ammunitions on the above newly developed all-in-one nano-rocket, unprecedented multifunctional cascade nano-missiles (GOx+L-Arg-NM/PTX-NM) are successfully constructed based on the following considerations (Figure 2): (i) the TME-responsive nano-missiles can “turn off” and “turn on” cellular internalization capabilities during blood circulation and in the TNBC, respectively, with excellent security. (ii) the triple-branched biotin mediated strong active targeting and nano-associated passive targeting (EPR effect) can endow the nano-missiles excellent precise targeting ability for TNBC, maximizing their enrichment in the tumor site. (iii) When successfully arrive in the acid-TME, benzyl imine bond can respond and crack rapidly, accompanied by the biotin-PEG layer detachment and amino generation. Subsequently, the nano-missiles can shrink the particle size and reverse surface charge from negative to positive, ultimately efficient transmit into the deep tissue of TNBC and lysosomal escape can be successfully achieved. (iv) Once internalized by TNBC cells, massive “bomb-like” damage based on cascade catalysis can be triggered immediately and automatically without external stimulation. In detail, paclitaxel (PTX) can block cell proliferation by inhibiting mitotic progression, leading to mitotic and postmitotic arrest and cell death (PTX-mediated chemotherapy). GOx can effectively oxidize the intracellular glucose into gluconic acid and poisonous H_2_O_2_, achieving starvation/oxidation therapy and amplifying chemotherapy. The large amount of H_2_O_2_ produced in the previous step can promptly react with L-Arg to generate sufficient NO *in situ* 35, 36, thereby efficiently inhibiting tumor cell proliferation and metastasis (NO-mediated gas therapy). (v) More ingeniously, the cascade catalytic reactions and delivery system can promote each other, and all the components of the nano-missiles can be fully utilized. NO, generated from cascade catalysis, can produce serial vascular effects, so as to enhance the EPR effect and the oxygen supply, further promoting the rapid entry of nano-missiles into the core area of TNBC and elevating the catalytic effect of GOx. Besides, the acidic amplification of TME, attributed to the sustained gluconic acid generation during the GOx-mediated oxidation progress, can facilitate the oxidization of L-Arg into NO. Additionally, elevating acidity in the TME can also in turn accelerate the cleavage of the benzyl imine bond and protonation of amino, promoting the deep penetration of nano-missiles into tumors and realizing efficient accumulation in the core area of TNBC. In conclusion, once successfully internalized by TNBC cells, the smart multifunctional cascade nano-missiles can automatically trigger relevant cascade reactions without external stimulation, destroy the homeostasis of TME, achieve self-promoting deep penetration and retention in TNBC, deliver ammunitions to the core region of TNBC to the maximum extent, and finally realize excellent suppression of tumor proliferation and metastasis *via* orchestrated multimodal synergistic starvation/oxidation/gas/chemotherapy.

## Results and Discussion

### Design, preparation and identification of novel intelligent ligands

**Figure 3:**
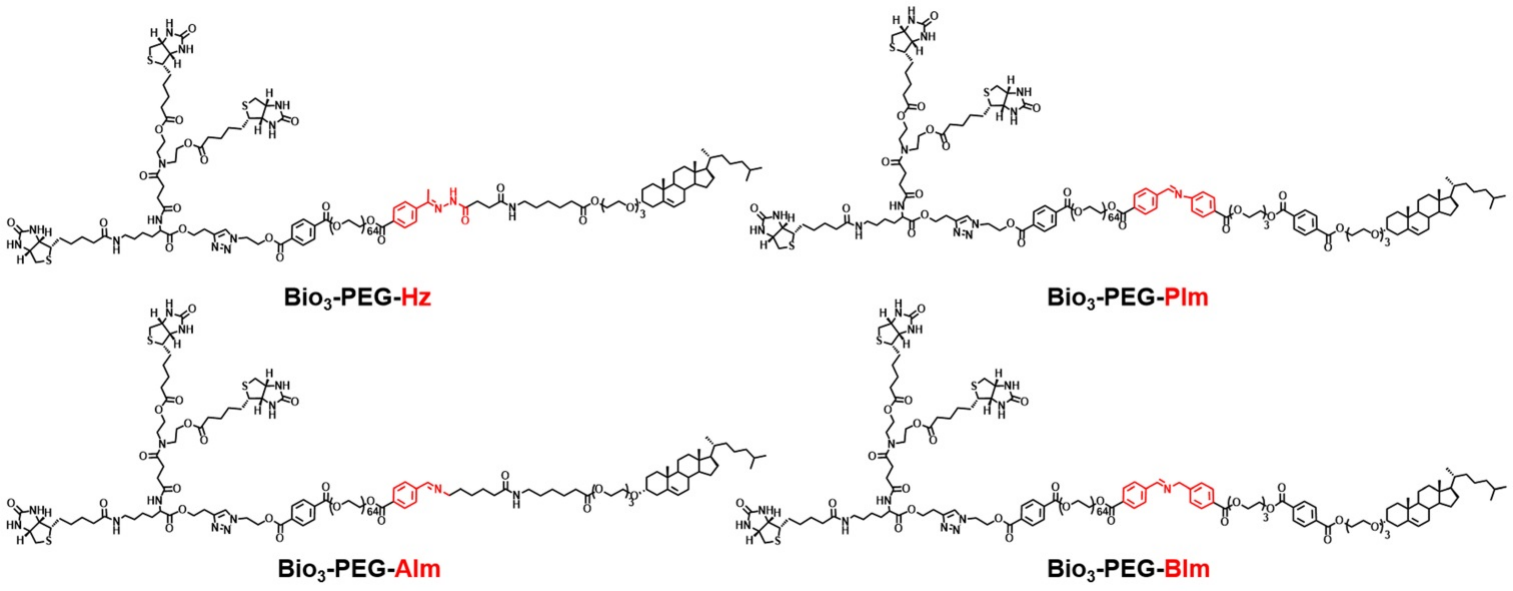
The chemical structures of ligands Bio_3_-PEG-Hz, Bio_3_-PEG-AIm, Bio_3_-PEG-PIm and Bio_3_-PEG-BIm.

In order to make the nano-missiles have more precise targeting ability for TNBC, in addition to integrating the functions of triple-branched biotin, acid-sensitive bond and long-chain PEG, we also deliberately carried out a systematic screening of the acid-sensitive bond, which should be stable at physiological pH (pH 7.4) but labile at the mildly acidic (pH 6.5) extracellular environment of tumors. Four smart ligands (Figure 3) containing different acid-sensitive bonds (Hz, AIm, PIm and BIm) were successfully designed and synthesized by chemical means for the first time, and the benzyl imine (BIm) bond showed the best TME-response capacity in the *in vitro* hydrolysis experiment (Table S1, Figure 5H and Figures S1-S3). Beyond that, the degradation rate of Bio_3_-PEG-BIm was further accelerated in the presence of GOx and glucose, achieving complete hydrolysis in only one hour (Figure 5H).

**Figure 4:**
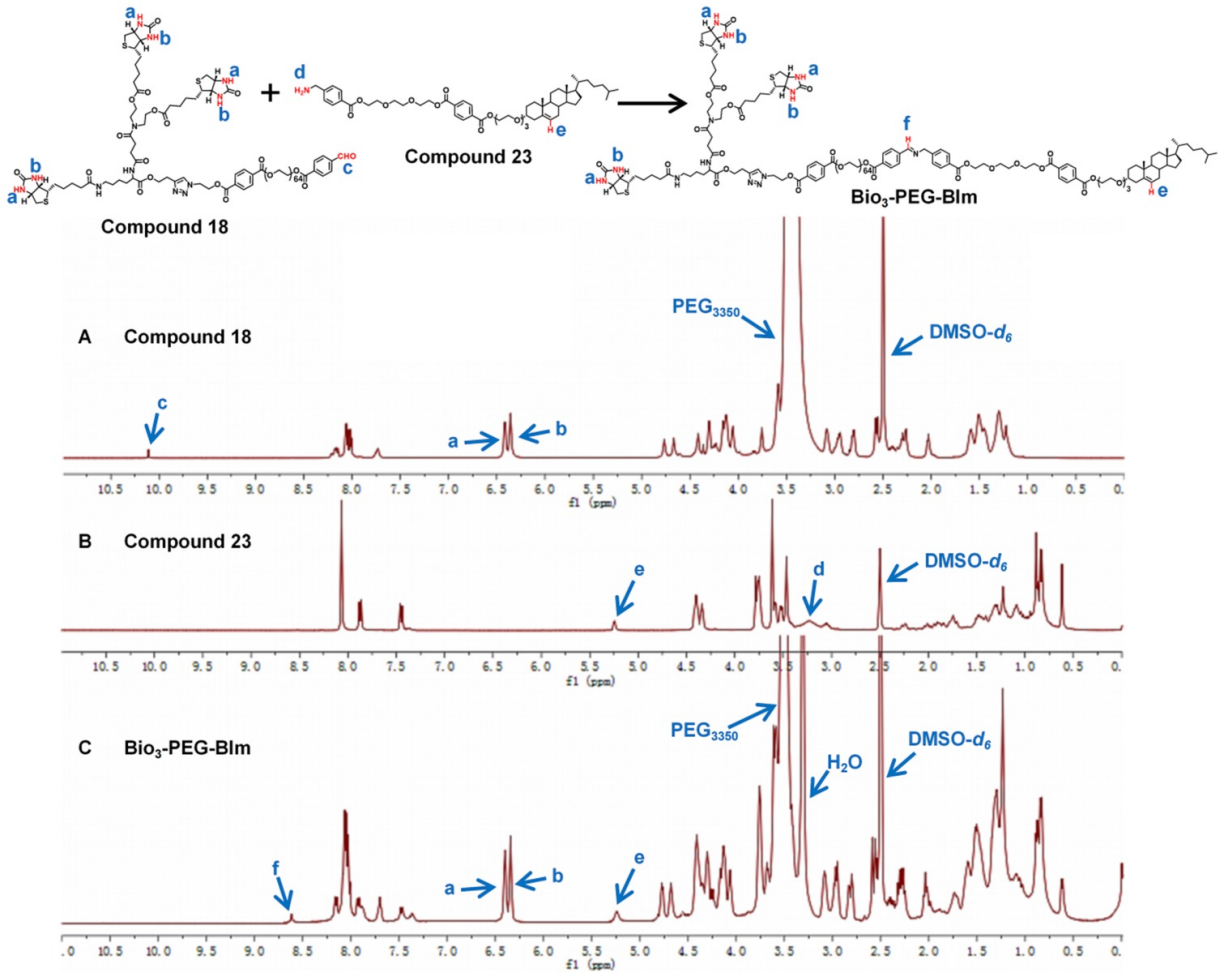
^1^H NMR spectra of compound **18** (**A**), compound **23** (**B**) and ligand Bio_3_-PEG-BIm (**C**).

The successful synthesis of Bio_3_-PEG-BIm can be confirmed by ^1^H NMR spectra (Figure 4) and high-resolution mass spectrometry data (Figure S40). In the ^1^H NMR spectra of this compound, the new signal of CH=N proton (f) appeared while the CHO proton (c) and NH_2_ proton (d) from raw materials **18** and **23** were disappeared. In addition, other characteristic protons (a, b, e) could be clearly seen in the expected regions. Moreover, in the high-resolution mass spectrum of Bio_3_-PEG-BIm, the [M+2H]^2+^ peak (m/z 2579.7344) was observed in agreement with its calculated values. The synthesis of these four ligands is described in detail in the supporting information.

### Characterization and evaluation of nano-rockets and nano-missiles

**Figure 5:**
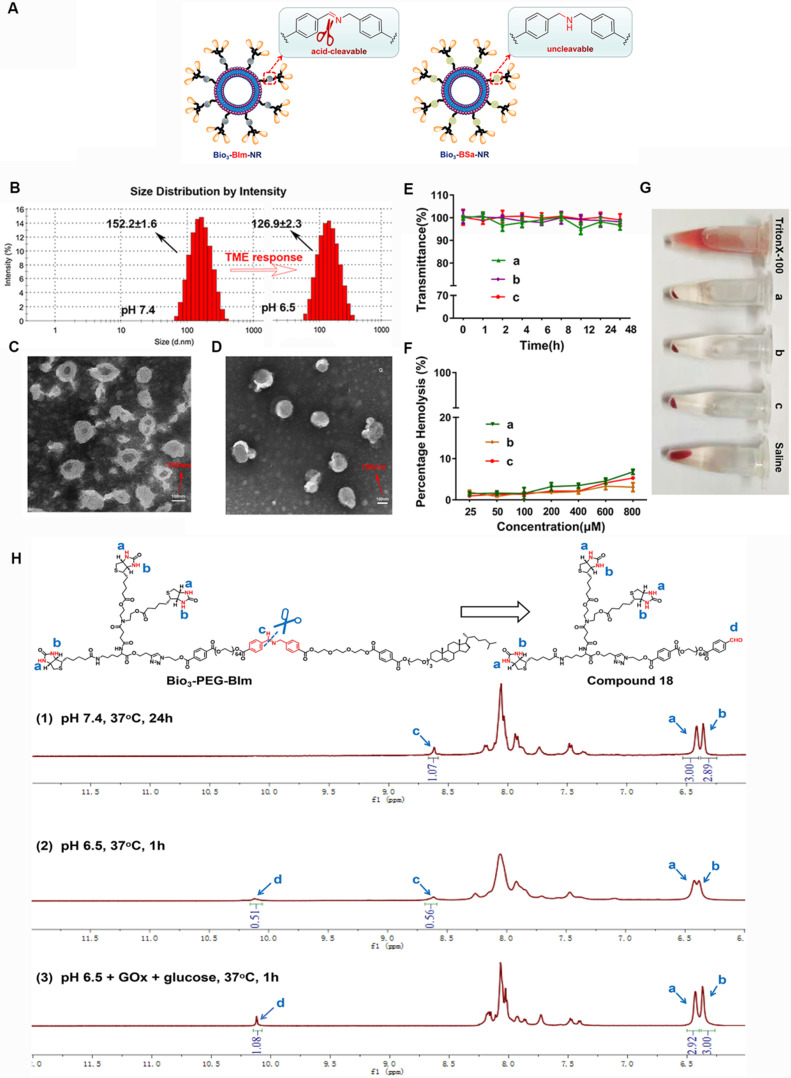
**Characterization and evaluation of nano-rockets and nano-missiles.** (**A**) Schematic diagram of Bio_3_-BIm-NR and Bio_3_-BSa-NR. (**B**) DLS size distribution of Bio_3_-BIm-NR in pH 7.4 or pH 6.5 (means ± SD, n = 3). TEM images of (**C**) PTX-NM and (**D**) GOx+L-Arg-NM. (**E**) The serum stability of nano-missiles during 48 h incubation with 50% FBS at 37 °C ((a) PTX-NM, (b) GOx+L-Arg-NM, (c) GOx+L-Arg-NM&PTX-NM) (means ± SD, n = 3). (**F**) Hemolysis ratio (%) and (**G**) photo images of red blood cells cultured with nano-missiles at various concentrations ((a) PTX-NM, (b) GOx+L-Arg-NM, (c) GOx+L-Arg-NM&PTX-NM) (means ± SD, n = 3). (**H**) ^1^H NMR spectra of Bio_3_-PEG-BIm after treatment by different conditions: (1) PBS (pH 7.4), 37 °C, 24 h; (2) PBS (pH 6.5), 37 °C, 1 h; (3) PBS (pH 6.5), GOx (100 µg/mL), glucose (1 mg/mL), 37 °C, 1 h.

To verify the response capability of Bio_3_-BIm-NR to TME, Bio_3_-BSa-NR was designed and prepared as the negative control (Figure 5A). Firstly, the pH-dependent hydrolysis of the benzyl imine bond in Bio_3_-PEG-BIm was monitored using ^1^H NMR, by treating it with different conditions (Figure 5H). Even if Bio_3_-PEG-BIm was oscillated in PBS at pH 7.4 for up to 24 h, the signal of CHO proton (c) of compound **18** was not detected, demonstrating that the benzyl imine bond was stable enough in physiological environment (Figure 5H1). At pH 6.5, the signal of CHO proton (c) appeared, while the single peak at 8.62 ppm (a) still existed, indicating partial hydrolysis of the benzyl imine bond (Figure 5H2). More importantly, by comparing the peak integral ratio of aldehyde and imine (c: a) in the NMR spectra (Figure 5H2-3), it was shown that the hydrolysis extent of Bio_3_-PEG-BIm was markedly increased by the addition of GOx and glucose. As expected, this result clearly displayed the excellent ability of GOx to accelerate the cleavage of benzyl imine bond by oxidating glucose into gluconic acid. Subsequently, we contrasted the particle size and zeta potential of the nano-rockets under different acidic conditions. Bio_3_-BIm-NR revealed a notable charge reversal from -16.7 mV to +30.8 mV and an apparent shrank hydrodynamic diameter of 25.3 nm from pH 7.4 to pH 6.5 (Figure 5B and Table S2). In contrast, Bio_3_-BSa-NR showed only slight changes (Table S2). These results suggested that the benzyl imine bond could be rapidly hydrolyzed and expose the protonated amino group, endowing Bio_3_-BIm-NR with good TME responsiveness.

Nano-missiles (GOx+L-Arg-NM and PTX-NM) were successfully prepared by the classical thin film hydration method. Whereafter, the approximately spherical shape and uniform size distribution of nano-missiles were revealed by transmission electron microscopy (TEM) and dynamic light scattering (DLS) detection (Figure 5C-D and Table S3). Hydrodynamic sizes of GOx+L-Arg-NM and PTX-NM were ≈ 174 nm and 155 nm with narrow size distributions (PDI < 0.15) and no aggregation or fusion, respectively. The nanoscale size can benefit the substantial accumulation of nano-missiles into the tumor owing to the enhanced permeability and retention (EPR) effect. According to Table S3, both GOx+L-Arg-NM and PTX-NM had strong electronegativity (-18.22 ± 0.46 and -15.02 ±0.34 mV, respectively). In addition, the encapsulation efficiency (EE) of GOx, L-Arg and PTX were determined to be 11.06%, 53.74% and 92.79%, respectively, which could guarantee the high-efficiency synergistic treatment. The above results indicated that the GOx+L-Arg-NM and PTX-NM we successfully fabricated could be used for subsequent studies, such as cellular internalization and *in vivo* distribution. No visible aggregation or precipitates was observed in the GOx+L-Arg-NM or PTX-NM group after 48 h incubation with FBS, whose transmittance values were above 95% and hardly changed, indicating the satisfactory serum stability (Figure 5E). Moreover, no detectable hemolytic reaction was observed even when the nano-missiles concentration was up to 800 μM (Figure 5F). Furthermore, the photo images revealed that the structure of the blood erythrocyte membrane had little changes and no cell lysis of erythrocytes, which were similar to that in saline (pH 7.4, Figure 5G). To sum up, all of the above results demonstrated that both GOx+L-Arg-NM and PTX-NM were very stable in physiological solution, which suggesting that *in vivo* utilization of the nano-missiles would be effective.

### Cascade catalysis of GOx+L-Arg-NM remodeled the tumor microenvironment

**Figure 6:**
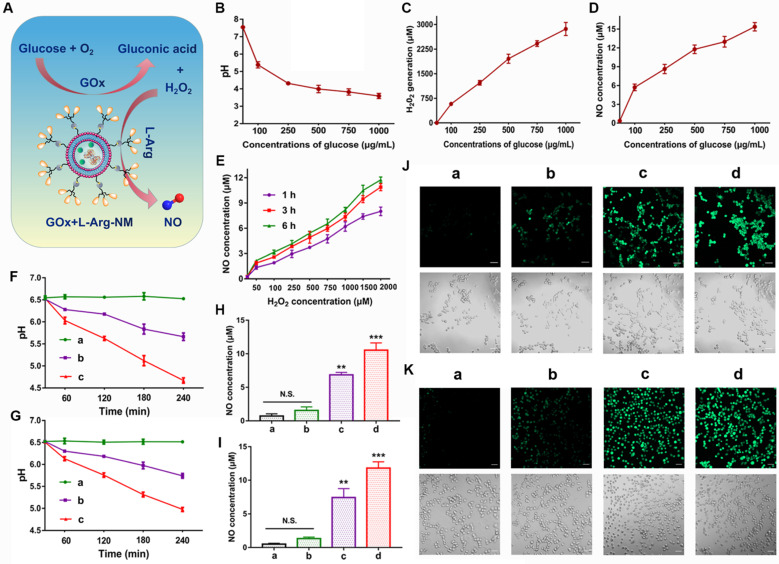
**Remodeling the tumor microenvironment by GOx+L-Arg-NM.** (**A**) Schematic illustration of GOx+L-Arg-NM-mediated cascade catalysis. (**B**) Changes of the pH values in glucose solutions containing GOx and L-Arg (means ± SD, n = 3). Generated H_2_O_2_ levels (**C**) and NO levels (**D**) in glucose solutions containing GOx and L-Arg (means ± SD, n = 3). (**E**) Amount of NO produced by L-Arg at different concentrations of H_2_O_2_ (means ± SD, n = 3). Time-dependent changes in the pH values of 4T1 cells (**F**) and MDA-MB-231 cells (**G**) treated with different nano-missiles ((a) control, (b) GOx+L-Arg-NM (GOx: 10 µg/mL, glucose: 0 mg/mL), (c) GOx+L-Arg-NM (GOx: 10 µg/mL, glucose: 1 mg/mL)) (means ± SD, n = 3). NO generation in 4T1 cells (**H**) and MDA-MB-231 cells (**I**) after incubation with different nano-missiles for 6 h ((a) control, (b) PTX-NM, (c) GOx+L-Arg-NM, (d) GOx+L-Arg-NM&PTX-NM) (means ± SD, n = 3, ** indicates p < 0.01, *** indicates p < 0.001, N.S. indicates no significant difference). Detection of NO in 4T1 cells (**J**) and MDA-MB-231 cells (**K**) using DAF-FM DA fluorescent probe. ((a) control, (b) PTX-NM, (c) GOx+L-Arg-NM, (d) GOx+L-Arg-NM&PTX-NM) (means ± SD, n = 3). The scale bar = 50 µm.

As an enzyme catalyst, GOx can efficiently oxidize the intracellular glucose into gluconic acid and H_2_O_2_, which will continue to react with L-Arg to generate sufficient NO *in situ* (Figure 6A). The unique features of TME establish an equilibrium state of elevated ROS and over-expressed antioxidant mechanisms in comparison to the normal cells. High concentrations of H_2_O_2_ (>25 μM) can efficiently kill tumor cells while sensitizing them to antitumor drugs 37-40. Similarly, the antitumor effect of NO is closely related to its concentration and duration in tumor cells. High concentrations of NO (>1 μM) can directly kill tumor cells and suppress metastasis through multiple pathways 41, 42. When the glucose solution was incubated with GOx and L-Arg, large amounts of H_2_O_2_ and gluconic acid (reflected by pH) were successfully detected (Figure 6B-C), indicating the high catalytic efficiency of GOx. In detail, abundant H_2_O_2_ (578-2865 μmol/L) was produced with the increase of glucose concentration (100-1000 µg/mL) (Figure 6C). And a dramatic pH decline from 7.54 to 3.59 was appeared (Figure 6B). Meanwhile, glucose-dependent NO release was clearly observed in the solution (Figure 6D), because the generated NO amount relied on the H_2_O_2_/H^+^ concentration, which was related to the added glucose concentration. In addition, we found that the amount of NO generated was proportional to the concentration of H_2_O_2_ and increased with the extension of time (Figure 6E). Notably, as illustrated in Figure 6F-G, a dramatic pH drop (from 6.53 to 4.67, 6.51 to 4.98) happened in tumor microenvironment when 4T1/MDA-MB-231 cells incubated with GOx+L-Arg-NM as exposed time extended. Beyond that, the PTX-NM group only produced marginal amounts of NO (Figure 6H-K). In contrast, higher amounts of NO were generated in cells treated with GOx+L-Arg-NM (Figure 6H-I). More and brighter fluorescence was observed in GOx+L-Arg-NM-treated cells, which further confirmed that the intracellular NO concentration of GOx+L-Arg-NM was higher than that of PTX-NM (Figure 6J-K). Furthermore, the GOx+L-Arg-NM&PTX-NM group showed significantly stronger fluorescence and higher NO levels than others (Figure 6H-K). Taken together, by co-loading GOx and L-Arg into Bio_3_-BIm-NR, GOx+L-Arg-NM could automatically trigger the cascade catalysis without external stimulation to generate sufficient amounts of H_2_O_2_, gluconic acid and NO, further successfully remodeling the tumor microenvironment.

### Cascade catalysis of GOx+L-Arg-NM remitted hypoxia and promoted rapid entry of nano-missiles into the core area of TNBC

**Figure 7:**
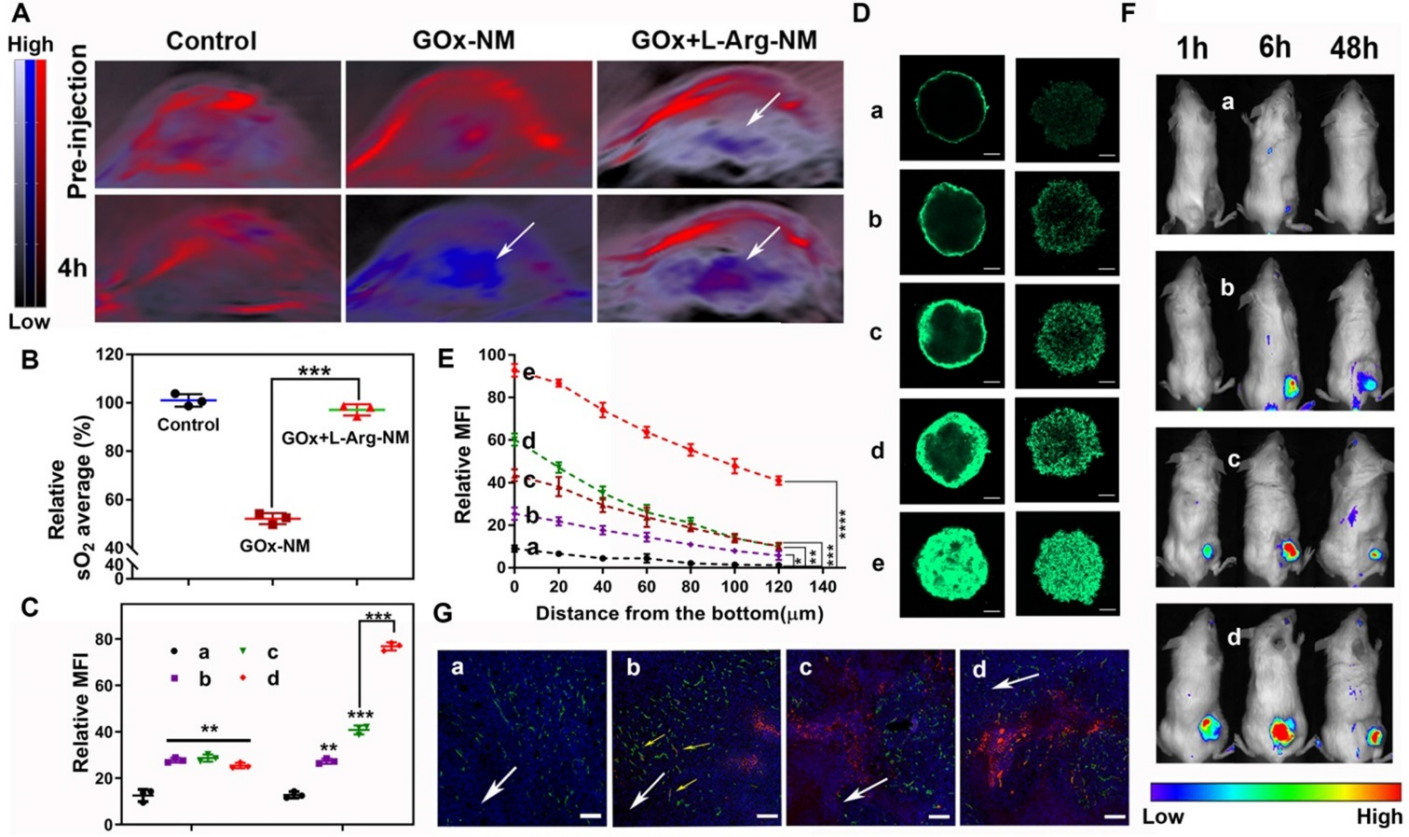
** GOx+L-Arg-NM remitted hypoxia and promoted rapid entry of nano-missiles into the core area of TNBC.** (**A**) PA images of 4T1 tumor blood oxygen saturation (sO_2_) levels with injection of PBS, GOx-NM or GOx+L-Arg-NM at pre and 4 h. The color scale was calculated from the PA signal ratios between de-oxygenated (750 nm, blue) and oxygenated hemoglobin (850 nm, red). (**B**) Corresponding quantitative analysis (relative sO_2_ average (%)) of the PA images in tumor regions (means ± SD, n = 3, *** indicates p < 0.001). (**C**) The quantitative cellular internalization of 4T1 cells after incubation with nano-rockets at pH 7.4 (left) or pH 6.5 (right), respectively ((a) CFPE-LF-NR, (b) CFPE-Bio_3_-BSa-NR, (c) CFPE-Bio_3_-BIm-NR, (d) CFPE-Bio_3_-BIm-NR&GOx+L-Arg-NM) (means ± SD, n = 3, ** indicates p < 0.01, *** indicates p < 0.001). (**D**) The fluorescence distribution of 4T1 and MDA-MB-231 tumor spheroids treated with different nano-missiles at pH 6.5, respectively ((a) CFPE-LF-NR, (b) CFPE-Bio_3_-BSa-NR, (c) CFPE-Bio_3_-BSa-NR&GOx+L-Arg-NM, (d) CFPE-Bio_3_-BIm-NR, (e) CFPE-Bio_3_-BIm-NR&GOx+L-Arg-NM) (n = 3, the scale bar = 100 µm). (**E**) The semi-quantitative intensity of nano-rockets in 4T1 tumor spheroids ((a) CFPE-LF-NR, (b) CFPE-Bio_3_-BSa-NR, (c) CFPE-Bio_3_-BSa-NR&GOx+L-Arg-NM, (d) CFPE-Bio_3_-BIm-NR, (e) CFPE-Bio_3_-BIm-NR&GOx+L-Arg-NM) (means ± SD, n = 3, * indicates p < 0.05, ** indicates p < 0.01, *** indicates p < 0.001, **** indicates p < 0.0001 versus CFPE-LF-NR). (**F**) Representative *in vivo* fluorescence images of the 4T1 tumor xenografts bearing mice and (**G**) distribution in the deep region of the tumor slices after intravenous injection with (a) DiD-LF-NR, (b) DiD-Bio_3_-BSa-NR, (c) DiD-Bio_3_-BIm-NR, (d) DiD-Bio_3_-BIm-NR&GOx+L-Arg-NM (n = 6). Green represents CD34, red represents DiD, blue represents DAPI and the scale bar = 100 µm.

NO, a multifunctional gaseous mediator, plays extensive roles pathologically and physiologically. Peculiarly in vascular functions, NO can enhance vascular permeability and perfusion, consequently replenishing oxygen to the tumor site. The intratumoral sO_2_ levels were recorded by employing the non-invasive PA imaging technique to validate the vascular functions of NO generated from cascade catalysis. As illustrated in Figure 7A-B, the sO_2_ average total (%) showed ~47.8% significantly dropping within 4 h post *i.v.* injection of GOx-NM, clearly evidencing the tumor hypoxia status was dramatically enhanced. The exacerbation of hypoxia by GOx-NM indicated the effective consumption of glucose and oxygen within the tumor by the starvation therapy. No obvious change was observed in PBS group. Surprisingly, when GOx+L-Arg-NM was given, intratumoral sO_2_ level increased to 97.1% compared with GOx-NM, which was ascribed to the endogenous NO-generation. It is worth mentioning that Oxy-Hemo signal with the GOx+L-Arg-NM injection appeared in the deep region of tumors, indicating blood perfusion increased in core aspect. To sum up, GOx would exhaust the oxygen of the tumor survival microenvironment, and NO produced continuously could efficiently and specifically increase the delivery of intratumoral oxygen which was material for the oxidation process. Additionally, the feasibility of GOx+L-Arg-NM could automatically trigger the cascade catalysis within the tumour was indeed manifested.

Comparative cellular uptake of various nano-rockets was quantificationally analyzed by flow cytometry. Based on the enhanced active targeting ability of the triple-branched biotin fragment to TNBC, the fluorescence signal in cells cultured with CFPE-Bio_3_-BSa-NR or CFPE-Bio_3_-BIm-NR was comparatively stronger compared to CFPE-LF-NR in a neutral environment (Figure 7C and Figure S4A). Moreover, when the pH of the surrounding environment changed from 7.4 to 6.5, cells demonstrated a significant shift in the mean fluorescence intensity treated with CFPE-Bio_3_-BIm-NR as compared to that of the cell populations treated with CFPE-Bio_3_-BSa-NR. Satisfyingly, maximum nano-rockets were delivered to the cells when both CFPE-Bio_3_-BIm-NR and GOx+L-Arg-NM were jointly employed. Particularly, the fluorescence intensity of CFPE-Bio_3_-BIm-NR&GOx+L-Arg-NM was approximate 6-fold and 1.9-fold compared with that for CFPE-LF-NR and CFPE-Bio_3_-BIm-NR in both 4T1 and MDA-MB-231 cells within 1 h (Figure 7C and Figure S4A). The results were further corroborated by CLSM images, and the co-localized with lysosomes indicated that CFPE-Bio_3_-BIm-NR&GOx+L-Arg-NM achieved the optimal lysosomal-escaping (Figure S4C-D). Next, the penetration ability of formulations was evaluated on 3D tumor spheroids (Figure 7D-E, Figure S4B and Figure S4E-F). Not surprisingly, the penetration of the CFPE-Bio_3_-BSa-NR group displayed slightly increased contrasted with CFPE-LF-NR, and most of them were excluded from the initial of collagen matrix, suggesting CFPE-Bio_3_-BSa-NR difficult to diffuse into the deep regions because of the large size and negative charge. In striking contrast, CFPE-Bio_3_-BIm-NR displayed more fluorescent intensity in spheroid than CFPE-Bio_3_-BSa-NR put down to the cleavage of benzyl imine bond. After administered in combination with GOx+L-Arg-NM, CFPE-Bio_3_-BSa-NR&GOx+L-Arg-NM distributed more in the core of tumor spheroid compared CFPE-Bio_3_-BSa-NR, whereas the fluorescent signal still weak, illustrating NO could indeed assist large nanoparticles penetrate deeply, but the efficiency was not satisfactory. Undoubtedly, CFPE-Bio_3_-BIm-NR&GOx+L-Arg-NM displayed deeper diffusion and stronger intensity than other groups, which further implied that the synergistic effect of triple-branched biotin fragment, TME-responsive benzyl imine bond and NO could notably facilitate the deep penetration process (Figure 7D-E, Figure S4B and Figure S4E-F).

BALB/c tumor-bearing mice were utilized for imaging after the systemic administration of DiD-labeled nano-rockets. Figure 7F and Figure S5A showed the real-time images of *in vivo* distribution at predetermined points after tail vein injection. Taking advantage of the prolonged systemic circulation, triple-branched biotin fragment-mediated potent targeting effect and acid sensitive bond, the DiD-Bio_3_-BIm-NR could more effectively accumulated in the tumor tissues in a highly specific manner. As expected, it was perspicuously observed that the fluorescence signal of the DiD-Bio_3_-BIm-NR&GOx+L-Arg-NM group in the tumor site was notably stronger than that of the other groups from 1 h to 48 h, which was favorable for precise tumor therapy (Figure S5A-B). According to the semiquantitative data (Figure S5C), the DiD-Bio_3_-BIm-NR&GOx+L-Arg-NM group showed fluorescence intensity at the excised tumor site was 1.97-, 4.40-, and 12.17- fold higher than that of DiD-Bio_3_-BIm-NR, DiD-Bio_3_-BSa-NR and DiD-NR (p < 0.001), respectively. Likewise, the DiD-Bio_3_-BIm-NR&GOx+L-Arg-NM group showed a remarkable fluorescence intensity and diffused more deeply into the tumor that were far away from the anti-CD34-stained neurovascular blood vessel (Figure 7G). In contrast, the DiD-Bio_3_-BSa-NR group was mainly confined to the blood vessel or peripheral area. These above results clearly elucidated that DiD-Bio_3_-BIm-NR could efficiently gather in tumor relied on triple-branched biotin fragment specifically binding SMVT, followed by entering into cells through unspecific electrostatic adsorption, and further facilitated by gluconic acid plus NO release. Moreover, disruption of tumor microenvironment homeostasis (NO and glucose acid generating from cascade catalysis) could be devoted to superior EPR effect and efficiently enhance the ability of DiD-Bio_3_-BIm-NR&GOx+L-Arg-NM to transport to the core region of the tumor even with the existence of high interstitial hypertension.

### Massive “bomb-like” attacks triggered by nano-missiles synchronously suppressed TNBC proliferation and metastasis

**Figure 8:**
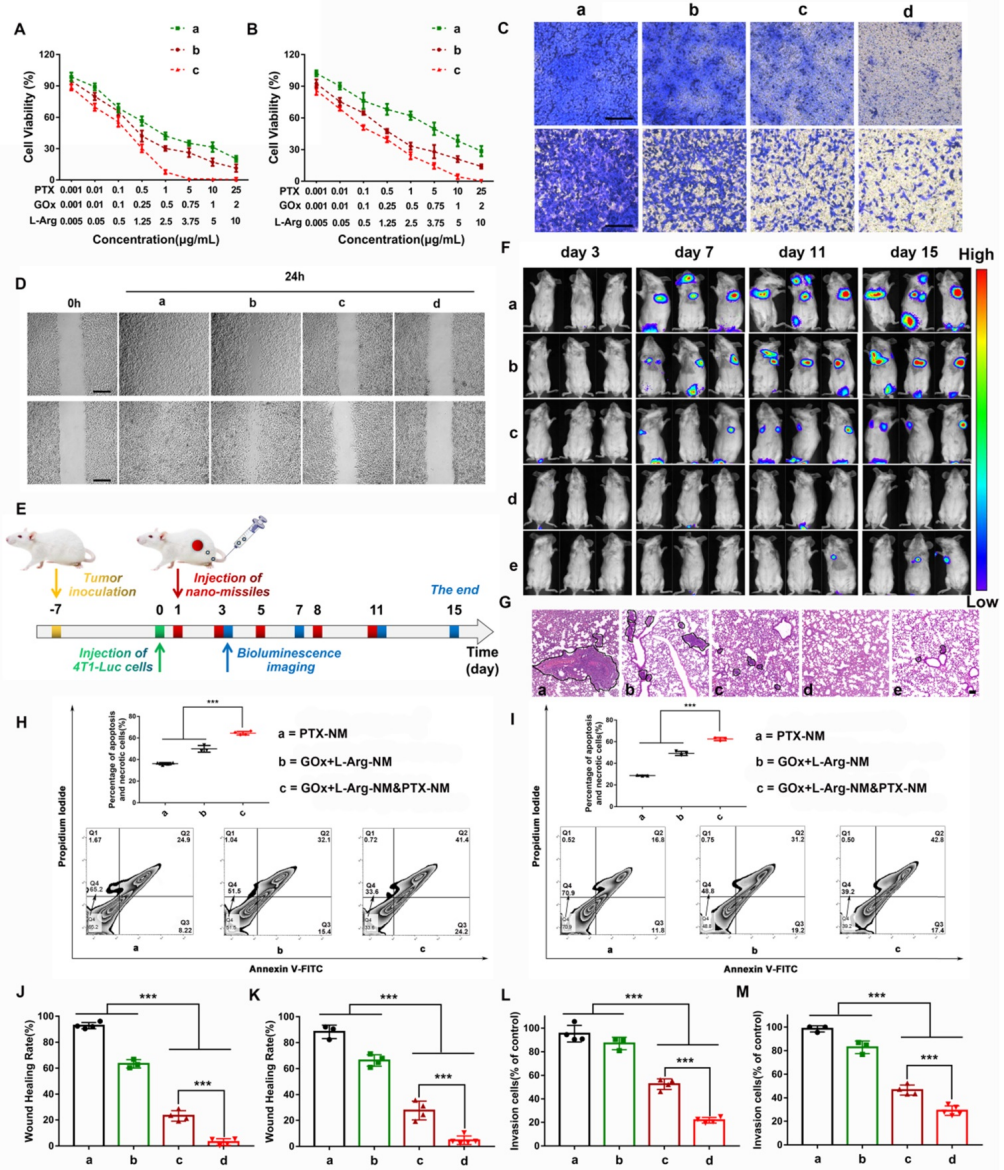
** Nano-missiles efficiently suppressed the proliferation and metastasis of TNBC.** Viabilities of the 4T1 cells (**A**) and MDA-MB-231 cells (**B**) after 24 h of incubation with different nano-missiles ((a) PTX-NM, (b) GOx+L-Arg-NM, (c) GOx+L-Arg-NM&PTX-NM) (means ± SD, n = 6). Images (**C**) and quantitative analysis of invaded 4T1 cells (**L**), MDA-MB-231 cells (**M**) after separate incubation with (a) PBS, (b) PTX-NM, (c) GOx+L-Arg-NM, or (d) GOx+L-Arg-NM&PTX-NM for 24 h. The invaded cells were stained with crystal violet (means ± SD, n = 3, *** indicates p < 0.001). The scale bar = 200 µm. Images (**D**) and healing rates of 4T1 cells (**J**) and MDA-MB-231 (**K**) cells after separate incubation with (a) PBS, (b) PTX-NM, (c) GOx+L-Arg-NM, or (d) GOx+L-Arg-NM&PTX-NM for 24 h (means ± SD, n = 3, *** indicates p < 0.001). The scale bar = 200 µm. (**E**) Pulmonary metastasis mice models were *i.v*. administrated with different nano-missiles on day 1, 3, 5, 8 and 11. Bioluminescence imaging were taken on day 3, 7, 11 and 15. (**F**) Pulmonary metastasis growth inhibition measured by BLI and (**G**) representative images from H&E assays of excised lungs ((a) PBS, (b) PTX-NM, (c) GOx+L-Arg-NM, (d) GOx+L-Arg-NM&PTX-NM (PTX = 3 mg/kg), (e) GOx+L-Arg-NM&PTX-NM (PTX = 1 mg/kg)) (means ± SD, n = 6). The dark purple parts indicated metastatic nodules. The scale bar = 100 µm. Flow cytometry analysis and the apoptotic proportion of 4T1 cells (**H**) and MDA-MB-231 cells (**I**) after incubation with (a) PTX-NM, (b) GOx+L-Arg-NM, (c) GOx+L-Arg-NM&PTX-NM (means ± SD, n = 3, *** indicates p < 0.001).

Encouraged by the excellent cascade catalysis effect of the GOx+L-Arg-NM, *in vitro* therapeutic efficacy was evaluated. The MTT results showed that GOx+L-Arg-NM&PTX-NM exhibited a notably improved considerable cytotoxicity in comparison with PTX-NM, especially in MDA-MB-231 cells. Of note, the IC_50_ values of PTX in the GOx+L-Arg-NM&PTX-NM group were dramatically down to 0.08 μg/mL (4T1) or 0.09 μg/mL (MDA-MB-231), whereas the IC_50_ of PTX-NM were 0.89 μg/mL for the 4T1 cells or 3.27 μg/mL for the MDA-MB-231 cells (Figure 8A-B and Table S4). It is known that NO can react rapidly with superoxide to form peroxynitrite, which acts as an inducer of cytotoxicity and apoptosis. In agreement with the MTT assay results, Annexin V-FITC/PI staining results revealed that cells treated with GOx+L-Arg-NM showed significant apoptosis as more than 50% of cells were in apoptosis and necrotic (Figure 8H-I). Notably, a synergistic enhancement in therapeutic efficiency by treatment with GOx+L-Arg-NM&PTX-NM could be clearly observed (Figure 8H-I). The percentage of apoptosis and necrotic cells against 4T1 cells was 64.4 ± 1.65%, and 62.4 ± 1.44% for MDA-MB-231. Therefore, benefiting from the synergistic effects of chemotherapy, GOx-mediated starvation, H_2_O_2_-excited oxidation and NO-mediated gas therapy, GOx+L-Arg-NM&PTX-NM could cause a satisfactory tumor inhibition effect. Using similar experimental methods, we have also evaluated the cellular internalization and antitumor activity of the GOx+L-Arg-NM&PTX-NM on MCF7 cells and consistently observed that GOx+L-Arg-NM&PTX-NM could be efficiently internalized by the MCF7 cells and restrain their growth (Figure S6A-H).

Metastases, which induced lesions in other tissues and organs, remains the main cause of TNBC-related mortality. Studies showed that sufficient NO could suppress invasion and metastasis through down regulating the expression of cell adhesion factors on endothelial cells, inhibiting the expression of matrix metalloproteinases (MMPs) and preventing platelet adhesion 43-46. To verify that NO released from cascade catalysis could suppress the metastasis, a series of assays were conducted. 4T1 or MDA-MB-231 cells, which exhibit high motility and strong metastatic properties, were used to investigate the anti-metastatic effect of nano-missiles. For the wound-healing assay, Figure 8D showed that scratch gaps in control groups almost recovered after 24 h. The slight suppression was observed in PTX-NM groups. Notably, the gap-healing rate decreased to approximately 23.2% or 27.7% after treatment with GOx+L-Arg-NM in 4T1 or MDA-MB-231 cells, respectively (Figure 8J-K). When given GOx+L-Arg-NM&PTX-NM, the cell motility was further significantly inhibited, with a migration distance ratio of only about 4% (Figure 8J-K). In addition, the invasion inhibitory effect was then assessed utilizing cell invasion assays. The results exhibited the same trend as the wound healing assay (Figure 8C). Majority of cells could invade across the Matrigel-coated membrane in PBS. Compared with the PBS group, the slight inhibition of invasion was observed in PTX-NM group with only reducing the invasion rate to 82.8%. We noticed that after treatment with GOx+L-Arg-NM, the invasion ability of MDA-MB-231 cells was clearly restrained with the rate of 46.6%, which attributed to NO produced from cascade catalysis (Figure 8L-M). Additionally, the GOx+L-Arg-NM&PTX-NM group exhibited optimal inhibition of invasion, wherein the invasion rate reduced to 21.8% or 29.1% in 4T1 or MDA-MB-231 cells, respectively (Figure 8L-M), which indicated that combination of GOx+L-Arg-NM and PTX-NM could achieve considerable anti-migration effects and reduce the risk of tumor metastasis.

As Figure 8F shows, 7 days after the intravenous injection of 4T1-Luc cells, the fluorescence intensity of metastases in the lungs of PBS-treated mice was apparent and notably grew with time. The treatment of PTX-NM group reduced tumor metastasis just to a certain extent, suggesting insufficient to inhibit the metastases. By constrast, GOx+L-Arg-NM performed notably metastasis-inhibited effect, ascribed to the released NO contributing to the metastasis inhibition, whereas the lung metastasis still grew slowly with time. More importantly, nearly no bioluminescence signal could be detected for mice after treatment with GOx+L-Arg-NM&PTX-NM (PTX = 3 or 1 mg/kg, GOx = 1 mg/kg). Further, mice with only PBS treatment showed obvious lung metastasis with significant nodules displayed in the H&E-stained images, while fewer metastatic nodules were observed of GOx+L-Arg-NM&PTX-NM groups (Figure 8G). All these results demonstrated GOx+L-Arg-NM&PTX-NM endowed with potent anti-metastasis effect which ascribed to the function of released NO and the effectiveness of combinational therapy.

**Figure 9:**
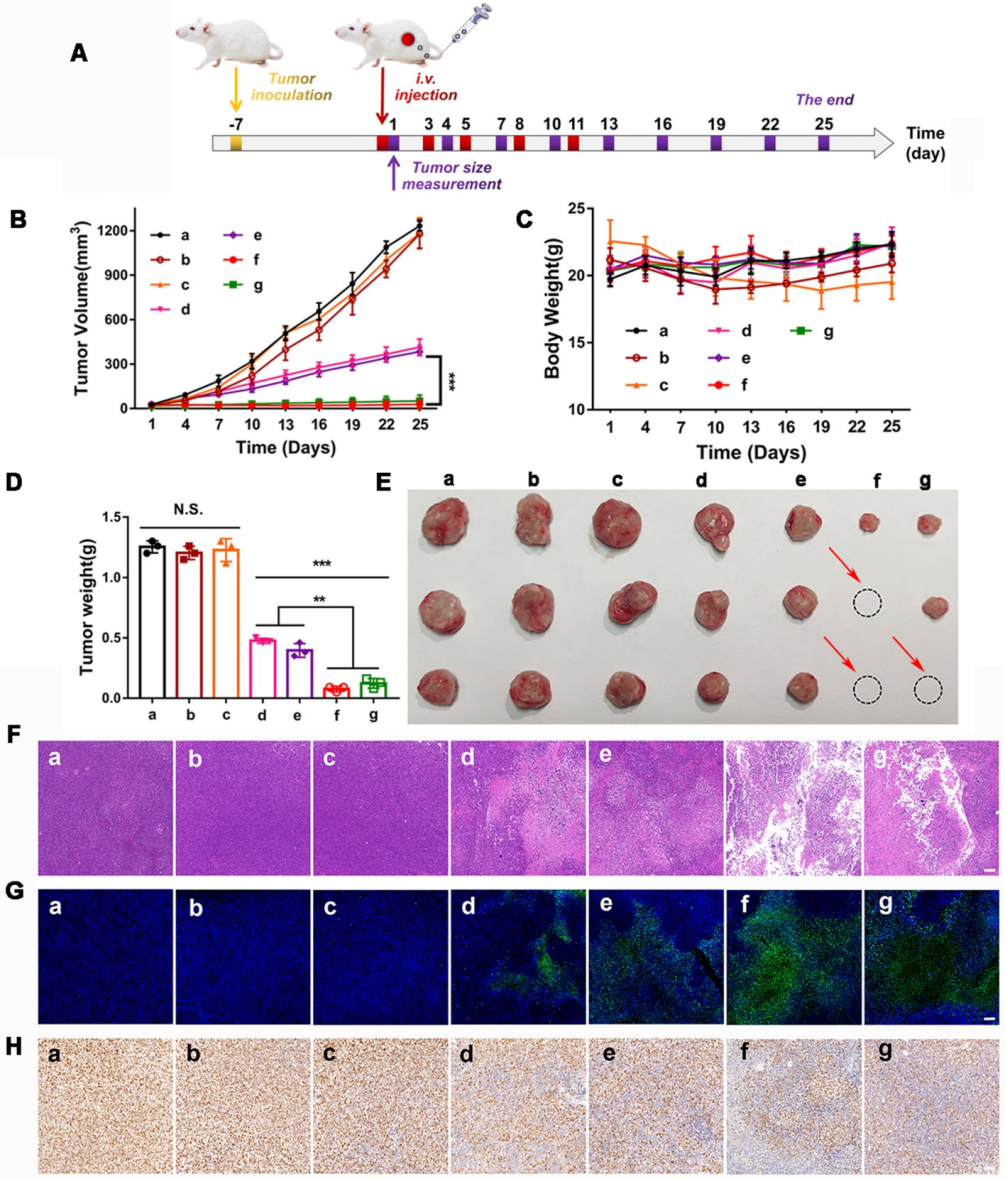
***In vivo* therapeutic efficacy in 4T1 tumor bearing mice.** (**A**) The BALB/c mice bearing 4T1 tumors were* i.v.* administrated with different nano-missiles on day 1, 3, 5, 8 and 11. The tumor size was monitored every 3 days from day 1. (**B**) Tumor volume changes in 4T1 tumor-bearing mice ((a) PBS, (b) Free PTX, (c) Free GOx+L-Arg, (d) PTX-NM, (e) GOx+L-Arg-NM, (f) GOx+L-Arg-NM&PTX-NM (PTX = 3 mg/kg), (g) GOx+L-Arg-NM& PTX-NM (PTX = 1 mg/kg)) (means ± SD, n = 6, *** indicates p < 0.001). (**C**) Changes in body weight over the treatment ((a) PBS, (b) Free PTX, (c) Free GOx+L-Arg, (d) PTX-NM, (e) GOx+L-Arg-NM, (f) GOx+L-Arg-NM&PTX-NM (PTX = 3 mg/kg), (g) GOx+L-Arg-NM&PTX-NM (PTX = 1 mg/kg)). (**D**) Excised tumor weight on day 25 ((a) PBS, (b) Free PTX, (c) Free GOx+L-Arg, (d) PTX-NM, (e) GOx+L-Arg-NM, (f) GOx+L-Arg-NM&PTX-NM (PTX = 3 mg/kg), (g) GOx+L-Arg-NM&PTX-NM (PTX = 1 mg/kg)) (means ± SD, ** indicates p < 0.01, *** indicates p < 0.001, N.S. indicates no significant difference). (**E**) Representative photographic image of tumor tissues obtained on day 25. Representative H&E images (**F**), typical TUNEL staining images (**G**) and Ki67 staining images (**H**) of the tumor slices ((a) PBS, (b) Free PTX, (c) Free GOx+L-Arg, (d) PTX-NM, (e) GOx+L-Arg-NM, (f) GOx+L-Arg-NM&PTX-NM (PTX = 3 mg/kg), (g) GOx+L-Arg-NM&PTX-NM (PTX = 1 mg/kg)). The scale bar = 200 µm.

The *in vivo* synergistic therapeutic efficacy of GOx+L-Arg-NM&PTX-NM was assessed against the 4T1 tumor-bearing mice. As depicted in Figure 9B, tumors in the free PTX or GOx+L-Arg group exhibited negligible tumor suppression and undeterred growth (with TIR of 4.4% or 4.1%), which was in accordance with the limited tumor accumulation of free drugs. In comparison, PTX-NM or GOx+L-Arg-NM exhibited relatively greater therapeutic ability, benefiting from the sufficient accumulation in the tumor tissue, of which the final tumor volume has been reduced to 431.9 mm^3^ and 383.1 mm^3^. However, the therapeutic efficacy of monotherapy was still quite limited and difficult to achieve the desired effect. Both tumor volume (Figure 9B) and photo (Figure 9E) results showed that mice administrated of GOx+L-Arg-NM&PTX-NM displayed the satisfactory tumor suppression effect with superior TIR presented to be 97.7% at a PTX dose of 3 mg/kg or 95.9% when 1 mg/kg. Hence, the GOx+L-Arg-NM&PTX-NM groups possessed the most pronounced tumor inhibition and even caused tumor ablation, showing extreme antitumor efficacy attributed to the starvation/ROS-/NO/chemo-mediated combination therapy. Tumor weight analysis also revealed a similar pattern (Figure 9D). Additionally, no significant variations in the body weights of mice were observed among various groups except Free GOx+L-Arg groups which gradually decreased throughout the treatment period, demonstrating the negligible side effects induced by nano-missiles (Figure 9C). Meanwhile, the H&E, TUNEL, and Ki67 staining of tumor slices further elucidated and confirmed the superior antitumor effect of the GOx+L-Arg-NM&PTX-NM groups (Figure 9F-H). More precisely, the most significant nuclear fragmentation from H&E staining, the most severe tumor cells apoptosis from TUNEL staining and the minimum expression level of the proliferative marker from Ki67 assay were observed in the tumor of GOx+L-Arg-NM&PTX-NM (PTX = 3 mg/kg, GOx = 1 mg/kg) group. These results adequately validated that the GOx+L-Arg-NM could establish a satisfactory multimodal synergistic starvation/oxidation/NO gas therapy, further efficiently cause irreversible damage to tumor cells and amplify the effect of chemotherapy. Better yet, the generated NO and gluconic acid from cascade catalysis could effectively increase tumor accumulation of GOx+L-Arg-NM&PTX-NM, which further enhance the therapeutic effect.

Comparing to the tumor cell lines, it was found that the CFPE-Bio_3_-BIm-NR or CFPE-Bio_3_-BIm-NR&GOx+L-Arg- NM internalization by the L02 cells were significantly lower (Figure S7E-F), further validating the pronounced tumor-selectivity effect of the triple-branched biotin fragment. Additionally, the cell survival rate was generally above 90% in the normal dose range, indicating a cytocompatibility of the nano-rockets substrate (Figure S7A-D). A good biocompatibility and low side effects *in vivo* are also necessary prerequisites and guarantee for the efficacy of drugs. Except free GOx+L-Arg group, H&E staining of the main organs (liver, heart, lung, spleen and kidney) of mice revealed no noticeable pathological abnormalities in the nano-missiles groups, confirming satisfactory biocompatibility and biosecurity (Figure S8). All these results demonstrated the merits of nano-missiles-based drug delivery system for enhanced biosafety, and GOx+L-Arg-NM&PTX-NM could be a promising candidate to serve as a tumor nanotherapeutic agent.

## Conclusion

In summary, based on the concept of maximizing the function of a single ligand, a bran-new multifunctional ligand (Bio_3_-PEG-BIm), which simultaneously possessed capabilities of accurate and efficient TNBC targeting, outstanding tumor microenvironment response, solid tumor deep penetration and lysosomal escape, was designed and synthesized *via* chemical means for the first time. Subsequently, by equipping the ammunitions on the Bio_3_-PEG-BIm decorated nano-rocket (Bio_3_-BIm-NR), novel intelligent multifunctional tumor-targeting nano-missiles (GOx+L-Arg-NM/PTX-NM) were successfully fabricated. Each unit of the nano-missiles was fully used and produced a series of positive chain effects to maximize the delivery of ammunitions to the core area of TNBC. Once internalized by TNBC cells, nano-missiles could automatically trigger cascade catalysis, destroy the homeostasis of TEM and produce strong explosive damage to the TNBC, ultimately eliminating tumor and restraining tumor metastasis. Even better, all components of nano-missiles, which could produce efficient “super-additive” multi-modal synergistic tumor treatment with minimized side effects, were biocompatible and biodegradable. To sum up, the well-designed multifunctional cascade nano-missiles (GOx+L-Arg-NM/PTX-NM) may emerge as a new paradigm to suppress the malignant proliferation and metastasis of TNBC, showing great potentials in future clinical applications.

## Materials and Methods

### Materials

Paclitaxel was obtained from National Institute for Food and Drug Control. Soybean phospholipids (SPC) was purchased from Kelong Chemical (Chengdu, China). Cholesterol (Chol) was purchased from Bio Life Science & Technology Co., Ltd (Shanghai, China). D-(+)-Biotin was purchased from shanghai darui fine chemical Co., Ltd (Shanghai, China). GOx was obtained from Sigma-Aldrich (St. Louis, MO, USA). 1,2-dioleoyl-snglycero-3-phosphoethanolamine-N-(carboxyfluorescein) (CFPE) was purchased from Avanti Polar Lipids (USA). 4'-6-Diamidino-2-phenylindole (DAPI) and 3-(4,5-Dimethylthiazol-2-yl)-2,5-diphenyltetrazolium bromide (MTT) were purchased from Beyotime Institute Biotechnology (Haimen, China). 4-chlorobenzenesulfonate salt (DiD) was purchased from Biotium (USA). Annexin V-FITC/PI apoptosis detection kit was obtained from BD Pharmingen^TM^ (USA). Enhanced BCA Protein Assay Kit (Beyotime^®^, China). Hydrogen Peroxide (H_2_O_2_) Assay Kit (Beyotime®, China). Reactive Oxygen Species (ROS) Assay Kit (Beyotime®, China). Calcein-AM/PI Live/Dead Cell Double Dyed Kit (Solarbio®, China). JC-1 dye (Beyotime®, China).

### Preparation and characterization of nano-rockets and nano-missiles

Nano-rockets were prepared by thin film hydration method. Compositions of different nano-rockets were as follows: (1) ligand-free nano-rocket (LF-NR): SPC/cholesterol (molar ratio = 65 : 35); (2) amino group modified nano-rocket (NH_2_-NR): SPC/cholesterol/compound **23** (molar ratio = 65 : 29 : 6); (3) Bio_3_-PEG-BSa modified nano-rocket (Bio_3_-BSa-NR): SPC/cholesterol/Bio_3_-PEG-BSa (molar ratio = 65 : 29 : 6); (4) Bio_3_-PEG-BIm modified nano-rocket (Bio_3_-BIm-NR): SPC/cholesterol/Bio_3_-PEG-BIm (molar ratio = 65 : 29 : 6).

Nano-missiles can be obtained by simultaneously loading drugs during the preparation of nano-rockets. To prepare GOx+L-Arg-NM, the lipid mixture of SPC, cholesterol and Bio_3_-PEG-BIm were dissolved in the mixture solvent chloroform/methanol (v/v = 2 : 1) and then dried under a rotary evaporator to form a complete lipid film layer. After being kept in vacuum over-night, the dried lipid film was hydrated with GOx+L-Arg PBS solution for 0.5 h at 20 °C and then intermittently sonicated by a probe sonicator at 80 W for 90 s in an ice-water bath to obtain the slightly milky solution. The excess GOx and L-Arg were removed by centrifugation and Sephadex G-150 (Solarbio®, China). GOx-NM was obtained without adding L-Arg during the above preparation process. PTX-NM was prepared following the similar procedure used for the preparation of GOx+L-Arg-NM with a slightly change, of which the PTX was added to the solution of compositions in chloroform/methanol prior to the solvent evaporation, and the unencapsulated PTX was removed by centrifugation.

Analogously, CFPE-labelled (CFPE-LF-NR, CFPE-Bio_3_-BSa-NR and CFPE-Bio_3_-BIm-NR) or DiD-loaded nano-rockets (DiD-LF-NR, DiD-Bio_3_-BSa-NR and DiD-Bio_3_-BSa-NR) were prepared by adding an appropriate amount of CFPE or DiD to the corresponding solution before the solvent evaporation. Free PTX was prepared by dissolving PTX in the mixture of ethanol and Cremophor ELP35 (volume ratio = 1 : 1).

The particle size, polydispersity index (PDI) and zeta potential were determined by DLS analysis using a Malvern Zetasizer Nano ZS90 instrument (Malvern Instruments Ltd, UK). The morphology of GOx+L-Arg-NM and PTX-NM were captured under TEM following negative staining with phosphotungstic acid (2%, w/v). The EE of each nano-missile was determined by the same method, as described previously 18. The amount of GOx, L-Arg and PTX were quantified by HPLC (Agilent 1200 series, Palo Alto, CA) or enhanced BCA Protein Assay Kit (Beyotime®, China), respectively. Turbidity variations were monitored in 50% fetal bovine serum (FBS) to evaluate the serum stability of nano-missiles. Briefly, samples were incubated with the same volume of FBS under 37 °C with gentle oscillation at 45 rpm (final material concentration, 3 mg/mL). The transmittance of each mixture was detected at 750 nm using a Varioskan Flash Multimode Reader (Thermo, USA) at different time points 18. Red blood cells (RBCs) collected from BALB/c mice were isolated from the serum by centrifugation, washed, and diluted with PBS. Subsequently, various concentrations of nano-missiles were mixed with 2% RBC suspensions at 37 °C with moderate shaking at 45 rpm. After 2 h, these samples were centrifuged and the supernatants were collected to examine the hemolysis. Erythrocytes treated with 1% Triton X-100 and PBS were taken as positive and negative controls for 100% and 0% hemolysis, respectively.

### Cascade catalysis performance of the GOx+L-Arg-NM

The capacities of destroying the homeostasis of tumor survival microenvironment were determined by various assays from different aspects. Briefly, different samples containing equivalent amount of GOx (2 μg/mL) and L-Arg (10 μg/mL) were mixed with glucose (at a final concentration of 0.1 to 1 mg/mL) solution at 37 °C. At desired time intervals, the release behavior of H_2_O_2_ and NO in solution were investigated separately by H_2_O_2_ assay kit (Beyotime®, China) and Griess reagent kit (Beyotime®, China) following the manufacturer's protocol. In parallel, the pH reduction of solution was recorded using a pH meter. Meanwhile, the efficiency of L-Arg converted to NO in H_2_O_2_ solution was also demonstrated. Subsequently, the intracellular procreant NO and gluconic acid (reflected by pH) concentration during the cascade catalysis were monitored. 4T1 or MDA-MB-231 cells pre-seeded in 6 well plates or confocal dishes were cultured for 24 h at 37 °C. Then, the cells were administrated with PTX-NM, GOx+L-Arg-NM, GOx+L-Arg-NM&PTX-NM for 6 h, and pure DMEM media were added as the control. After that, the cells were collected and the intracellular NO generation were quantified as mentioned above. A fluorescence NO probe, 4-amino-5-methylamino-2′,7′-difluorofluorescein diacetate (DAF-FM diacetate) was employed for the NO detection. Finally, the pH value was calculated obtained by incubating the cells at various time. The experiments were conducted in triplicate.

### Cellular internalization experiment

The cellular internalization and distribution of different nano-rockets were evaluated by confocal laser scanning microscopy and flow cytometry. Firstly, a precise quantitative comparison of internalization patterns was obtained using a flow cytometer (BD FACSCelesta, BD, USA). 4T1 and MDA-MB-231 cells were separately seeded in 12-well plates with an initial cell density of 3 × 10^5^ cells/well, adhered for 24 h, and then treated with fresh media (pH 7.4 or pH 6.5) containing various nano-rockets for 1 h. After exposure for 1 h, cells were rinsed with cold PBS, trypsinized, centrifuged and harvested in PBS prior to assessing their fluorescence intensity. In addition, to track the intracellular delivery of nano-rockets, cells (5.0 × 10^5^ cells/well) were seeded in 6-well plates with glass coverslips. The cells were incubated for 24 h and added with nano-rockets followed by the treatments as aforesaid. Whereafter, the cells were rinsed with serum-free medium and treated with Lyso-Tracker Red (60 nM) for 1.5 h to stain lysosomes, then subsequently washed repeatedly with cold PBS and fixed with 4% paraformaldehyde for 30 min at room temperature. The cell nuclei were counterstained with DAPI (5 µg/mL) for 5 min. Finally, CLSM (LSM800, Carl Zeiss AG, Germany) was utilized for further imaging.

### Solid tumor penetration

The three-dimensional tumor spheroids employed to evaluate the deep penetrating ability of the nano-rockets were prepared as our previous studies 18. Briefly, 4T1 or MDA-MB-231 cells were plated into 96-well plates (5 × 10^3^ or 1.3 ×10^4^ cells per well) with 2% (w/v) low melting-point agarose at the bottom and incubated under 37 °C, respectively. Spheroids were monitored with optical microscope and cultured to grow up to an appropriate diameter. The uniform and compact spheroids were selected and then treated with different CFPE-labeled nano-rockets at pH 6.5 for 8 h. Afterward, the spheroids were rinsed and fixed, and fluorescent intensity was imaged by CLSM.

### *In vitro* therapeutic efficacy

For the cytotoxicity assay, 4T1 and MDA-MB-231 cells were severally incubated in 96-well plates (6 × 10^3^, 8 × 10^3^ cells per well) for 24 h. Then the culture medium was discarded, and gradient concentrations of PTX-NM, GOx+L-Arg-NM, GOx+L-Arg-NM&PTX-NM were added into each well. Cell viability was quantified by the MTT assay, and the optical density was measured at 490 nm on an automatic microplate spectrophotometer (Thermo Scientific Varioskan Flash, USA). The cytotoxicity was ultimately presented as the percentage of cell viability in contrast with that of the untreated control cells. Cell apoptosis induced by various treatments was detected by an Annexin V-FITC/PI apoptosis detection kit (BD Pharmingen™, USA). Briefly, 4T1 or MDA-MB-231 cells were mixed with various nano-missiles (the concentration of PTX, GOx or L-Arg was 0.5 μg/mL, 0.5 μg/mL or 2.5 μg/mL, respectively) and cultured for 24 h at 37 °C. After staining cells with Annexin V-FITC/PI, apoptosis was quantified by flow cytometry analysis (BD FACSCelesta, BD, USA).

### Wound healing and invasion assays

Wound healing and invasion assays were used to assess the anti-metastasis ability of 4T1 or MDA-MB-231 cells after various treatments. In detail, 4T1 or MDA-MB-231 cells were cultured in 12-well plates and incubated until 95% confluency, respectively. Subsequently, the monolayer of cells was wounded using a 10 μL pipette tip and debris was removed by washing thrice with PBS. Afterwards, different nano-missiles were added and incubated for another 24 h. Images were taken at 0 and 24 h under a microscope and the healed areas in individual group of cells were semi-quantified by Image J 6.0 software. For invasion assay, 4T1 or MDA-MB-231 cells were added to the upper chambers of 24-well Transwell plates (8 µm pore, Corning, USA) coated with Matrigel (BD Biosciences). Then, 100 μL of serum-free medium with PBS, PTX-NM, GOx+L-Arg-NM or GOx+L-Arg-NM&PTX-NM was loaded into each upper chamber, while the lower chambers were filled with 600 μL medium containing 20% FBS as chemoattractant. After 24 h, the cells on the upper surface of the membrane were removed and the invaded cells were fixed in 4% (m/v) paraformaldehyde solution for 30 min and stained with crystal violet, followed by photoimaging. Finally, the crystal violet was dissolved in 33% acetic acid, and the absorbance was measured at 570 nm using a Varioskan Flash multimode reader (ThermoScientific, Varioskan Flash, USA).

### *In vivo* tumor-targeting assay and tumor distribution assay

Female BALB/c mice were anesthetized and subcutaneously injected with 1 × 10^6^ 4T1 cells into the right flank to establish the tumor model. Mice were intravenously administrated with the corresponding DiD-nano-rockets (equivalent to 10 μg of DiD per mouse) through the tail vein. DiD fluorescence images of mice were collected at 1, 2, 4, 6, 8, 12, 24 and 48 h post injection. The mice were sacrificed at 24 h post injection, and the tumors and major organs were excised for *ex vivo* fluorescence imaging. The average fluorescence intensities were calculated by the Image software. Subsequently, tumor tissues were fixed with 4% (w/v) paraformaldehyde, cut into 4 μm sections for CD34 antibody staining and assessed using confocal laser scanning microscope as previously described.

### Assessment on the tumor hemoglobin oxygen saturation

To evaluate whether NO produced by GOx+L-Arg-NM could ameliorate the hypoxia during GOx oxidation. The intratumoral hemoglobin oxygen saturation (sO_2_) was monitored with a photoacoustic (PA) imaging instrument (iThera Medical, MSOT inVision 128 Scanner, GER). 4T1 tumor bearing mice were divided into 3 groups: (I) negative control group with PBS injection; (II) i.v. injection of GOx-NM; (III) i.v. injection of GOx+L-Arg-NM, the final concentration of the GOx in all sample groups was maintained at 1 mg/kg. The images of PA at the wavelengths of 750 and 850 nm were obtained immediately before and 4 h after intravenous injection to monitor the intratumoral sO2 47. Photoacoustic signal intensity was calculated by the photoacoustic system software, and the variation of sO2 were detected and compared corresponding Deo-Hemo and Oxy-Hemo signal. The intratumoral sO2 recorded prior to administration were set at 100%.

### *In vivo* synergistic antitumor efficiency and preliminary safety evaluation

4T1 tumor bearing BALB/c mice were established as described above. Once the tumor volumes reached 50 mm^3^, mice were randomly divided into nine groups (6 mice per group): (a) negative control group with appropriate PBS injection; (b)* i.v.* injection of free PTX; (c) *i.v.* injection of free GOx+L-Arg; (d) *i.v.* injection of PTX-NM; (e) *i.v.* injection of GOx+L-Arg-NM; (f)* i.v.* injection of GOx+L-Arg-NM&PTX-NM, the doses of PTX or GOx were 3 or 1 mg/kg, respectively; (g) *i.v.* injection of GOx+L-Arg-NM&PTX-NM with the dose of PTX reduced to 1 mg/kg. All the groups received treatments on day 1, 3, 5, 8, 11. The tumor volume (0.5 × length × width^2^) and body weight were recorded every 3 days. On the 12th day, the mice were sacrificed, the tumors and major organs were collected. The excised tumors were weighed, photographed and dissected for H&E, TUNEL and Ki67 staining, respectively. Additionally, the major organs were also stained by H&E for systemic toxicity analysis. The tumor inhibition ratio (TIR) was calculated using the equation TIR (%) = (1 - V_t_/V_c_) ×100, where V_t_ and V_c_ were the average tumor volumes at day 25 in the treatment and PBS groups, respectively.

### Evaluation of the therapeutic effect of nano-missiles on pulmonary metastasis

BALB/c tumor-bearing mice were established using the same method described above. Then, 4T1-Luc cells (3 × 10^5^ cells/mouse) were injected intravenously into tumor-bearing mice to generate the pulmonary metastasis mice models. Mice were randomly divided into 5 groups (n = 6) and treated separately for a total of 5 administrations in the same way as mentioned above, the day after injection of cells. At different time points (day 3, day 7, day 11 and day 15), mice were intraperitoneally injected with D-luciferin potassium salt (15 mg/mL, 200 μL). The fluorescence of pulmonary metastases was detected by bioluminescence imaging using an *in vivo* imaging system (IVIS Lumina Series III, PerkinElmer, USA) 10 min later. Mice were sacrificed on the 15th day, and the excised lungs were collected and subjected to H&E staining.

### Statistics analysis

All of the results were expressed as the Mean ± standard deviation (SD). Statistical differences were evaluated with the Student's t-test and performed by one-way ANOVA for multiple groups. Values with P < 0.05 were considered statistically significant (* indicates P < 0.05, ** indicates P < 0.01, *** indicates P < 0.001, **** indicates P < 0.0001).

## Supplementary Material

Supplementary methods, figures and tables.

## Abbreviations

TNBC: triple-negative breast cancer
TME: tumor microenvironment
OS: overall survival
GOx: glucose oxidase
H_2_O_2_: hydrogen peroxide
NO: nitric oxide
NM: nano-missile
NR: nano-rocket
PEG: polyethylene glycol
Bio: biotin
SMVT: sodium-dependent multivitamin transporter
EPR: enhanced permeability and retention
PTX: paclitaxel
Hz: hydrazone
AIm: alkyl imine
PIm: phenyl imine
BIm: benzyl imine
BSa: benzyl secondary amine
NMR: nuclear magnetic resonance
DLS: dynamic light scattering
TEM: transmission electron microscopy
PBS: phosphate balanced solution
EE: encapsulation efficiency
L-Arg: L-arginine
PA: photoacoustic
MMPs: matrix metalloproteinases
TIR: tumor inhibition ratio
SPC: soybean phospholipids
Chol: cholesterol
ROS: reactive oxygen species
PDI: polydispersity index
FBS: fetal bovine serum
RBCs: red blood cells
SD: standard deviation
