# Supplementary material for: Accurate programmed multifunctional nano-missiles for self-promoted deep delivery and synergistic cascade tumor therapy: Tactfully collaborating chemosynthesis with tumor microenvironment remodeling
Source: Theranostics. 2022 Jul 4;12(12):5299–316. doi: 10.7150/thno.74550 (PMC9330536; doi:10.7150/thno.74550)

Supporting Information

Accurate programmed multifunctional nano-missiles for self-promoted deep delivery and synergistic cascade tumor therapy: Tactfully collaborating chemosynthesis with tumor microenvironment remodeling

Runxin Lu[1,*], Siqi Wang[2], Zhongzhen Yang[2], Lin Zhou[2], Chunyan Yang[2], Yong Wu[2,*]

[1]*Department of Pharmacy, Evidence-Based Pharmacy Center, Key Laboratory of Birth Defects and Related Diseases of Women and Children (Sichuan University), West China Second University Hospital, Sichuan University, Chengdu, China.*

[2]*Key Laboratory of Drug-Targeting and Drug Delivery System of the Education Ministry and Sichuan Province, Sichuan Engineering Laboratory for Plant-Sourced Drug and Sichuan Research Center for Drug Precision Industrial Technology, West China School of Pharmacy, Sichuan University, Chengdu, China.*

*Corresponding authors. lurunxin@scu.edu.cn (Runxin Lu), wyong@scu.edu.cn (Yong Wu).

1. The synthesis of the ligands Bio$_3$-PEG-BIm, Bio$_3$-PEG-AIm, Bio$_3$-PEG-PIm, Bio$_3$-PEG-Hz and Bio$_3$-PEG-BSa

1.1 Chemicals and materials

All reactions were carried out under usual atmosphere unless otherwise stated. All liquid reagents were distilled before use. All unspecified reagents were from commercial resources. TLC was performed using precoated silica gel GF254 (0.2 mm), while column chromatography was performed using silica gel (100-200 mesh). ^{1}H NMR spectra was taken on a Varian INOVA 400 (Varian, Palo Alto, CA, USA) using $CDCl_3$ or DMSO-d_6 as solvents. Chemical shifts are expressed in δ (ppm), with tetramethylsilane (TMS) functioning as the internal reference, and coupling constants (J) were expressed in Hz.

1.2 Synthesis of ligand Bio$_3$-PEG-BIm

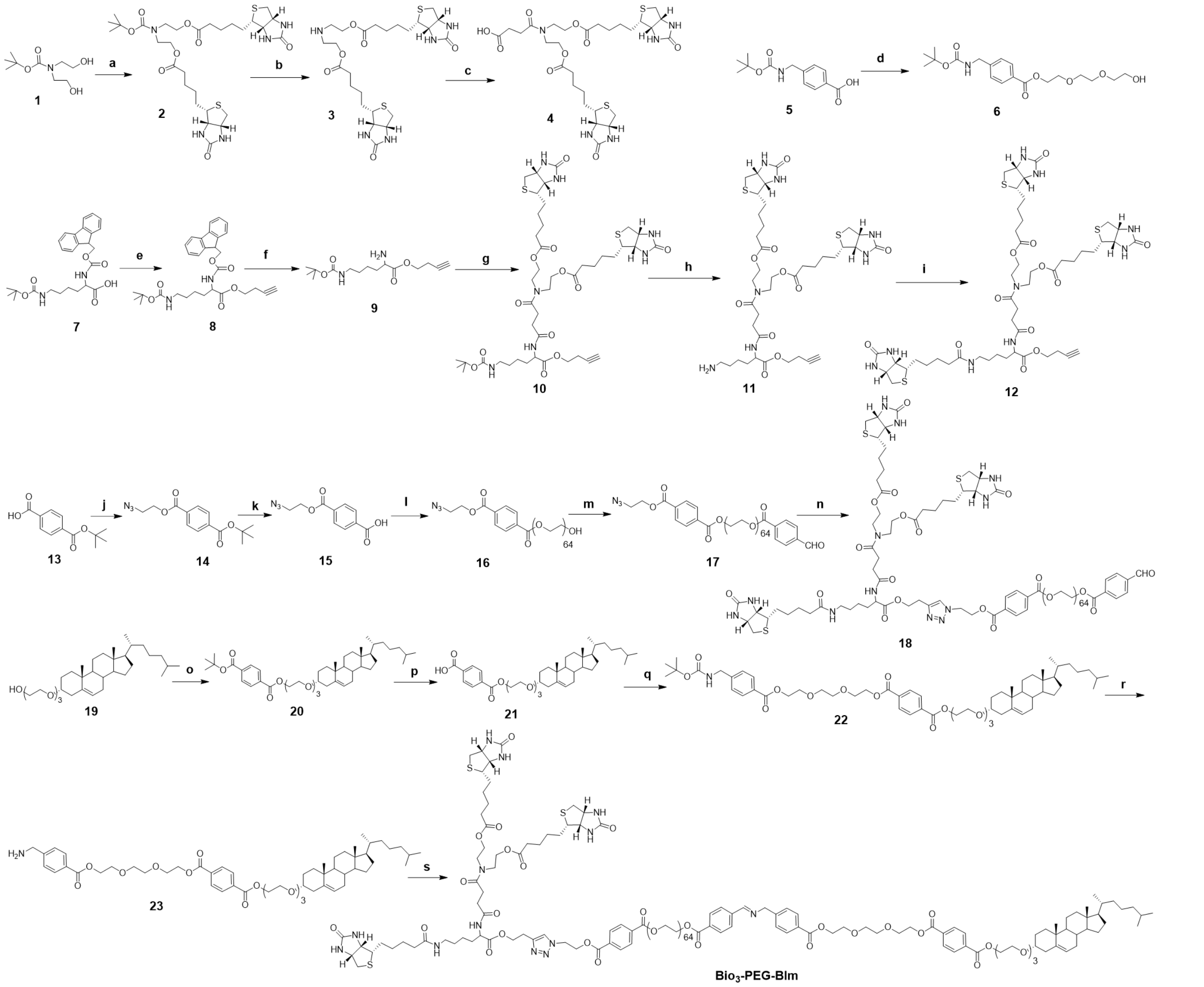

Scheme S1. The synthetic route of the ligand Bio$_3$-PEG-BIm. Reagents and conditions: (a) Biotin, EDCI, DMAP, DIPEA, DCM, DMF, r.t., 18h, 74.1%; (b) CF_3COOH, DCM, r.t., 3 h, 81.2%; (c) Succinic anhydride, Et_3N, DCM, r.t., 6 h, 77.1%; (d) Triethylene glycol, EDCI, DMAP, DIPEA, DCM, r.t., 5 h, 76.3%; (e) 3-Butyn-1-ol, DCC, DMAP, DCM, r.t., 2 h, 84.6%; (f) DBU, DCM, r.t., 30 min, 58.1%; (g) Compound **4**, HATU, DIPEA, DCM/DMF, r.t., 2 h, 84.7%; (h) CF_3COOH, DCM, r.t., 2 h, 70.3%; (i) Biotin, HATU, DIPEA, DCM/DMF, r.t., 2 h, 70.3%; (j)

2-Azidoethanol, EDCI, DMAP, DIPEA, DCM, r.t., 12 h, 83.5%; (k) CF_3COOH, Et_3SiH, DCM, r.t., 3 h, 71.9%; (l) PEG_{3350}, EDCI, DMAP, DIPEA, DCM, r.t., 16 h, 90.1%; (m) 4-Formylbenzoic acid, DCC, DMAP, DCM, r.t., 24 h, 75.2%; (n) Compound **12**, sodium ascorbate, CuI, DMF/H_2O, r.t., 16 h, 55.2%; (o) 4-(tert-Butoxycarbonyl)benzoic acid, EDCI, DMAP, DIPEA, DCM, r.t., 5 h, 84.7%; (p) CF_3COOH, Et_3SiH, DCM, r.t., 2 h, 76.3%; (q) Compound **6**, DCC, DMAP, DCM, r.t., 2 h, 70.3%; (r) CF_3COOH, DCM, r.t., 2 h, 76.2%; (s) Compound **18**, AcOH, molecular sieves, DMSO, 40°C, 4 h, 77.4%.

Ligand Bio_3-PEG-BIm was prepared using the methodologies depicted in Scheme S1. In the initial step, the esterification reaction of compound **1** with excess biotin resulted in the intermediate **2**, which was then subjected to the Boc-deprotection reaction and condensation reaction to yield intermediate **4**. Both intermediates **6** and **8** could be successfully obtained *via* esterification reaction using commercially sourced raw materials. Fmoc-deprotection with DBU in dichloromethane afforded the primary amine **9**, which was followed by amidation with compound **4**, Boc-deprotection with TFA and amidation with biotin to produce the intermediate alkyne **12**. By reacting with commercially available 2-azidoethanol, compound **13** was converted to intermediate **14**. After removing the tert-butyl group, it was successively esterified with PEG_{3350} and 4-formylbenzoic acid to yield intermediate **17**. Subsequently, the click reaction of compound **17** with alkyne **12** in the presence of CuI generated the key intermediate **18**. Similarly, another key intermediate, benzylamine **23**, could be easily prepared in four steps starting from the known compound **19**. Finally, benzaldehyde **18** reacted with benzylamine **23** to reach the target compound (Bio_3-PEG-BIm) by using anhydrous acetic acid as the catalyst.

1.2.1 Synthesis of compound **2**.

To a solution of biotin (2.38 g, 9.76 mmol) in dichloromethane (100 mL) and DMF (100 mL) was added EDCI (2.81 g, 14.64 mmol), DMAP (1.79 g, 14.64 mmol) and DIPEA (2.84 g, 21.96 mmol), and the reaction was stirred at -5 °C for 30 min. Then, compound **1** (500 mg, 2.44 mmol) in dichloromethane (10 mL) was added slowly. After stirring for another 18h at room temperature, the mixture was washed with 1 mol/L HCl (100 mL x 2) and extracted three times with dichloromethane. The combined organic layers were washed with water and brine, dried over anhydrous sodium sulfate, filtered, concentrated and purified by silica gel column chromatography to afford compound **2** as a white solid (1.19 g, 74.1%). M.p. > 200°C. ^{1}H-NMR (600 MHz, $CDCl_3$, ppm) δ 6.56 (s, 1H), 6.48 (s, 1H), 5.86 (brs, 1H), 5.77 (brs, 1H), 4.50 (t, J = 6.0 Hz, 2H), 4.31-4.33 (m, 2H), 4.17-4.18 (m, 4H), 3.50 (s, 2H), 3.42-3.48 (m, 2H), 3.13-3.15 (m, 2H), 2.90 (dd, J_1 = 12.6 Hz, J_2 = 4.2 Hz, 2H), 2.73 (d, J = 12.6 Hz, 2H), 2.36 (s, 4H), 1.63-1.74 (m, 8H), 1.39-1.45 (m, 13H). ^{13}C-NMR (150 MHz, $CDCl_3$, ppm) δ 173.7, 164.1, 155.2, 80.4, 62.6, 62.4, 61.9, 61.8, 60.2, 55.4, 47.5, 47.4, 40.7, 33.9, 28.4, 28.2, 28.1, 25.0, 24.9. LR-MS: (ESI) calcd. for $C_{29}H_{48}N_5O_8S_2$ $[M+H]^+$ 658.3, found 658.2.

1.2.2 Synthesis of compound **3**.

Trifluoroacetic acid (2.5 mL, 10% v/v) was added to the solution of compound **2** (1.3 g) in dichloromethane (25 mL). After stirred at room temperature for 3h, the mixture was adjusted to pH 9 with saturated sodium bicarbonate

solution and extracted three times with dichloromethane. The combined organic layers were washed with water and brine, dried over anhydrous sodium sulfate, filtered, concentrated and purified by silica gel column chromatography to afford compound **3** as a white solid (896.6 mg, 81.2%). M.p. > 200ºC. ^{1}H-NMR (400 MHz, DMSO-d_6, ppm) δ 8.81 (brs, 1H), 6.41 (s, 2H), 6.37 (s, 2H), 4.30-4.33 (m, 2H), 4.27 (t, J = 4.8 Hz, 4H), 4.12-4.15 (m, 2H), 3.28 (s, 4H), 3.08-3.13 (m, 2H), 2.82 (dd, J_1 = 12.4 Hz, J_2 = 5.2 Hz, 2H), 2.58 (d, J = 12.4 Hz, 2H), 2.36 (t, J = 7.2 Hz, 4H), 1.43-1.67 (m, 8H), 1.32-1.41 (m, 4H). ^{13}C-NMR (100 MHz, $CDCl_3$, ppm) δ 173.7, 164.0, 63.3, 61.9, 60.2, 55.5, 47.8, 40.6, 33.8, 28.4, 28.2, 24.9.

1.2.3 Synthesis of compound **4**.

The solution of succinic anhydride (63 mg, 0.62 mmol) in dichloromethane (3 mL) was stirred at 0ºC for 5min, then a mixture of compound **3** (210 mg, 0.31 mmol) and trethylamine (174 μL, 1.24 mmol) in dichloromethane (5 mL) was added dropwise. After stirring for another 6h at room temperature, the reaction mixture was concentrated and purified by silica gel column chromatography to afford compound **4** as a white solid (157.2 mg, 77.1%). M.p. 194.6-196.2ºC. ^{1}H-NMR (400 MHz, DMSO-d_6, ppm) δ 6.47 (s, 1H), 6.45 (s, 1H), 6.41 (s, 1H), 6.38 (s, 1H), 4.31 (s, 2H), 4.14-4.18 (m, 4H), 4.07 (s, 2H), 3.61 (s, 2H), 3.50 (s, 2H), 3.10 (s, 2H), 2.83 (dd, J_1 = 12.4 Hz, J_2 = 4.8 Hz, 2H), 2.56-2.63 (m, 4H), 2.42 (s, 2H), 2.26-2.35 (m, 4H), 1.42-1.60 (m, 8H), 1.31-1.33 (m, 4H). LR-MS: (ESI) calcd. for $C_{28}H_{44}N_5O_9S_2$ $[M + H]^+$ 658.3, found 658.2.

1.2.4 Synthesis of compound **6**.

To a solution of compound **5** (500 mg, 1.99 mmol) in dichloromethane (8 mL) was added EDCI (573 mg, 2.98 mmol), DMAP (365 mg, 2.98 mmol) and DIPEA (515 mg, 3.98 mmol), and the reaction was stirred at -5 ºC for 30 min. Then, triethylene glycol (747 mg, 4.97 mmol) in dichloromethane (1 mL) was added slowly. After stirring for another 5h at room temperature, the mixture was washed with 1 mol/L HCl and extracted three times with dichloromethane. The combined organic layers were washed with water and brine, dried over anhydrous sodium sulfate, filtered, concentrated and purified by silica gel column chromatography to afford compound **2** as a yellowish oily (582.1 mg, 76.3%). ^{1}H-NMR (400 MHz, DMSO-d_6, ppm) δ 7.92 (d, J = 8.0 Hz, 2H), 7.48 (t, J = 6.0 Hz, 1H), 7.37 (d, J = 8.0 Hz, 2H), 4.57 (t, J = 5.2 Hz, 1H), 4.37 (t, J = 4.8 Hz, 2H), 4.19 (d, J = 6.0 Hz, 2H), 3.74 (t, J = 4.8 Hz, 2H), 3.58-3.60 (m, 2H), 3.52-3.54 (m, 2H), 3.45-3.49 (m, 2H), 3.41 (t, J = 4.8 Hz, 2H), 1.39 (s, 9H).

1.2.5 Synthesis of compound **8**.

To a solution of compound **7** (10 g, 21.3 mmol) in dichloromethane (80 mL) was added DCC (8.8 g, 42.6 mmol) and DMAP (522 mg, 4.26 mmol), and the reaction was stirred at -5 ºC for 30 min. Then, 3-Butyn-1-ol (2.24 g, 32 mmol) in dichloromethane (5 mL) was added slowly. After stirring for another 2h at room temperature, the mixture was cooled at -20 ºC for 5h and filtered through a Buchner funnel. The filtrate was washed with 1 mol/L HCl and extracted three times with dichloromethane. The combined organic layers were washed with water and brine, dried over anhydrous

sodium sulfate, filtered, concentrated and purified by silica gel column chromatography to afford compound **8** as a yellowish oily (9.38 g, 84.6%). ^{1}H-NMR (400 MHz, $CDCl_3$, ppm) δ 7.77 (d, J = 7.6 Hz, 2H), 7.61 (d, J = 7.6 Hz, 2H), 7.40 (t, J = 7.6 Hz, 2H), 7.32 (t, J = 7.6 Hz, 2H), 5.41 (brs, 1H), 4.57 (brs, 1H), 4.18-4.45 (m, 6H), 3.12 (d, J = 6.0 Hz, 2H), 2.58 (td, J_1 = 6.4 Hz, J_2 = 2.8 Hz, 2H), 2.02 (t, J = 2.8 Hz, 1H), 1.84-1.93 (m, 1H), 1.65-1.78 (m, 3H), 1.36-1.51 (m, 11H). ^{13}C-NMR (100 MHz, $CDCl_3$, ppm) δ 172.2, 156.1, 155.9, 143.9, 143.7, 141.3, 127.7, 127.1, 125.1, 120.0, 79.6, 79.2, 70.3, 67.0, 63.0, 53.7, 47.2, 40.1, 32.2, 29.6, 28.4, 22.3, 18.9. LR-MS: (ESI) calcd. for $C_{25}H_{29}N_2O_4$ $[M\text{-}Boc+H]^+$ 421.2, found 421.1.

1.2.6 Synthesis of compound **9**.

DBU (6.37 g, 41.9 mmol) was added to the solution of compound **8** (7.27 g， 13.96 mmol) in dichloromethane (60 mL). After stirred at room temperature for 30 min, the mixture was washed with water and brine. Then, the combined organic layers were dried over anhydrous sodium sulfate, filtered, concentrated and purified by silica gel column chromatography to afford compound **9** as a colorless transparent oil (2.42 g, 58.1%). ^{1}H-NMR (400 MHz, DMSO-d_6, ppm) δ 6.75 (brs, 1H), 4.03-4.16 (m, 2H), 3.26-3.29 (m, 1H), 2.85-2.90 (m, 3H), 2.49-2.53 (m, 2H), 1.72 (brs, 2H), 1.51-1.60 (m, 1H), 1.42-1.47 (m, 1H), 1.24-1.37 (m, 13H). ^{13}C-NMR (100 MHz, $CDCl_3$, ppm) δ 175.7, 156.0, 79.9, 79.1, 70.1, 62.5, 54.3, 40.4, 34.4, 29.8, 28.4, 22.8, 19.0. LR-MS: (ESI) calcd. for $C_{15}H_{27}N_2O_4$ $[M+H]^+$ 299.2, found 299.2.

1.2.7 Synthesis of compound **10**.

To a solution of compound **4** (3.98 g, 6.05 mmol) in dichloromethane (60 mL) and DMF (6 mL) was added HATU (2.63 g, 6.91 mmol) and DIPEA (1.49 g, 11.52 mmol), and the reaction was stirred at -5 °C for 30 min. Then, compound **9** (1.72 g, 5.76 mmol) in dichloromethane (5 mL) was added slowly. After stirring for another 2h at room temperature, the mixture was washed with 1 mol/L HCl and extracted three times with dichloromethane. The combined organic layers were washed with water and brine, dried over anhydrous sodium sulfate, filtered, concentrated and purified by silica gel column chromatography to afford compound **10** as a yellowish oily (4.58 g, 84.7%). ^{1}H-NMR (400 MHz, DMSO-d_6, ppm) δ 8.19 (d, J = 7.2 Hz, 1H), 6.74 (brs, 1H), 6.40 (s, 2H), 6.35 (s, 2H), 4.29-4.32 (m, 2H), 4.11-4.17 (m, 6H), 4.03-4.08 (m, 2H), 3.60 (t, J = 4.8 Hz, 2H), 3.48-3.51 (m, 2H), 3.38 (q, J = 6.8 Hz, 1H), 3.07-3.12 (m, 2H), 2.80-2.90 (m, 5H), 2.54-2.59 (m, 6H), 2.36-2.43 (m, 2H), 2.26-2.34 (m, 4H), 1.43-1.68 (m, 10H), 1.37 (s, 9H), 1.24-1.33 (m, 8H). LR-MS: (ESI) calcd. for $C_{43}H_{68}N_7O_{12}S_2$ $[M+H]^+$ 938.4, found 938.3.

1.2.8 Synthesis of compound **11**.

Trifluoroacetic acid (7.5 mL, 30% V/V) was added to the solution of compound **10** (5.7 g, 6.08mmol) in dichloromethane (25 mL). After stirred at room temperature for 2h, the mixture was adjusted to pH 9 with saturated sodium bicarbonate solution and extracted three times with dichloromethane. The combined organic layers were washed with water and brine, dried over anhydrous sodium sulfate, filtered, concentrated and purified by silica gel column

chromatography to afford compound **11** as a yellowish oily (3.58 g, 70.3%). ^{1}H-NMR (600 MHz, DMSO-d_6, ppm) δ 8.22 (brs, 1H), 7.71 (brs, 1H), 7.67 (brs, 1H), 6.42 (s, 2H), 6.37 (s, 2H), 4.30-4.31 (m, 2H), 4.14-4.23 (m, 6H), 4.05-4.07 (m, 2H), 3.60 (s, 2H), 3.49-3.50 (m, 3H), 3.09 (s, 2H), 2.87 (s, 1H), 2.82 (dd, J_1 = 12.0 Hz, J_2 = 4.8 Hz, 2H), 2.68 (t, J = 6.0 Hz, 2H), 2.55-2.61 (m, 4H), 2.37-2.43 (m, 2H), 2.27-2.33 (m, 4H), 1.97-2.02 (m, 2H), 1.59-1.65 (m, 2H), 1.52-1.53 (m, 2H), 1.45-1.46 (m, 2H), 1.28-1.38 (m, 6H), 1.23 (s, 6H). LR-MS: (ESI) calcd. for $C_{38}H_{60}N_7O_{10}S_2$ $[M+H]^+$ 838.4, found 838.4.

1.2.9 Synthesis of compound **12**.

To a solution of biotin (268 mg, 1.09 mmol) in dichloromethane (10 mL) and DMF (10 mL) was added HATU (501 mg, 1.32 mmol) and DIPEA (284 mg, 2.19 mmol), and the reaction was stirred at -5 ^{o}C for 30 min. Then, compound **11** (613 mg, 0.73 mmol) in dichloromethane (5 mL) was added slowly. After stirring for another 2h at room temperature, the mixture was washed with 1 mol/L HCl and extracted three times with dichloromethane. The combined organic layers were washed with water and brine, dried over anhydrous sodium sulfate, filtered, concentrated and purified by silica gel column chromatography to afford compound **12** as a yellowish oily (546.2 mg, 70.3%). ^{1}H-NMR (400 MHz, DMSO-d_6, ppm) δ 8.22 (d, J = 7.2 Hz, 1H), 7.75 (t, J = 5.6 Hz, 1H), 6.44 (s, 3H), 6.38 (s, 3H), 4.29-4.32 (m, 3H), 4.10-4.19 (m, 7H), 4.02-4.09 (m, 3H), 3.59-3.61 (m, 2H), 3.50 (t, J = 5.6 Hz, 2H), 3.17 (d, J = 5.2 Hz, 3H), 3.09-3.10 (m, 3H), 2.98-3.02 (m, 2H), 2.87 (t, J = 2.8 Hz, 1H), 2.82 (dd, J_1 = 12.4 Hz, J_2 = 4.8 Hz, 3H), 2.54-2.62 (m, 4H), 2.47-2.48 (m, 2H), 2.34-2.45 (m, 2H), 2.26-2.34 (m, 4H), 2.04 (t, J = 7.2 Hz, 2H), 1.41-1.69 (m, 14H), 1.30-1.37 (m, 8H). LR-MS: (ESI) calcd. for $C_{48}H_{74}N_9O_{12}S_3$ $[M+H]^+$ 1064.5, found 1064.5.

1.2.10 Synthesis of compound **14**.

To a solution of compound **13** (1.0 g, 4.50 mmol) in dichloromethane (36 mL) was added EDCI (1.3 g, 6.75 mmol), DMAP (830 mg, 6.75 mmol) and DIPEA (1.16 g, 9.0 mmol), and the reaction was stirred at -5 ^{o}C for 30 min. Then, 2-azidoethanol (587 mg, 6.75 mmol) in dichloromethane (3 mL) was added slowly. After stirring for another 12h at room temperature, the mixture was washed with 1 mol/L HCl and extracted three times with dichloromethane. The combined organic layers were washed with water and brine, dried over anhydrous sodium sulfate, filtered, concentrated and purified by silica gel column chromatography to afford compound **14** as a white solid (1.09 g, 83.5%). M.p. > 200°C. ^{1}H-NMR (400 MHz, DMSO-d_6, ppm) δ 8.08 (d, J = 8.4 Hz, 2H), 8.03 (d, J = 8.4 Hz, 2H), 4.48 (t, J = 4.8 Hz, 2H), 3.69 (t, J = 4.8 Hz, 2H), 1.55 (s, 9H).

1.2.11 Synthesis of compound **15**.

The solution of compound **14** (1.2 g, 4.12 mmol) in dichloromethane (2 mL) was stirred at 0°C for 5min, Then, triethylsilane (1.2 g, 10.3 mmol) in dichloromethane (1.5 mL) and trifluoroacetic acid (8 mL) was added slowly. After stirring for another 3h at room temperature, the mixture was concentrated and purified by recrystallization to afford

compound **15** as a white solid (696.7 mg, 71.9%). M.p. > 200°C. ^{1}H-NMR (400 MHz, DMSO-d_6, ppm) δ 8.08 (s, 4H), 4.48 (t, J = 4.8 Hz, 2H), 3.70 (t, J = 4.8 Hz, 2H). ^{13}C-NMR (100 MHz, DMSO-d_6, ppm) δ 167.3, 165.7, 135.8, 133.6, 130.4, 130.2, 64.9, 50.1.

1.2.12 Synthesis of compound **16**.

To a solution of compound **15** (500 mg, 2.13 mmol) in dichloromethane (30 mL) was added EDCI (612 mg, 3.20 mmol), DMAP (390 mg, 3.20 mmol) and DIPEA (550 mg, 4.26 mmol), and the reaction was stirred at -5 °C for 30 min. Then, PEG_{3350} (7.13 g, 2.13 mmol) in dichloromethane (5 mL) was added slowly. After stirring for another 16h at room temperature, the mixture was washed with 1 mol/L HCl and extracted three times with dichloromethane. The combined organic layers were washed with water and brine, dried over anhydrous sodium sulfate, filtered, concentrated and purified by recrystallization (DCM/Et_2O) to afford compound **16** as a white solid (5.85 g, 90.1%). M.p. 72.6-74.2°C. ^{1}H-NMR (400 MHz, DMSO-d_6, ppm) δ 8.11 (s, 4H), 4.56 (t, J = 5.2 Hz, 2H), 4.49 (t, J = 4.8 Hz, 2H), 4.43 (t, J = 4.8 Hz, 2H), 3.76 (t, J = 4.8 Hz, 2H), 3.67-3.72 (m, 4H), 3.40-3.59 (m, 248H).

1.2.13 Synthesis of compound **17**.

To a solution of 4-formylbenzoic acid (407 mg, 2.7 mmol) in dichloromethane (20 mL) was added DCC (741.6 mg, 3.6 mmol) and DMAP (44.0 mg, 0.36 mmol), and the reaction was stirred at -5 °C for 30 min. Then, compound **16** (6.45 g, 1.8 mmol) in dichloromethane (5 mL) was added slowly. After stirring for another 24h at room temperature, the mixture was cooled at -20 °C for 5h and filtered through a Buchner funnel. The filtrate was washed with 1 mol/L HCl and extracted three times with dichloromethane. The combined organic layers were washed with water and brine, dried over anhydrous sodium sulfate, filtered, concentrated and purified by recrystallization (DCM/Et_2O) to afford compound **17** as a white solid (4.3 g, 75.2%). M.p. 88.8-90.2°C. ^{1}H-NMR (400 MHz, DMSO-d_6, ppm) δ 10.12 (s, 1H), 8.16 (d, J = 8.4 Hz, 2H), 8.11 (s, 4H), 8.06 (d, J = 8.4 Hz, 2H), 4.42-4.43 (m, 4H), 3.77 (s, 4H), 3.67-3.72 (m, 4H), 3.59-3.60 (m, 6H), 3.51 (s, 242H).

1.2.14 Synthesis of compound **18**.

To a solution of compound **12** (100 mg, 0.094 mmol) and compound **17** (448.8 mg, 0.141 mmol) in the mixed solvent of DMF (3 mL) and water (0.3 mL) was added CuI (5.3 mg, 0.028 mmol) and sodium ascorbate (11.1 mg, 0.056 mmol). After stirring for another 16h at room temperature under the protection of argon, the mixture was concentrated and purified by silica gel column chromatography to afford compound **18** as a yellowish oily (220.4 mg, 55.2%). ^{1}H-NMR (600 MHz, DMSO-d_6, ppm) δ 10.11 (s, 1H), 8.21 (d, J = 6.6 Hz, 1H), 8.15-8.18 (m, 2H), 8.08 (s, 1H), 8.05 (d, J = 8.4 Hz, 2H), 8.01 (d, J = 8.4 Hz, 2H), 7.73-7.75 (m, 3H), 6.42 (s, 3H), 6.36 (s, 3H), 4.76-4.77 (m, 2H), 4.67-4.68 (m, 2H), 4.41-4.45 (m, 3H), 4.21-4.36 (m, 6H), 4.12-4.16 (m, 6H), 4.03-4.06 (m, 2H), 3.75-3.76 (m, 2H), 3.58 (s, 4H), 3.47-3.50 (m, 250H), 3.08 (s, 3H), 2.94-3.00 (m, 5H), 2.80-2.85 (m, 3H), 2.58 (s, 2H), 2.56 (s, 2H), 2.38-2.44 (m, 2H),

2.25-2.37 (m, 6H), 2.02-2.04 (m, 2H), 1.43-1.59 (m, 14H), 1.22-1.30 (m, 8H).

1.2.15 Synthesis of compound **20**.

To a solution of compound **13** (1.0 g, 4.50 mmol) in dichloromethane (36 mL) was added EDCI (1.3 g, 6.75 mmol), DMAP (830 mg, 6.75 mmol) and DIPEA (1.16 g, 9.0 mmol), and the reaction was stirred at -5 ^{o}C for 30 min. Then, compound **19** (1.56 g, 3.0 mmol) in dichloromethane (3 mL) was added slowly. After stirring for another 5h at room temperature, the mixture was washed with 1 mol/L HCl and extracted three times with dichloromethane. The combined organic layers were washed with water and brine, dried over anhydrous sodium sulfate, filtered, concentrated and purified by silica gel column chromatography to afford compound **20** as a yellowish oily (1.84 g, 84.7%). ^{1}H-NMR (400 MHz, $CDCl_3$, ppm) δ 8.09 (d, *J* = 8.4 Hz, 2H), 8.03 (d, *J* = 8.4 Hz, 2H), 5.32-5.33 (m, 1H), 4.49 (t, *J* = 4.8 Hz, 2H), 3.85 (t, *J* = 4.8 Hz, 2H), 3.70 (d, *J* = 4.8 Hz, 4H), 3.63 (s, 4H), 3.13-3.20 (m, 1H), 1.00-2.37 (28H, remaining cholesterol protons), 1.61 (s, 9H), 0.98 (s, 3H), 0.91 (d, *J* = 6.4 Hz, 3H), 0.86 (d, *J* = 6.8 Hz, 6H), 0.67 (s, 3H).

1.2.16 Synthesis of compound **21**.

The solution of compound **20** (1.15 g, 1.59 mmol) in dichloromethane (2 mL) was stirred at 0°C for 5min, Then, triethylsilane (462.4 mg, 3.98 mmol) in dichloromethane (1 mL) and trifluoroacetic acid (7 mL) was added slowly. After stirring for another 2h at room temperature, the mixture was concentrated and purified by silica gel column chromatography to afford compound **21** as a colorless transparent oil (808.9 mg, 76.3%). ^{1}H-NMR (400 MHz, $CDCl_3$, ppm) δ 8.13 (s, 4H), 5.31-5.33 (m, 1H), 4.51 (t, *J* = 4.8 Hz, 2H), 3.86 (t, *J* = 4.8 Hz, 2H), 3.71 (d, *J* = 4.8 Hz, 4H), 3.63 (s, 4H), 3.13-3.20 (m, 1H), 1.00-2.37 (28H, remaining cholesterol protons), 0.98 (s, 3H), 0.91 (d, *J* = 6.4 Hz, 3H), 0.86 (d, *J* = 6.8 Hz, 6H), 0.67 (s, 3H).

1.2.17 Synthesis of compound **22**.

To a solution of compound **21** (250 mg, 0.36 mmol) in dichloromethane (3 mL) was added DCC (155 mg, 0.72 mmol) and DMAP (9.5 mg, 0.07 mmol), and the reaction was stirred at -5 ^{o}C for 20 min. Then, compound **6** (216 mg, 0.54 mmol) in dichloromethane (0.5 mL) was added slowly. After stirring for another 2h at room temperature, the mixture was washed with 1 mol/L HCl and extracted three times with dichloromethane. The combined organic layers were washed with water and brine, dried over anhydrous sodium sulfate, filtered, concentrated and purified by silica gel column chromatography to afford compound **22** as a yellowish oily (261.3 mg, 70.3%). ^{1}H-NMR (400 MHz, DMSO-d_6, ppm) δ 8.07 (s, 4H), 7.89 (d, *J* = 8.0 Hz, 2H), 7.46 (t, *J* = 5.6 Hz, 1H), 7.35 (d, *J* = 8.0 Hz, 2H), 5.25 (s, 1H), 4.40 (t, *J* = 8.8 Hz, 4H), 4.31 (t, *J* = 4.4 Hz, 2H), 4.18 (d, *J* = 5.6 Hz, 2H), 3.73-3.76 (m, 6H), 3.58-3.61 (m, 6H), 3.52-3.54 (m, 2H), 3.47 (s, 4H), 3.03-3.09 (m, 1H), 0.93-2.28 (28H, remaining cholesterol protons), 1.39 (s, 9H), 0.89 (s, 3H), 0.87 (s, 3H), 0.84 (d, *J* = 6.4 Hz, 6H), 0.62 (s, 3H).

1.2.18 Synthesis of compound **23**.

Trifluoroacetic acid (1.5 mL, 30% V/V) was added to the solution of compound **22** (200 mg, 0.194 mmol) in dichloromethane (5 mL). After stirred at room temperature for 2h, the mixture was adjusted to pH 9 with saturated sodium bicarbonate solution and extracted three times with dichloromethane. The combined organic layers were washed with water and brine, dried over anhydrous sodium sulfate, filtered, concentrated and purified by silica gel column chromatography to afford compound **23** as a yellowish oily (137.8 mg, 76.2%). ^{1}H-NMR (400 MHz, DMSO-d_6, ppm) δ 8.07 (s, 4H), 7.87 (d, J = 8.0 Hz, 2H), 7.45 (d, J = 8.0 Hz, 2H), 5.24(s, 1H), 4.40 (t, J = 8.8 Hz, 4H), 4.34 (t, J = 4.4 Hz, 2H), 3.78 (s, 2H), 3.73-3.77 (m, 6H), 3.58-3.61 (m, 6H), 3.51-3.54 (m, 2H), 3.47 (s, 4H), 3.23 (brs, 2H), 3.03-3.09 (m, 1H), 0.93-2.27 (28H, remaining cholesterol protons), 0.89 (s, 3H), 0.87 (s, 3H), 0.84 (d, J = 6.4 Hz, 6H), 0.62 (s, 3H).

1.2.19 Synthesis of ligand Bio$_3$-PEG-BIm.

Compound **18** (42.5 mg, 0.01 mmol) and Compound **23** (14.0 mg, 0.015 mmol) were dissolved in anhydrous DMSO (1 mL). Then, molecular sieves (20 mg) and anhydrous AcOH (2 drops) were added under the protection of argon. After stirred at 40°C for 4h, the mixture was diluted with saturated sodium bicarbonate solution, filtered, and extracted three times with dichloromethane. The combined organic layers were washed with water and brine, dried over anhydrous sodium sulfate, filtered, concentrated and purified by dialysis and lyophilization to afford Bio$_3$-PEG-BIm as a white solid (40.0 mg, 77.4%). ^{1}H-NMR (400 MHz, DMSO-d_6, ppm) δ 8.62 (s, 1H), 8.16 (d, J = 7.2 Hz, 1H), 8.07-8.09 (m, 5H), 8.04 (d, J = 8.4 Hz, 2H), 8.02 (d, J = 8.4 Hz, 2H), 7.93 (d, J = 8.0 Hz, 2H), 7.88 (d, J = 8.0 Hz, 2H), 7.69-7.71 (m, 2H), 7.48 (d, J = 8.4 Hz, 2H), 7.37 (t, J = 5.6 Hz, 1H), 6.40 (s, 3H), 6.34 (s, 3H), 5.24 (s, 1H), 4.77 (d, J = 4.4 Hz, 2H), 4.68 (t, J = 4.4 Hz, 2H), 4.35-4.42 (m, 7H), 4.24-4.32 (m, 8H), 4.12-4.16 (m, 6H), 4.06 (t, J = 4.8 Hz, 2H), 3.76 (s, 10H), 3.68 (t, J = 4.8 Hz, 2H), 3.58-3.61 (m, 12H), 3.40-3.51 (m, 252H), 3.09 (s, 4H), 2.94-2.98 (m, 5H), 2.79-2.83 (m, 3H), 2.54-2.59 (m, 4H), 2.37-2.42 (m, 2H), 2.25-2.32 (m, 7H), 2.04 (t, J = 7.2 Hz, 3H), 1.03-1.97 (48H, including 28H remaining cholesterol protons), 0.89 (s, 3H), 0.88 (s, 3H), 0.84 (d, J = 6.4 Hz, 6H), 0.63 (s, 3H). HRMS calculated for $C_{249}H_{423}N_{13}O_{92}S_3$ $[M+2H]^{2+}$ 2582.9020, found 2579.7344.

1.3 Synthesis of ligand Bio$_3$-PEG-AIm

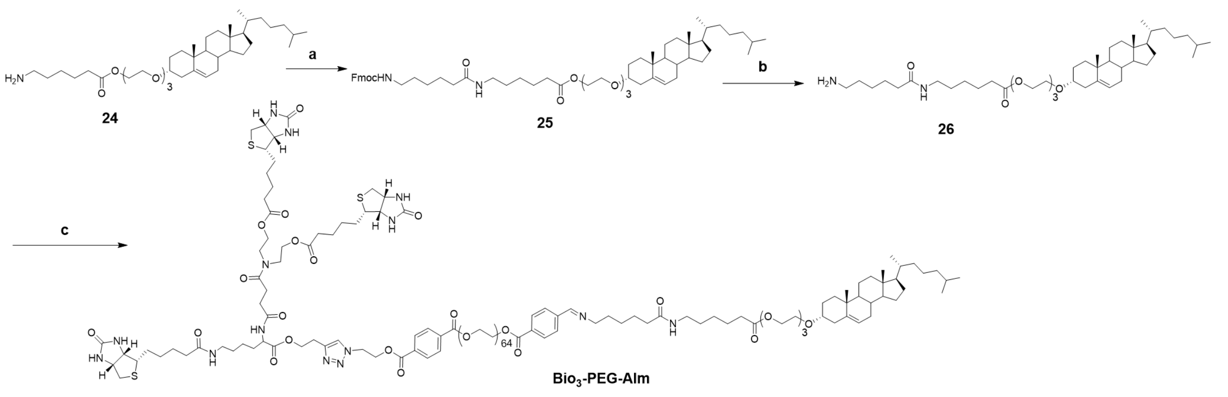

Scheme S2. The synthetic route of the ligand Bio_3-PEG-AIm. Reagents and conditions: (a) Fmoc-6-aminohexanoic acid, HATU, DIPEA, DCM, r.t., 2 h, 74.2%; (b) DBU, DCM, r.t., 30 min, 55.3%; (c) Compound **18**, AcOH, molecular sieves, DMSO, 40°C, 4 h, 75.6%.

*1.3.1 Synthesis of compound **24**.*

The synthesis of compound **24** had been reported in our previous work [1].

*1.3.2 Synthesis of compound **25**.*

To a solution of Fmoc-6-aminohexanoic acid (420.5 mg, 1.19 mmol) in dichloromethane (10 mL) was added HATU (539.7 mg, 1.42 mmol) and DIPEA (307.3 mg, 2.37 mmol), and the reaction was stirred at -5 °C for 30 min. Then, compound **24** (500 mg, 0.79 mmol) in dichloromethane (2 mL) was added slowly. After stirring for another 2h at room temperature, the mixture was washed with 1 mol/L HCl and extracted three times with dichloromethane. The combined organic layers were washed with water and brine, dried over anhydrous sodium sulfate, filtered, concentrated and purified by silica gel column chromatography to afford compound **25** as a yellowish oily (567.1 mg, 74.2%). ^{1}H-NMR (400 MHz, $CDCl_3$, ppm) δ 7.76 (d, J = 7.2 Hz, 2H), 7.59 (d, J = 7.2 Hz, 2H), 7.40 (t, J = 7.2 Hz, 2H), 7.31 (t, J = 7.2 Hz, 2H), 5.33-5.34 (m, 1H), 4.87 (brs, 1H), 4.78 (brs, 1H), 4.40 (s, 2H), 4.22 (s, 3H), 3.49-3.69 (m, 10H), 3.20-3.27 (m, 5H), 1.06-2.36 (44H, including 28H remaining cholesterol protons), 0.99 (s, 3H), 0.91 (d, J = 6.4 Hz, 3H), 0.86 (d, J = 6.8 Hz, 6H), 0.67 (s, 3H). ^{13}C-NMR (100 MHz, $CDCl_3$, ppm) δ 173.5, 156.4, 144.0, 141.3, 141.0, 127.6, 127.0, 125.0, 121.5, 120.0, 79.5, 70.9, 70.6, 70.4, 69.2, 67.3, 66.4, 56.8, 56.2, 50.2, 47.4, 42.3, 40.8, 39.8, 39.5, 39.1, 39.0, 37.3, 36.9, 36.2, 35.8, 34.0, 32.0, 31.9, 29.7, 29.6, 28.4, 28.3, 28.2, 28.0, 26.1, 24.5, 24.3, 23.8, 22.8, 22.6, 21.1, 19.4, 18.7, 11.9.

*1.3.3 Synthesis of compound **26**.*

DBU (2.36 g, 15.5 mmol) was added to the solution of compound **25** (5 g，5.17 mmol) in dichloromethane (50 mL). After stirred at room temperature for 30 min, the mixture was washed with 1 mol/L HCl, water and brine. Then, the combined organic layers were dried over anhydrous sodium sulfate, filtered and concentrated to afford compound **26** as a colorless oil (2.13 g, 55.3%), which was used for next step directly without further purification.

1.3.4 Synthesis of ligand Bio_3-PEG-AIm.

Compound **18** (42.5 mg, 0.01 mmol) and Compound **26** (11.2 mg, 0.015 mmol) were dissolved in anhydrous DMSO (1 mL). Then, molecular sieves (20 mg) and anhydrous AcOH (0.5 drop) were added under the protection of argon. After stirred at 40°C for 4h, the mixture was diluted with saturated sodium bicarbonate solution, filtered, and extracted three times with dichloromethane. The combined organic layers were washed with water and brine, dried over anhydrous sodium sulfate, filtered, concentrated and purified by dialysis and lyophilization to afford Bio_3-PEG-AIm as a white solid (37.6 mg, 75.6%). ^{1}H-NMR (400 MHz, DMSO-d_6, ppm) δ 8.42 (s, 1H), 8.16 (d, J = 7.6 Hz, 1H), 8.09 (s, 1H), 8.06 (d, J = 6.0 Hz, 2H), 8.02 (d, J = 8.0 Hz, 2H), 8.01 (d, J = 6.0 Hz, 2H), 7.95 (d, J = 8.0 Hz, 2H), 7.87 (s, 1H), 7.85 (s, 1H), 6.41 (s, 3H), 6.35 (s, 3H), 5.30 (s, 1H), 4.77 (t, J = 4.8 Hz, 2H), 4.68 (t, J = 4.8 Hz, 2H), 4.39-4.45 (m, 3H),

4.21-4.31 (m, 8H), 4.11-4.17 (m, 6H), 4.06 (t, J = 4.8 Hz, 2H), 3.76 (s, 4H), 3.68 (t, J = 4.8 Hz, 4H), 3.40-3.59 (m, 258H), 3.08-3.12 (m, 5H), 2.94-2.99 (m, 8H), 2.79-2.85 (m, 3H), 2.54-2.59 (m, 4H), 2.36-2.42 (m, 6H), 2.25-2.33 (m, 8H), 0.97-2.08 (62H, including 28H remaining cholesterol protons), 0.94 (s, 3H), 0.89 (s, 3H), 0.84 (d, J = 6.4 Hz, 6H), 0.65 (s, 3H).

1.4 Synthesis of ligand Bio$_3$-PEG-PIm

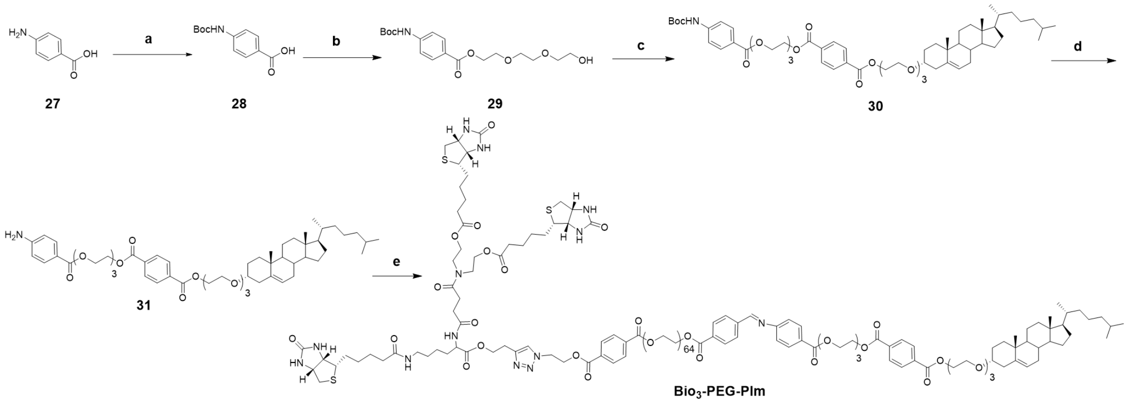

Scheme S3. The synthetic route of the ligand Bio$_3$-PEG-PIm. Reagents and conditions: (a) $(Boc)_2O$, Et_3N, 1,4-dioxane, H_2O, r.t., 24 h, 92.3%; (b) Triethylene glycol, EDCI, DMAP, DIPEA, DCM, r.t., 5 h, 78.5%; (c) Compound **21**, DCC, DMAP, DCM, r.t., 2 h, 74.4%; (d) CF_3COOH, DCM, r.t., 2 h, 72.8%; (e) Compound **18**, AcOH, molecular sieves, DMSO, 40°C, 4 h, 79.9%.

*1.4.1 Synthesis of compound **28**.*

Triethylamine (10 mL, 0.73 mol) was added to the solution of compound **27** (5.0 g, 0.36 mmol) in 1,4-dioxane (75 mL) and water (35 mL), and the reaction was stirred at room temperature for 5 min. Then, Di-tert-butyl dicarbonate (16 g, 0.73 mol) was added to the above solution. After stirring for another 24h at room temperature, the mixture was concentrated and 3N HCl was added to produce precipitation. After filtration, the filter cake was recrystallized with methanol to afford compound **28** as a white solid (7.95 g, 92.3%). M.p. 192-194°C. ^{1}H-NMR (400 MHz, DMSO-d_6, ppm) δ 12.61 (s, 1H), 9.73 (s, 1H), 7.83 (d, J = 8.8 Hz, 2H), 7.55 (d, J = 8.8 Hz, 2H), 1.48 (s, 9H).

*1.4.2 Synthesis of compound **29**.*

To a solution of compound **28** (250 mg, 1.05 mmol) in dichloromethane (5 mL) was added EDCI (303 mg, 1.58 mmol), DMAP (193 mg, 1.58 mmol) and DIPEA (272 mg, 2.1 mmol), and the reaction was stirred at -5 ^{o}C for 30 min. Then, triethylene glycol (396 mg, 2.63 mmol) in dichloromethane (1 mL) was added slowly. After stirring for another 5h at room temperature, the mixture was washed with 1 mol/L HCl and extracted three times with dichloromethane. The combined organic layers were washed with water and brine, dried over anhydrous sodium sulfate, filtered, concentrated and purified by silica gel column chromatography to afford compound **29** as a yellowish oily (304.5 mg, 78.5%). ^{1}H-NMR (400 MHz, $CDCl_3$, ppm) δ 7.98 (d, J = 8.8 Hz, 2H), 7.42 (d, J = 8.8 Hz, 2H), 6.77 (s, 1H), 4.46 (t, J = 4.8 Hz, 2H), 3.83 (t, J = 4.8 Hz, 2H), 3.67-3.72 (m, 6H), 3.60 (t, J = 4.8 Hz, 2H), 1.52 (s, 9H).

*1.4.3 Synthesis of compound **30**.*

To a solution of compound **21** (250 mg, 0.36 mmol) in dichloromethane (3 mL) was added DCC (155 mg, 0.72 mmol) and DMAP (9.5 mg, 0.07 mmol), and the reaction was stirred at -5 °C for 20 min. Then, compound **29** (199.5 mg, 0.54 mmol) in dichloromethane (0.5 mL) was added slowly. After stirring for another 2h at room temperature, the mixture was washed with 1 mol/L HCl and extracted three times with dichloromethane. The combined organic layers were washed with water and brine, dried over anhydrous sodium sulfate, filtered, concentrated and purified by silica gel column chromatography to afford compound **30** as a yellowish oily (272.7 mg, 74.4%). ^{1}H-NMR (400 MHz, $CDCl_3$, ppm) δ 8.08 (s, 4H), 7.96 (d, J = 8.8 Hz, 2H), 7.41 (d, J = 8.8 Hz, 2H), 6.83 (s, 1H), 5.31-5.32 (m, 1H), 4.47-4.51 (m, 4H), 4.44 (t, J = 4.8 Hz, 2H), 3.81-3.87 (m, 6H), 3.69-3.72 (m, 8H), 3.62 (s, 4H), 3.12-3.20 (m, 1H), 1.01-2.36 (28H, remaining cholesterol protons), 1.52 (s, 9H), 0.97 (s, 3H), 0.91 (d, J = 6.4 Hz, 3H), 0.86 (d, J = 6.4 Hz, 6H), 0.67 (s, 3H).

*1.4.4 Synthesis of compound **31**.*

Trifluoroacetic acid (1.5 mL, 30% V/V) was added to the solution of compound 30 (200 mg, 0.196 mmol) in dichloromethane (5 mL). After stirred at room temperature for 2h, the mixture was adjusted to pH 9 with saturated sodium bicarbonate solution and extracted three times with dichloromethane. The combined organic layers were washed with water and brine, dried over anhydrous sodium sulfate, filtered, concentrated and purified by silica gel column chromatography to afford compound **31** as a yellowish oily (131 mg, 72.8%). ^{1}H-NMR (400 MHz, $CDCl_3$, ppm) δ 8.09 (s, 4H), 7.84 (d, J = 8.4 Hz, 2H), 6.64 (d, J = 8.4 Hz, 2H), 5.30-5.32 (m, 1H), 4.47-4.51 (m, 4H), 4.41 (t, J = 4.8 Hz, 2H), 3.80-3.86 (m, 6H), 3.69-3.72 (m, 8H), 3.63 (s, 4H), 3.13-3.20 (m, 1H), 1.01-2.37 (28H, remaining cholesterol protons), 0.97 (s, 3H), 0.91 (d, J = 6.4 Hz, 3H), 0.86 (d, J = 6.4 Hz, 6H), 0.67 (s, 3H).

1.4.5 Synthesis of ligand Bio_3-PEG-PIm.

Compound **18** (42.5 mg, 0.01 mmol) and Compound **31** (13.8 mg, 0.015 mmol) were dissolved in anhydrous DMSO (1 mL). Then, molecular sieves (20 mg) and anhydrous AcOH (2 drops) were added under the protection of argon. After stirred at 40°C for 4h, the mixture was diluted with saturated sodium bicarbonate solution, filtered, and extracted three times with dichloromethane. The combined organic layers were washed with water and brine, dried over anhydrous sodium sulfate, filtered, concentrated and purified by dialysis and lyophilization to afford Bio_3-PEG-PIm as a white solid (41.1mg, 79.9%).

1.5 Synthesis of ligand Bio_3-PEG-Hz

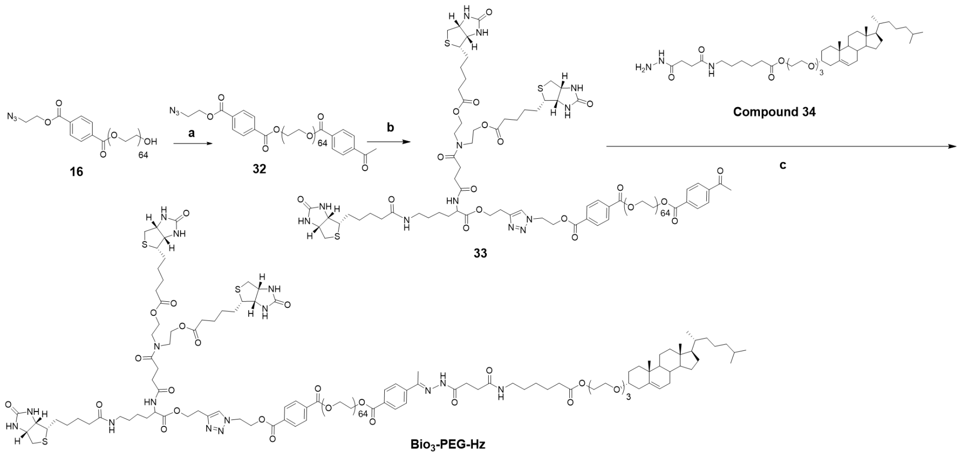

Scheme S4. The synthetic route of the ligand Bio_3-PEG-Hz. Reagents and conditions: (a) 4-Acetylbenzoic acid, DCC, DMAP, DCM, r.t., 24 h, 83.6%; (b) Compound **12**, sodium ascorbate, CuI, DMF/H_2O, r.t., 16 h, 57.2%; (c) Compound **35**, HCOOH, DCM, THF, 20°C, 72 h, 60.3%.

*1.5.1 Synthesis of compound **32**.*

To a solution of 4-acetylbenzoic acid (74.42 mg, 0.45 mmol) in dichloromethane (5 mL) was added DCC (123.6 mg, 0.60 mmol) and DMAP (7.33 mg, 0.06 mmol), and the reaction was stirred at -5 ^{o}C for 30 min. Then, compound **16** (915.4 mg, 0.3 mmol) in dichloromethane (3 mL) was added slowly. After stirring for another 24h at room temperature, the mixture was cooled at -20 ^{o}C for 5h and filtered through a Buchner funnel. The filtrate was washed with 1 mol/L HCl and extracted three times with dichloromethane. The combined organic layers were washed with water and brine, dried over anhydrous sodium sulfate, filtered, concentrated and purified by recrystallization (DCM/Et_2O) to afford compound **32** as a white solid (801.9 mg, 83.6%). M.p. 95.8-97.2^{o}C. ^{1}H-NMR (400 MHz, DMSO-d_6, ppm) δ 8.11 (s, 4H), 8.08 (s, 4H), 4.49 (t, J = 4.8 Hz, 2H), 4.43 (t, J = 4.0 Hz, 4H), 3.76 (t, J = 4.8 Hz, 4H), 3.67-3.72 (m, 4H), 3.48-3.61 (m, 246H), 2.63 (s, 3H).

*1.5.2 Synthesis of compound **33**.*

To a solution of compound **12** (100 mg, 0.094 mmol) and compound **32** (450.9 mg, 0.141 mmol) in the mixed solvent of DMF (3 mL) and water (0.3 mL) was added CuI (5.3 mg, 0.028 mmol) and sodium ascorbate (11.1 mg, 0.056 mmol). After stirring for another 16h at room temperature under the protection of argon, the mixture was concentrated and purified by silica gel column chromatography to afford compound **33** as a yellowish oily (229.1 mg, 57.2%). ^{1}H-NMR (400 MHz, DMSO-d_6, ppm) δ 8.15-8.17 (m, 1H), 8.11 (s, 1H), 8.05-8.08 (m, 6H), 8.02 (d, J = 8.4 Hz, 2H), 7.71 (t, J = 5.6 Hz, 1H), 6.41 (s, 3H), 6.35 (s, 3H), 4.76-4.78 (m, 2H), 4.67-4.69 (m, 2H), 4.41-4.42 (m, 3H), 4.22-4.36 (m, 6H), 4.13-4.17 (m, 6H), 4.06-4.08 (m, 2H), 3.76 (s, 2H), 3.68 (t, J = 4.8 Hz, 2H), 3.37-3.59 (m, 252H), 3.08-3.10 (m, 3H), 2.94-3.01 (m, 5H), 2.80-2.86 (m, 3H), 2.63 (s, 3H), 2.59 (s, 2H), 2.56 (s, 2H), 2.36-2.42 (m, 2H), 2.25-2.32 (m, 6H), 2.03 (t, J = 6.8 Hz, 2H), 1.48-1.59 (m, 14H), 1.23-1.31 (m, 8H).

*1.5.3 Synthesis of compound **34**.*

The synthesis of compound **34** had been reported in our previous work.[1]

1.5.4 Synthesis of ligand Bio3-PEG-Hz.

Compound **33** (42.6 mg, 0.01 mmol) was dissolved in a mixture of anhydrous dichloromethane (1 mL) and tetrahydrofuran (0.9 mL). Then, compound **34** (45 mg, 0.06 mmol) in anhydrous dichloromethane (1 mL) was added dropwise into the above solution under the protection of argon. After that, anhydrous formic acid (1 drop) was added and the mixture was stirred at 20 ^oC for 72h. At last, the mixture was concentrated and purified by flash column chromatography to afford ligand Bio$_3$-PEG-Hz as a faint yellow oily (29.6 mg, 60.3%). ^{1}H-NMR (400 MHz, DMSO-*d*$_6$, ppm) δ 10.56 (s, 1H), 8.17 (d, J = 7.2 Hz, 1H), 8.11 (s, 1H), 8.07 (d, J = 7.6 Hz, 2H), 8.02 (d, J = 8.4 Hz, 2H), 7.98 (d, J = 8.4 Hz, 2H), 7.91 (d, J = 7.6 Hz, 2H), 7.80 (s, 1H), 7.73 (t, J = 5.6 Hz, 1H), 6.41 (s, 3H), 6.35 (s, 3H), 5.30 (s, 1H), 4.77 (t, J = 4.8 Hz, 2H), 4.68 (t, J = 4.8 Hz, 2H), 4.38-4.42 (m, 3H), 4.21-4.30 (m, 6H), 4.11-4.17 (m, 8H), 4.06 (t, J = 4.8 Hz, 2H), 3.76 (s, 2H), 3.68 (t, J = 4.8 Hz, 2H), 3.51-3.59 (m, 262H), 3.07-3.10 (m, 4H), 2.93-3.02 (m, 7H), 2.79-2.85 (m, 3H), 2.54-2.59 (m, 4H), 2.37-2.41 (m, 5H), 2.26-2.32 (m, 12H), 0.97-2.08 (58H, including 28H remaining cholesterol protons), 0.94 (s, 3H), 0.89 (d, J = 6.0 Hz, 3H), 0.84 (d, J = 6.4 Hz, 6H), 0.65 (s, 3H).

1.6 Synthesis of ligand Bio3-PEG-BSa

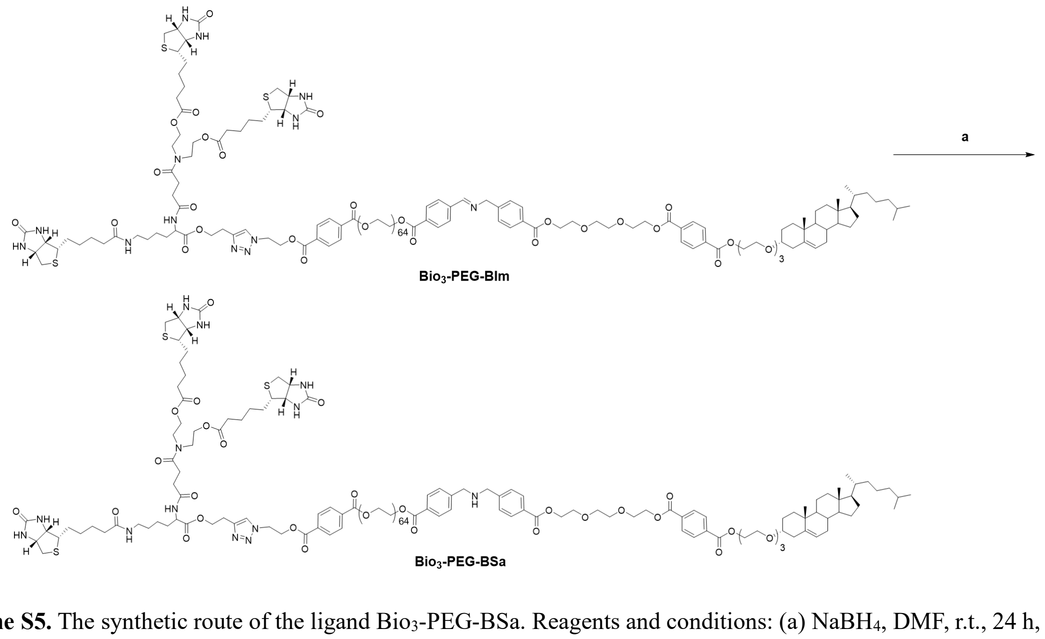

Scheme S5. The synthetic route of the ligand Bio$_3$-PEG-BSa. Reagents and conditions: (a) $NaBH_4$, DMF, r.t., 24 h, 81.2%.

$NaBH_4$ (5.7 mg, 0.15 mmol) was slowly added to the solution of Bio$_3$-PEG-BIm (154.8 mg, 0.03 mmol) in anhydrous DMF (1 mL) at 0°C under the protection of argon. After stirring for 24h at room temperature, the mixture was diluted with saturated sodium bicarbonate solution and extracted three times with dichloromethane. The combined organic layers were washed with water and brine, dried over anhydrous sodium sulfate, filtered, concentrated and purified by flash column chromatography to afford Bio$_3$-PEG-BSa as a faint yellow oily (125.8 mg, 81.2%). ^{1}H-NMR

(400 MHz, DMSO-d_6, ppm) δ 8.18 (d, J = 7.2 Hz, 1H), 8.07 (s, 5H), 8.02 (d, J = 7.6 Hz, 2H), 7.92 (d, J = 8.0 Hz, 2H), 7.89 (d, J = 7.6 Hz, 2H), 7.73 (t, J = 5.6 Hz, 1H), 7.53 (d, J = 8.0 Hz, 1H), 7.51 (d, J = 7.6 Hz, 1H), 7.46 (d, J = 8.0 Hz, 2H), 7.38 (d, J = 7.6 Hz, 1H), 7.36 (d, J = 8.0 Hz, 1H), 6.42 (s, 3H), 6.36 (s, 3H), 5.25 (s, 1H), 4.89 (brs, 1H), 4.77 (d, J = 4.4 Hz, 2H), 4.68 (t, J = 4.4 Hz, 2H), 4.57 (t, J = 5.6 Hz, 4H), 4.35-4.41 (m, 10H), 4.28-4.32 (m, 3H), 4.12-4.26 (m, 4H), 4.06 (t, J = 4.8 Hz, 2H), 3.75 (s, 12H), 3.68 (t, J = 4.8 Hz, 2H), 3.58-3.61 (m, 12H), 3.41-3.50 (m, 252H), 3.08 (s, 4H), 2.94-2.98 (m, 5H), 2.81 (dd, J_1 = 12.8 Hz, J_2 = 4.4 Hz, 3H), 2.55-2.59 (m, 4H), 2.38-2.42 (m, 2H), 2.25-2.32 (m, 7H), 2.01-2.05 (m, 3H), 1.05-1.94 (48H, including 28H remaining cholesterol protons), 0.89 (s, 6H), 0.84 (d, J = 6.40 Hz, 6H), 0.63 (s, 3H).

2. The hydrolysis of ligands Bio$_3$-PEG-BIm, Bio$_3$-PEG-AIm, Bio$_3$-PEG-PIm and Bio$_3$-PEG-Hz

To test the responsiveness of different acid-sensitive bonds to the tumor microenvironment, ligands Bio$_3$-PEG-BIm, Bio$_3$-PEG-Hz, Bio$_3$-PEG-AIm and Bio$_3$-PEG-PIm was treated with the follow conditions: (A) the PBS (pH 7.4) solution of the ligand Bio$_3$-PEG-BIm or Bio$_3$-PEG-Hz or Bio$_3$-PEG-PIm (10 mg/mL) was shaken at 37 °C for 24h; (B) the PBS (pH 7.4) solution of the ligand Bio$_3$-PEG-AIm (10 mg/mL) was shaken at 37 °C for 2h; (C) the PBS (pH 6.5) solution of the ligand Bio$_3$-PEG-Hz or Bio$_3$-PEG-PIm (10 mg/mL) was shaken at 37 °C for 2h; (D) the PBS (pH 6.5) solution of the ligand Bio$_3$-PEG-BIm (10 mg/mL) was shaken at 37 °C for 1h; (E) the ligand Bio$_3$-PEG-BIm (10 mg) was added into the PBS (pH 6.5, 1 mL) solution of GOx (100 µg/mL) and glucose (1 mg/mL), then shaken at 37 °C for 1h. Subsequently, using the same method for reprocessing: (1) Each solution was diluted with saturated sodium bicarbonate solution and extracted three times with dichloromethane. (2) The combined organic layers were washed with water and brine, dried over anhydrous sodium sulfate, filtered, concentrated, and then identified by ^{1}H NMR.

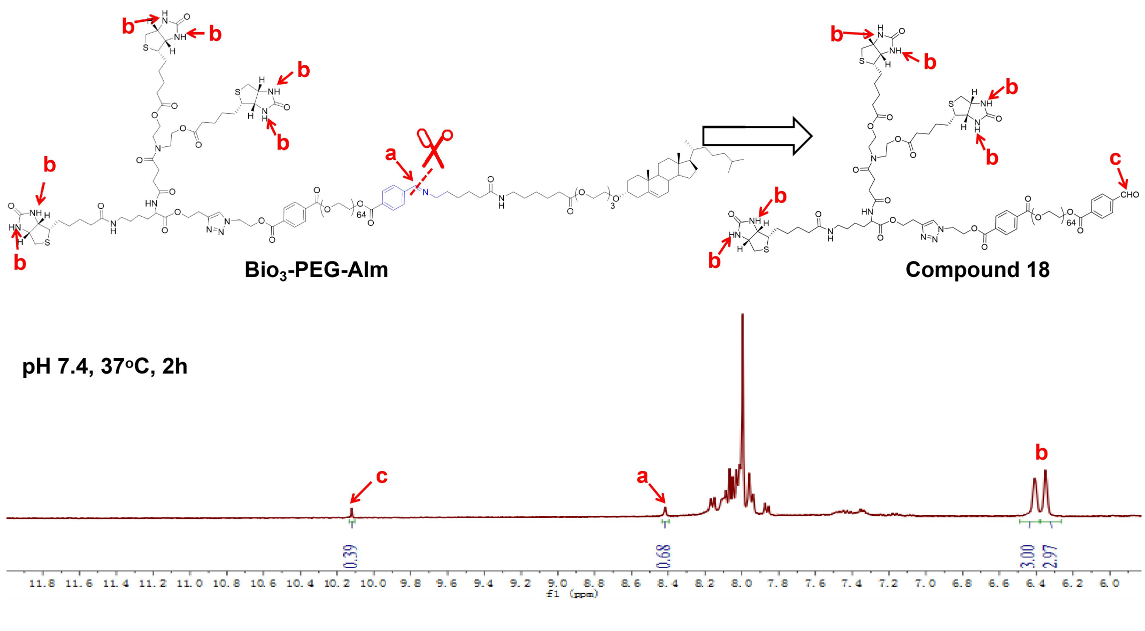

Figure S1. ^{1}H NMR spectra of Bio$_3$-PEG-AIm after treatment by the follow condition: PBS (pH 7.4), 37 °C, 2h.

As shown in Figure S1, after shaking at pH 7.4 for 2h, about 36.4% (0.39/(0.39+0.68)×100%) of Bio3-PEG-AIm was hydrolyzed, indicating that the alkyl-imine bond (AIm) was not stable under normal physiological conditions of blood circulation and could not be used as acid-sensitive bond in guidance unit.

After Bio3-PEG-PIm was oscillated at pH 7.4 for 24 hours, there was still no new signal peak in the corresponding hydrogen spectrum (Figure S2A), indicating that this ligand remained stable for a long time under normal physiological conditions of blood circulation. In contrast, about 18.7% (0.20/(0.20+0.87)×100%) of Bio3-PEG-PIm was hydrolyzed at pH 6.5 for 2 h (Figure S2B), indicating that phenyl-imine bond (PIm) was slowly broken under weakly acidic conditions in the tumor microenvironment.

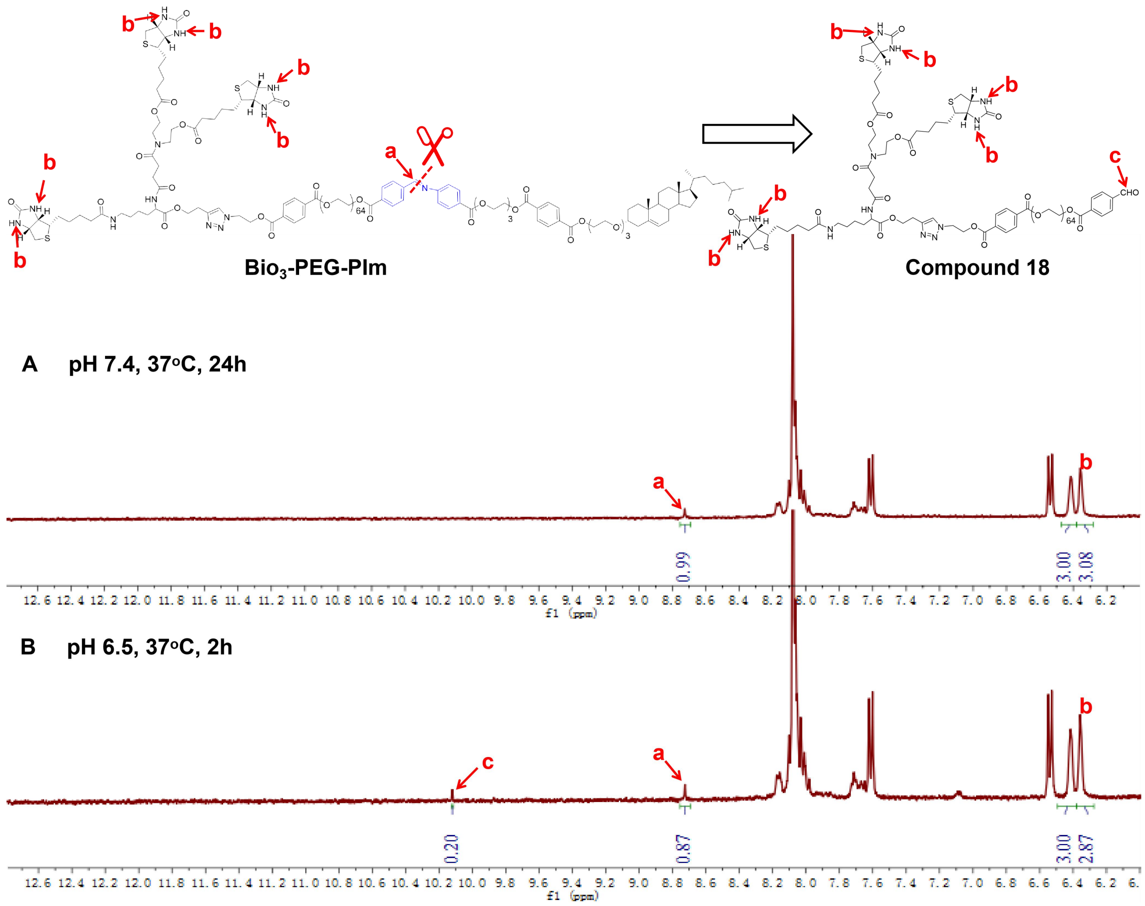

Figure S2. ^{1}H NMR spectra of Bio3-PEG-PIm after treatment by different conditions: (A) PBS (pH 7.4), 37 °C, 24h; (B) PBS (pH 6.5), 37 °C, 2h.

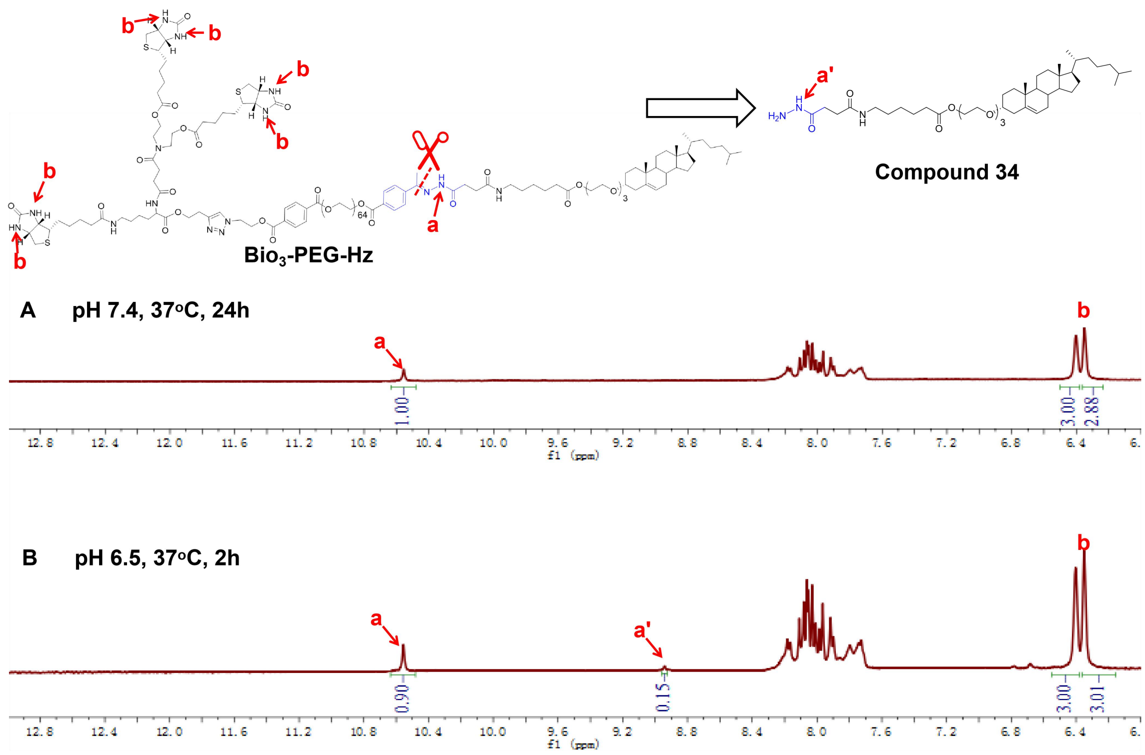

Figure S3. ^{1}H NMR spectra of Bio$_3$-PEG-Hz after treatment by different conditions: (A) PBS (pH 7.4), 37 °C, 24h; (B) PBS (pH 6.5), 37 °C, 2h.

Similarly, no new signal peak appeared in the hydrogen spectrum of Bio$_3$-PEG-Hz after shock at pH 7.4 for 24h (Figure S3A). In contrast, about 14.3% (0.15/(0.15+0.90)×100%) of Bio$_3$-PEG-Hz was hydrolyzed at pH 6.5 for 2h (Figure S3B), indicating that the hydrazone bond (Hz) fractured slowly under weakly acidic conditions in tumor microenvironment, and the rate was slower than that of Bio$_3$-PEG-PIm.

Table S1. The hydrolysis rate of ligands Bio$_3$-PEG-Hz, Bio$_3$-PEG-AIm, Bio$_3$-PEG-PIm and Bio$_3$-PEG-BIm in different conditions.

Ligand	Hydrolysis rate at pH 7.4 [a]	Hydrolysis rate at pH 6.5 [b]
Bio$_3$-PEG-Hz	0	14.3%
Bio$_3$-PEG-AIm	36.4% [c]	N.T. [d]
Bio$_3$-PEG-PIm	0	18.7%
Bio$_3$-PEG-BIm	0	47.7% [e]

[a] pH 7.4, 24h; [b] pH 6.5, 2h; [c] pH 7.4, 2h; [d] Not tested;[e] pH 6.5, 1h.

3. Cell culture

4T1 cells (Murine TNBC cell line) were maintained in RPMI 1640, whereas MDA-MB-231 cells (Human TNBC cancer cell line), MCF7 ((Human breast cancer cell line) and L02 cells (Human hepatocytes with normally-expressed SMVT) were cultivated in Dulbecco's Modified Eagles Medium (DMEM), all supplemented with 10% FBS, 100 U/mL

streptomycin, and 100 U/mL penicillin at 37 °C under a humidified atmosphere containing 5% CO_2.

4. Animals

Female BALB/c mice (6–8 weeks old, SPF) were bought from Dashuo Biotechnology Co., Ltd (Chengdu, China). All animal procedures were performed in accordance with the Guidelines for the Care and Use of Laboratory Animals of Sichuan University, and approved by the Animal Ethics Committee of Sichuan University.

5. Other experimental results

Table S2. Characterization of different nano-rockets. (means ± SD, n = 3).

Nano-rockets (NR)	pH	Size (nm)	PDI	Zeta potential (mV)
LF-NR	7.4	113.4±1.3	0.190±0.016	-1.24±0.50
	6.5	114.1±1.5	0.173±0.022	-0.57±0.39
NH_2-NR	7.4	119.7±2.1	0.198±0.034	28.8±0.62
	6.5	120.8±1.6	0.193±0.030	39.7±0.41
Bio_3-BSa-NR	7.4	151.6±1.8	0.185±0.019	-15.9±0.55
	6.5	151.0±2.3	0.153±0.028	-14.7±0.37
Bio_3-BIm-NR	7.4	152.2±1.6	0.162±0.025	-16.7±0.26
	6.5	126.9±2.3	0.177±0.029	30.8±0.55

Table S3. Characterization of GOx+L-Arg-NM and PTX-NM. (means ± SD, n = 3).

Nano-missiles (NM)	Size(nm)	PDI	EE (%)	Zeta potential (mV)
GOx+L-Arg-NM	173.6±0.9	0.111±0.019	11.06±1.09（GOx）<br>53.74±1.83（L-Arg）	-18.22±0.46
PTX-NM	155.3±1.5	0.134±0.025	92.79±2.22	-15.02±0.34

Table S4. IC_{50} values of PTX to 4T1, MCF7 and MDA-MB-231 cells (means ± SD, n = 6).

Cell Lines	IC_{50} (μg/mL)		
	PTX-NM	GOx+L-Arg-NM	PTX-NM& GOx+L-Arg-NM
4T1	0.89	/	0.08
MCF7	1.13	/	0.10
MDA-MB-231	3.27	/	0.09

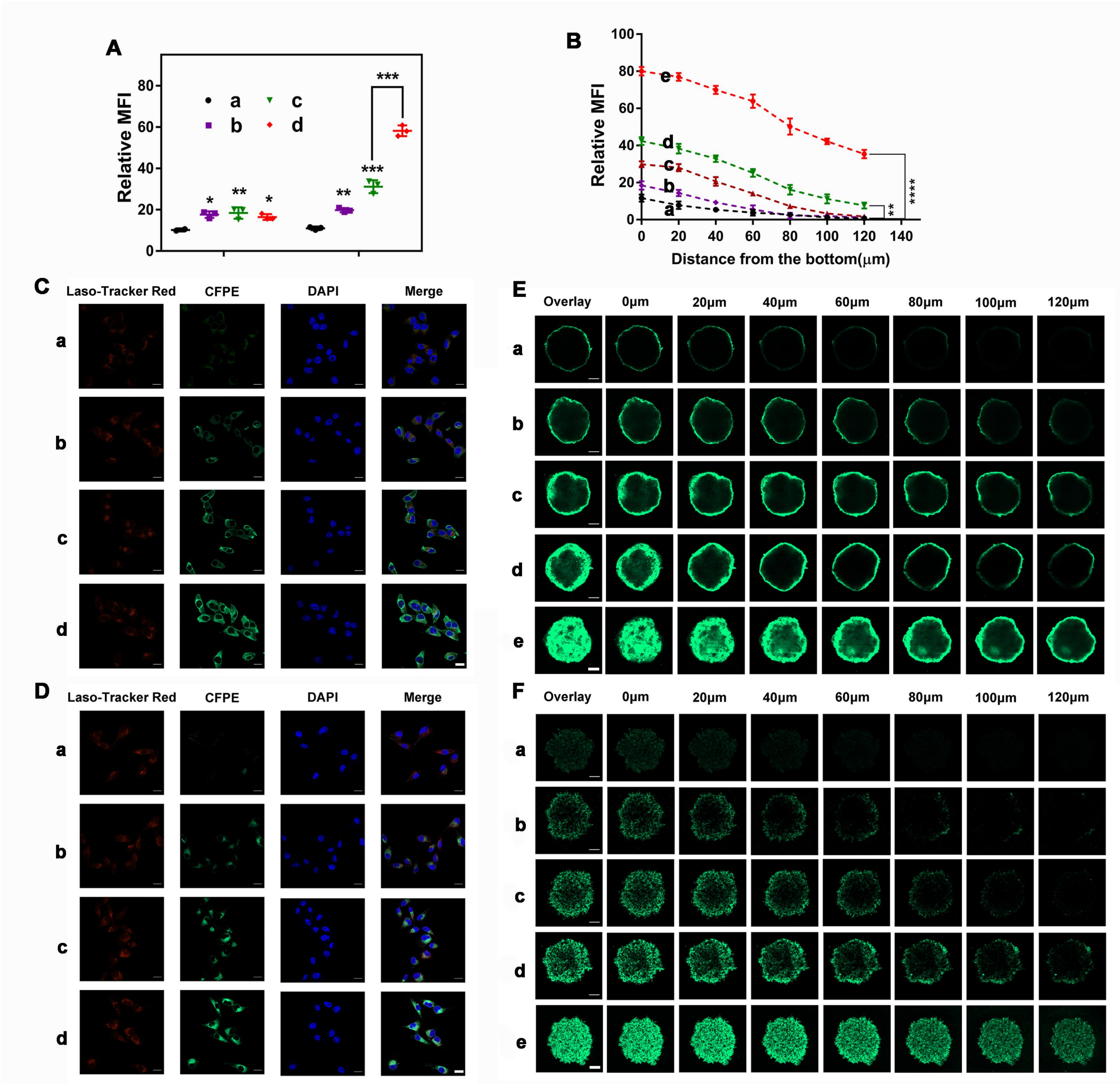

Figure S4. (**A**) The quantitative cellular internalization of MDA-MB-231 cells after incubation with nano-rockets at pH 7.4 (left) or pH 6.5 (right), respectively ((a) CFPE-LF-NR, (b) CFPE-Bio_3-BSa-NR, (c) CFPE-Bio_3-BIm-NR, (d) CFPE-Bio_3-BIm-NR&GOx+L-Arg-NM) (means ± SD, n = 3, * indicates $p < 0.05$, ** indicates $p < 0.01$, *** indicates $p < 0.001$). (**B**) was the semi-quantitative intensity of these nano-rockets in MDA-MB-231 tumor spheroids ((a) CFPE-LF-NR, (b) CFPE-Bio_3-BSa-NR, (c) CFPE-Bio_3-BSa-NR&GOx+L-Arg-NM, (d)

CFPE-Bio_3-BIm-NR, (e) CFPE-Bio_3-BIm-NR&GOx+L-Arg-NM) (means ± SD, n = 3, ** indicates $p < 0.01$, **** indicates $p < 0.0001$ versus CFPE-LF-NR). Confocal laser scanning microscopy (CLSM) images on (**C**) 4T1 cells and (**D**) MDA-MB-231 cells at pH 6.5, showing FITC channel (green), LysoTracker-stained lysosome channel (red), and DAPI-stained nucleus channel (blue), the scale bar = 20 μm ((a) CFPE-LF-NR, (b) CFPE-Bio_3-BSa-NR, (c) CFPE-Bio_3-BIm-NR, (d) CFPE-Bio_3-BIm-NR&GOx+L-Arg-NM) (n = 3). (**E**) and (**F**) were the fluorescence distribution of 4T1 and MDA-MB-231 tumor spheroids under pH 6.5 conditions, respectively ((a) CFPE-LF-NR, (b) CFPE-Bio_3-BSa-NR, (c) CFPE-Bio_3-BSa-NR&GOx+L-Arg-NM, (d) CFPE-Bio_3-BIm-NR, (e) CFPE-Bio_3-BIm-NR&GOx+L-Arg-NM) (n = 3). The scale bar = 100 μm.

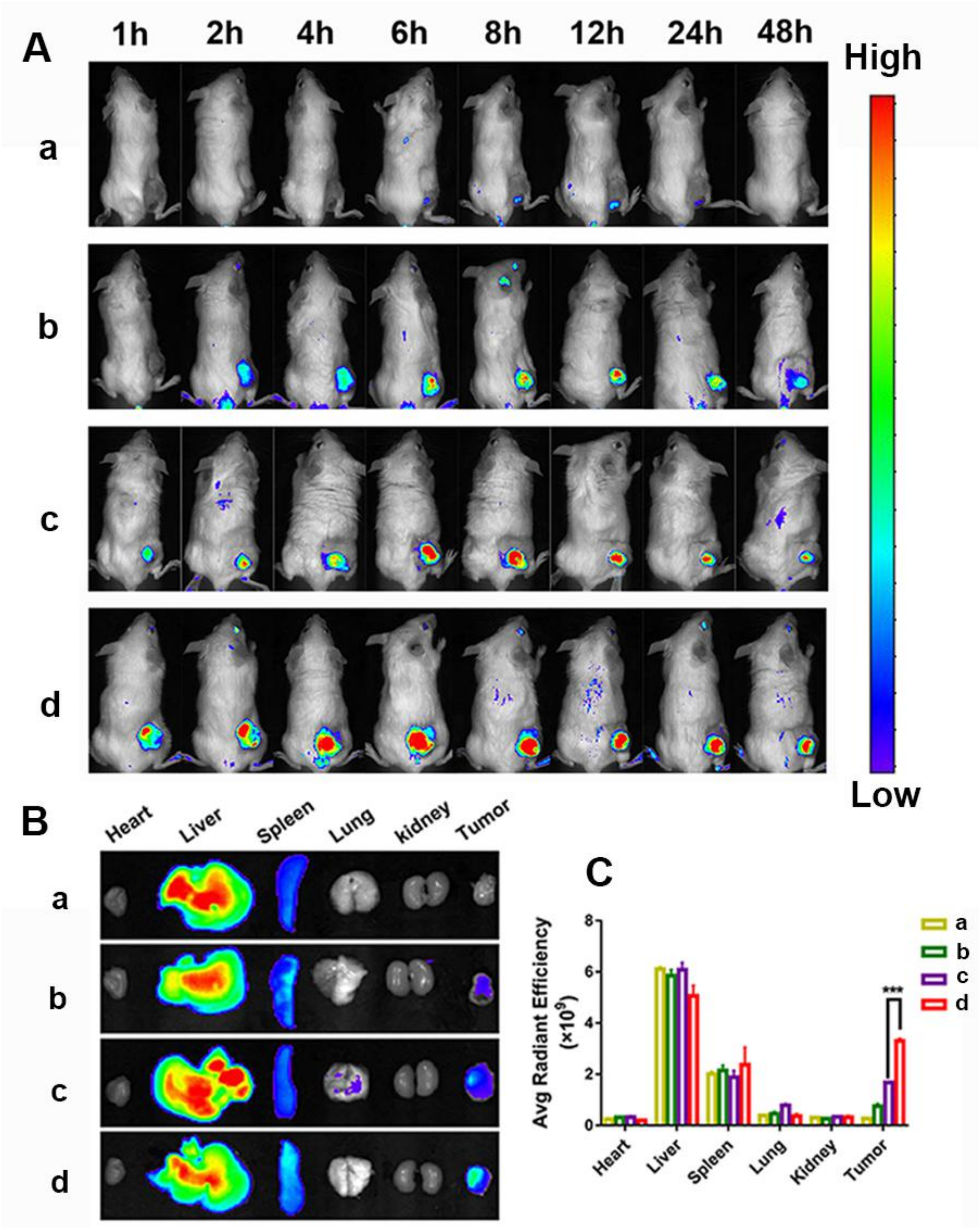

Figure S5. (**A**) In vivo fluorescence imaging of the 4T1 tumor xenografts bearing mice after intravenous injection with DiD-LF-NR (a), DiD-Bio_3-BSa-NR (b), DiD-Bio_3-BIm-NR (c), and DiD-Bio_3-BSa-NR&GOx+L-Arg-NM (d). (**B**) Ex vivo imaging of the major organs and tumor ex vivo 24 h post-injection. (**C**) Radiant efficiency of the organs measured by ex vivo imaging (means ± SD, *** indicates $p < 0.001$).

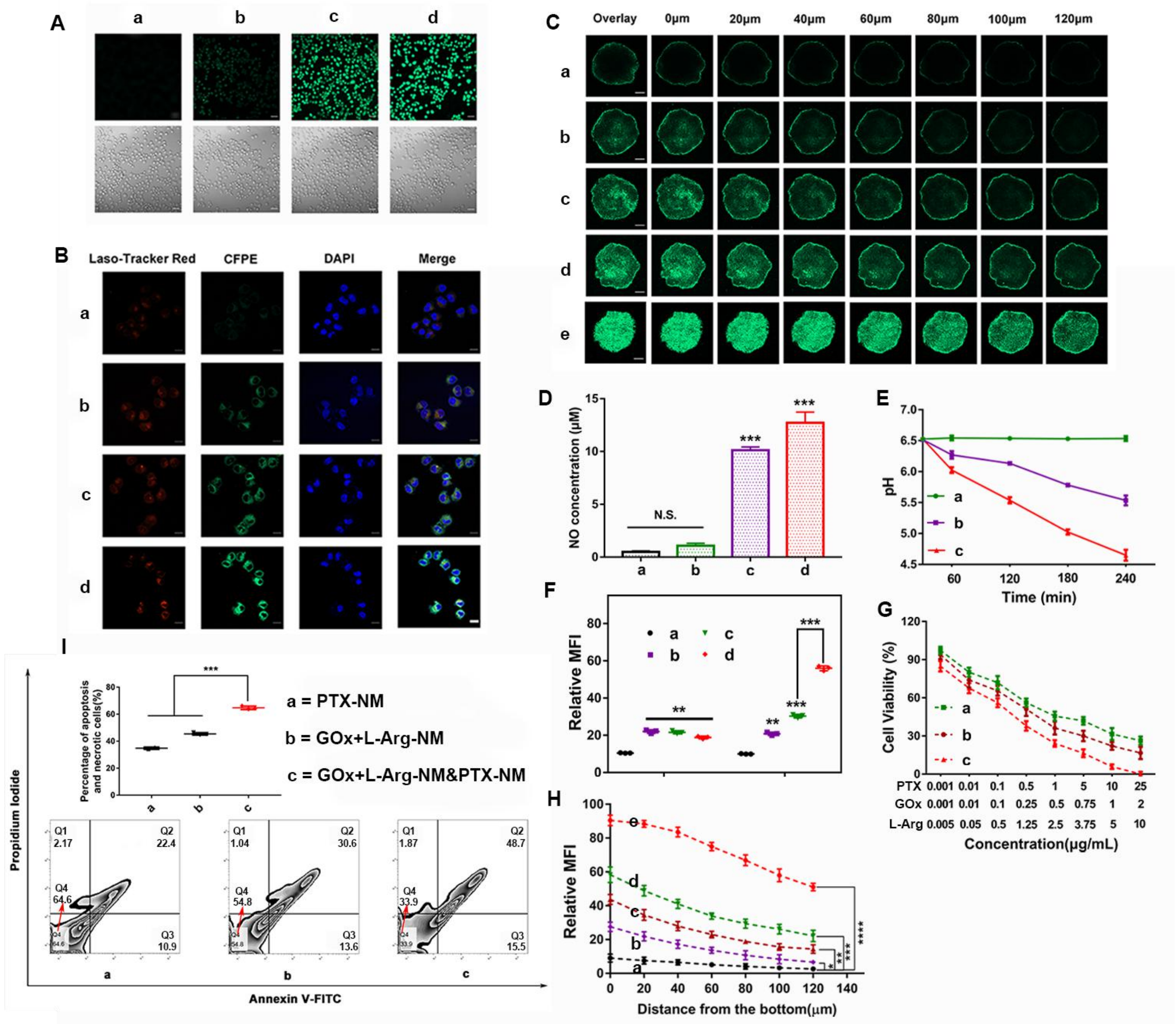

Figure S6. Confocal fluorescence and bright field images (**A**), NO generation (**D**) of MCF7 cells after incubation with different nano-missiles for 6 h ((a) control, (b) PTX-NM, (c) GOx+L-Arg-NM, (d) GOx+L-Arg-NM&PTX-NM) (means ± SD, n = 3, *** indicates $p < 0.001$, N.S. indicates no significant difference). (**E**) Time-dependent changes in the pH values of MCF7 cells at pH 6.5 ((a) control, (b) GOx+L-Arg-NM (GOx: 10 μg/mL, glucose: 0 mg/mL), (c) GOx+L-Arg-NM (GOx: 10 μg/mL, glucose: 1 mg/mL)) (means ± SD, n = 3). (**B**) Confocal laser scanning microscopy (CLSM) images on MCF7 cells at pH 6.5, showing FITC channel (green), LysoTracker-stained lysosome channel (red), and DAPI-stained nucleus channel (blue), the scale bar = 20 μm (n = 3). (**F**) The quantitative cellular internalization of MCF7 cells after incubation with nano-rockets at pH 7.4 or pH 6.5, respectively ((a) CFPE-LF-NR, (b) CFPE-Bio_3-BSa-NR, (c) CFPE-Bio_3-BIm-NR, (d) CFPE-Bio_3-BIm-NR&GOx+L-Arg-NM) (means ± SD, n = 3, ** indicates $p < 0.01$, *** indicates $p < 0.001$). (**C**) was the fluorescence distribution and (**H**) was the semi-quantitative intensity of these nano-rockets in MCF7 tumor spheroids under pH 6.5 conditions ((a) CFPE-LF-NR, (b) CFPE-Bio_3-BSa-NR, (c) CFPE-Bio_3-BSa-NR&GOx+L-Arg-NM, (d) CFPE-Bio_3-BIm-NR, (e) CFPE-Bio_3-BIm-NR&GOx+L-Arg-NM) (means ± SD, n = 3, * indicates $p < 0.05$, ** indicates $p < 0.01$, *** indicates $p < 0.001$, **** indicates $p < 0.0001$ versus CFPE-LF-NR). (**G**) The cytotoxicity study of different nano-missiles on MCF7 cells ((a) PTX-NM, (b) GOx+L-Arg-NM, (c) GOx+L-Arg-NM&PTX-NM) (means ± SD, n = 6). (**I**) Flow cytometry analysis and the apoptotic proportion of MCF7 cells after incubation with PTX-NM, GOx+L-Arg-NM, GOx+L-Arg-NM&PTX-NM (means ± SD, n = 3, *** indicates $p < 0.001$).

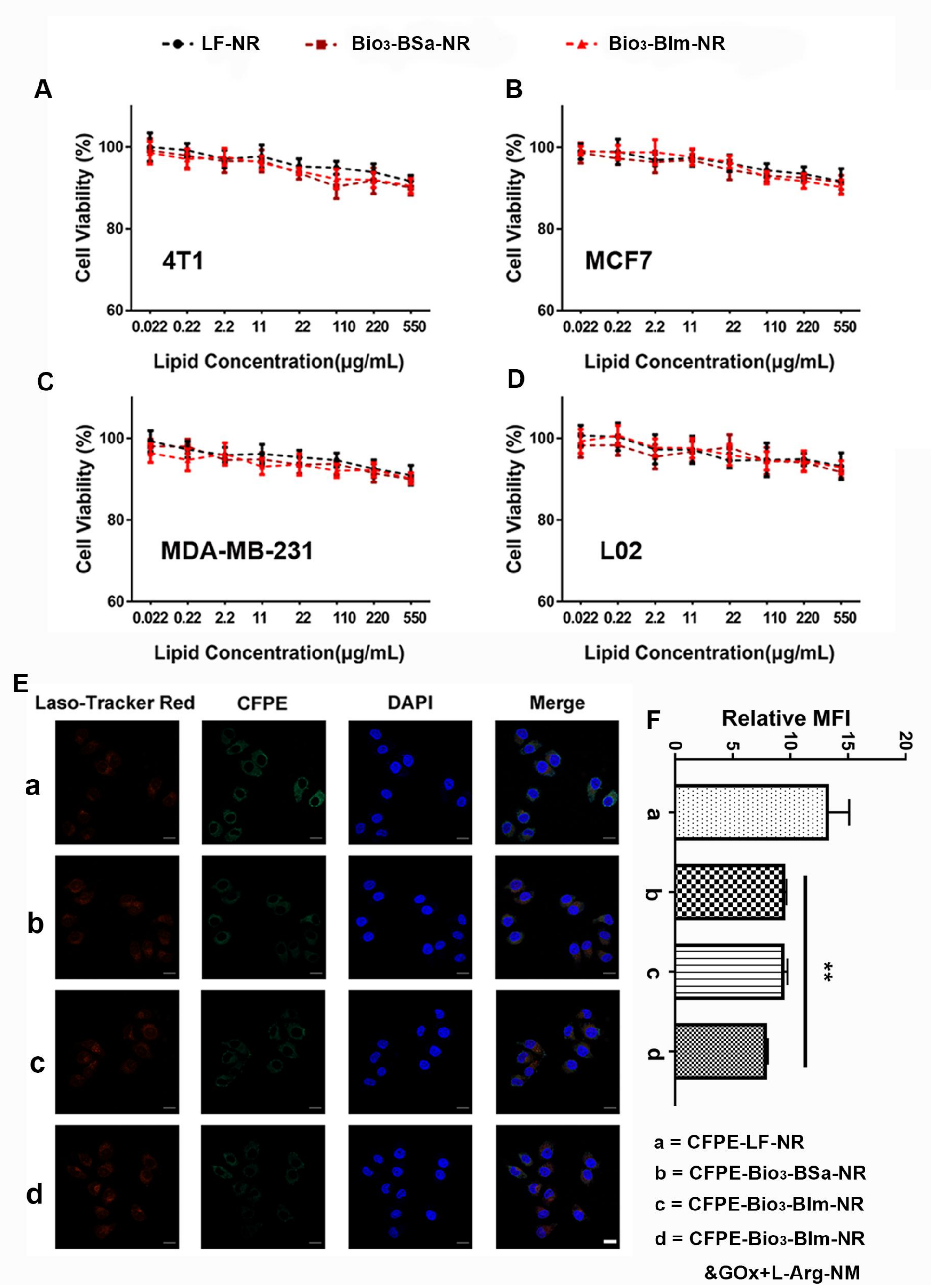

Figure S7. The cytotoxicity study of nano-rockets without ammunitions on (**A**) 4T1, (**B**) MCF7, (**C**) MDA-MB-231, (**D**) L02 cells (means ± SD, n = 6). (**E**) Confocal laser scanning microscopy (CLSM) images on L02 cells after incubation with different nano-rockets for 1 h, the scale bar = 20 µm (n = 3). (**F**) the quantitative cellular internalization of L02 cells after incubation with nano-rockets at pH 7.4 (means ± SD, n = 3, ** indicates p < 0.01 versus CFPE-LF-NR).

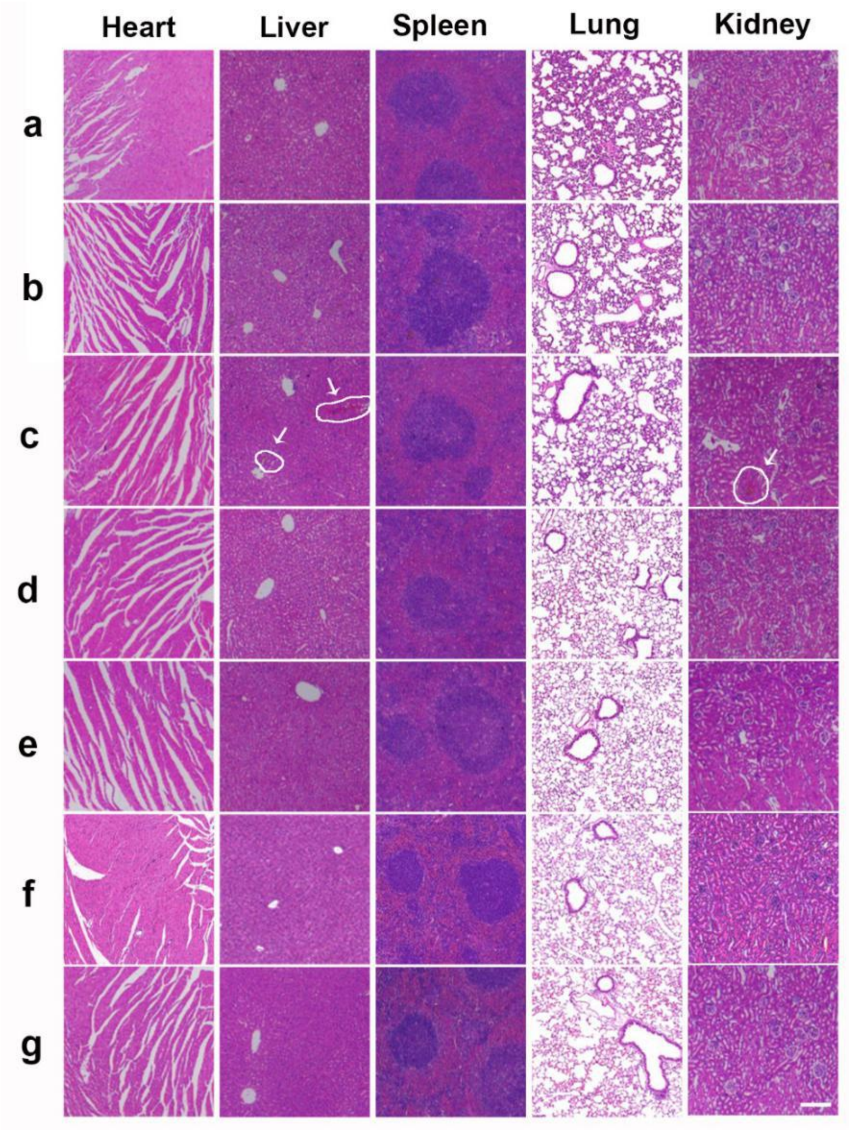

Figure S8. H&E analyses of the major organs (heart, liver, spleen, lung and kidney) after treatments of PBS (a), Free PTX (b), Free GOx+L-Arg (c), PTX-NM (d), GOx+L-Arg-NM (e), GOx+L-Arg-NM&PTX-NM (PTX = 3 mg/kg) (f), GOx+L-Arg-NM&PTX-NM (PTX = 1 mg/kg) (g). The white circle and arrow represent the blood cells in the intercellular space, and the scale bar = 200 μm.

6. Reference

1. Lu RX, Zhou L, Liu QJ, Wang SQ, Yang CY, Hai L, et al. Skillfully collaborating chemosynthesis with GOx-enabled tumor survival microenvironment deteriorating strategy for amplified chemotherapy and enhanced tumor ablation. Biomater Sci. 2021; 9: 1855-1871.

Figure S9. ^{1}H-NMR of compound **2**

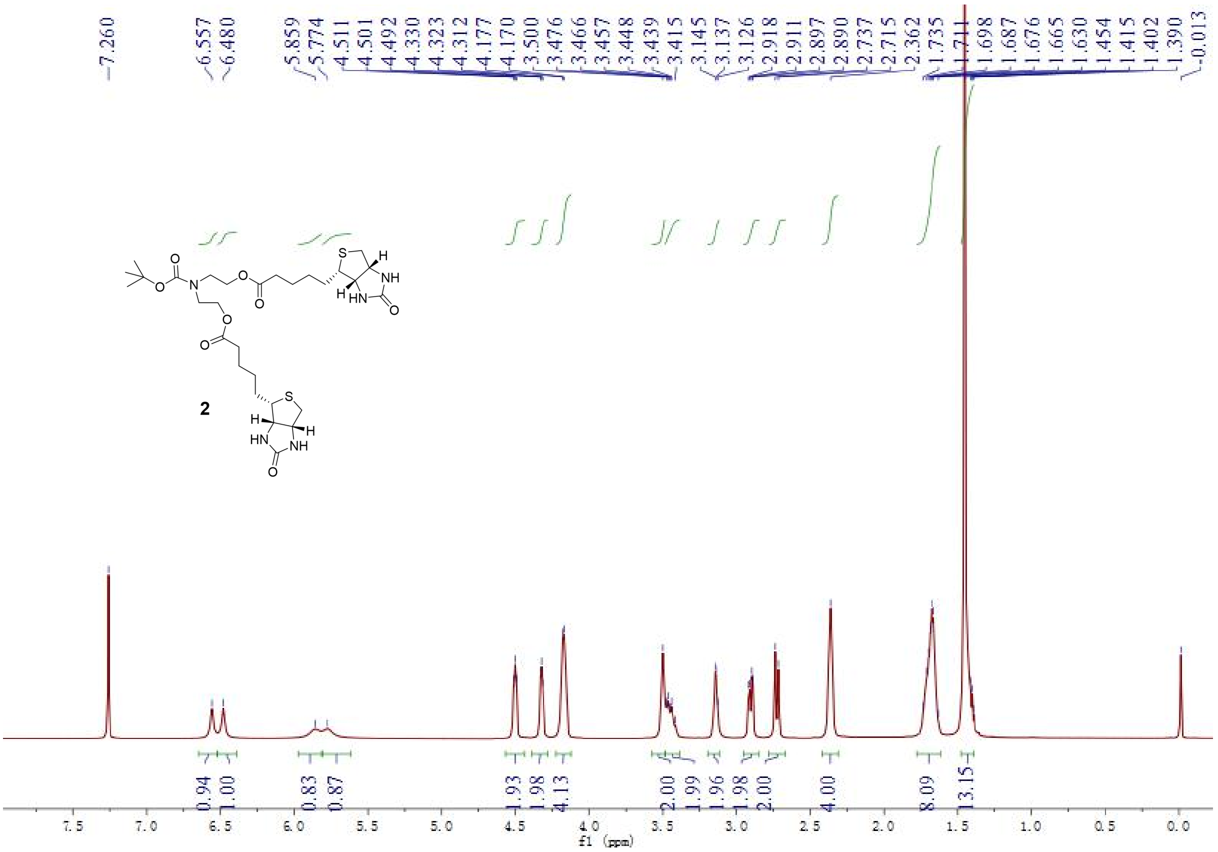

Figure S10. ^{13}C-NMR of compound **2**

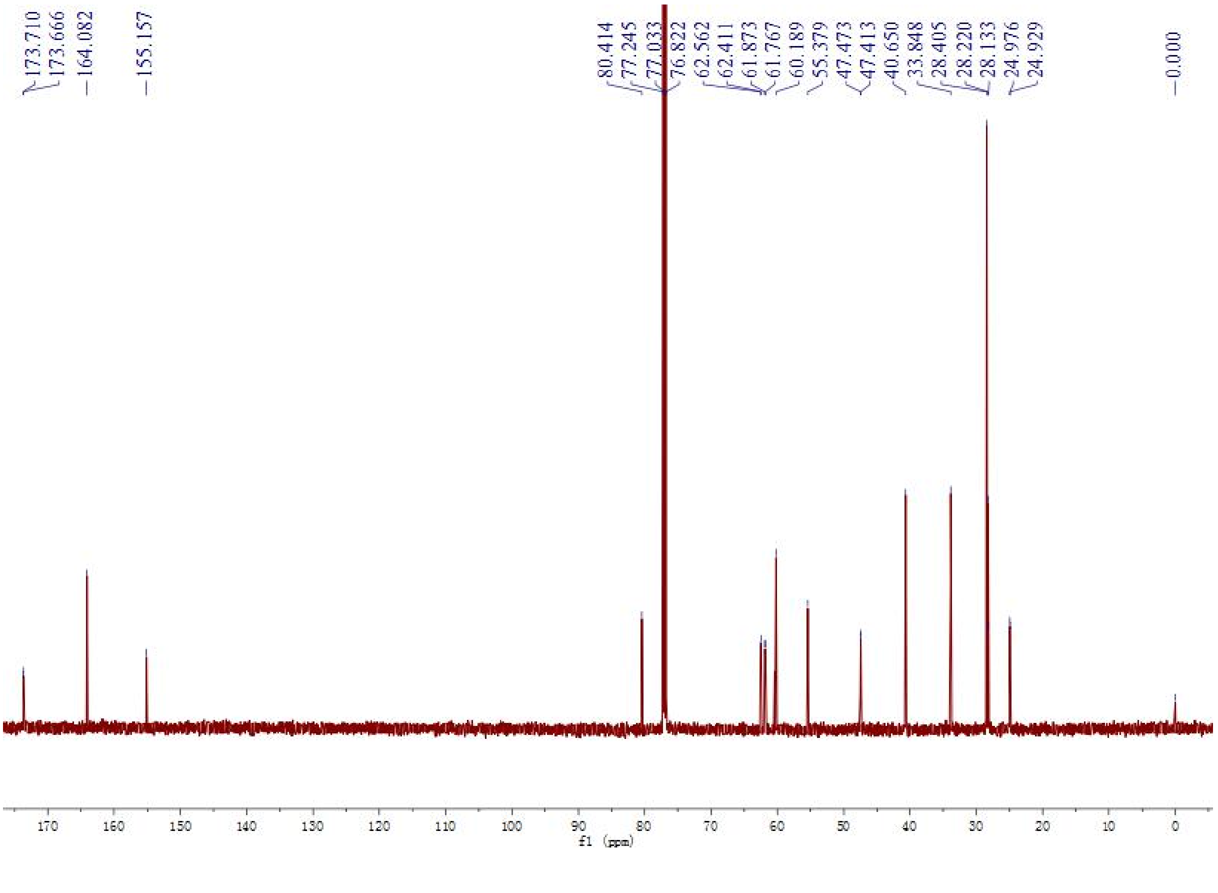

Figure S11. LR-MS of compound **2**

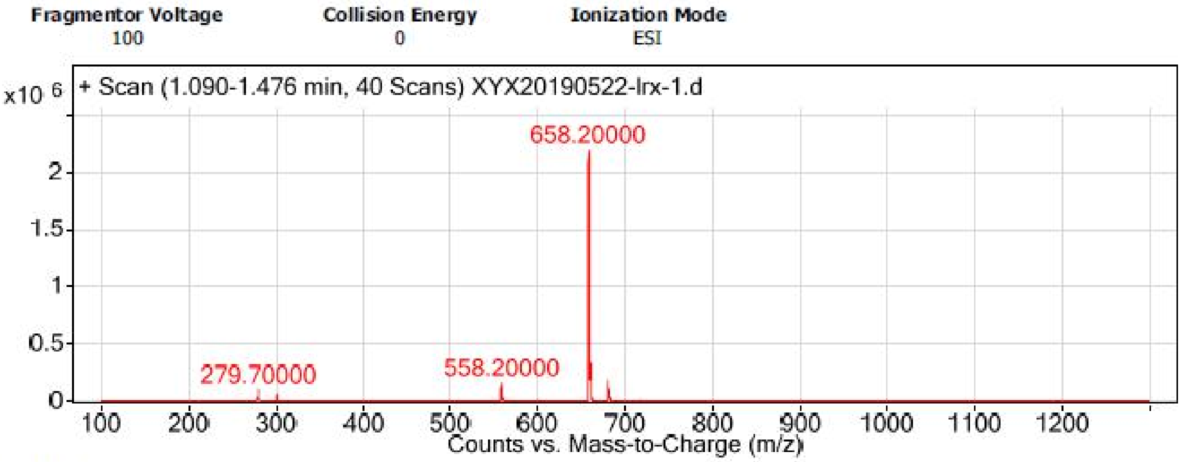

Peak List

m/z	z	Abund
279.7		100000.58
301.6		65931.89
558.2	1	159338.78
559.1	1	46905.08
658.2	1	2205089.5
659.2	1	758793.75
660.2	1	336451.38
661.2	1	88130.98
680.2	1	182844.84
681.2	1	64316.82

Figure S12. ^{1}H-NMR of compound **3**

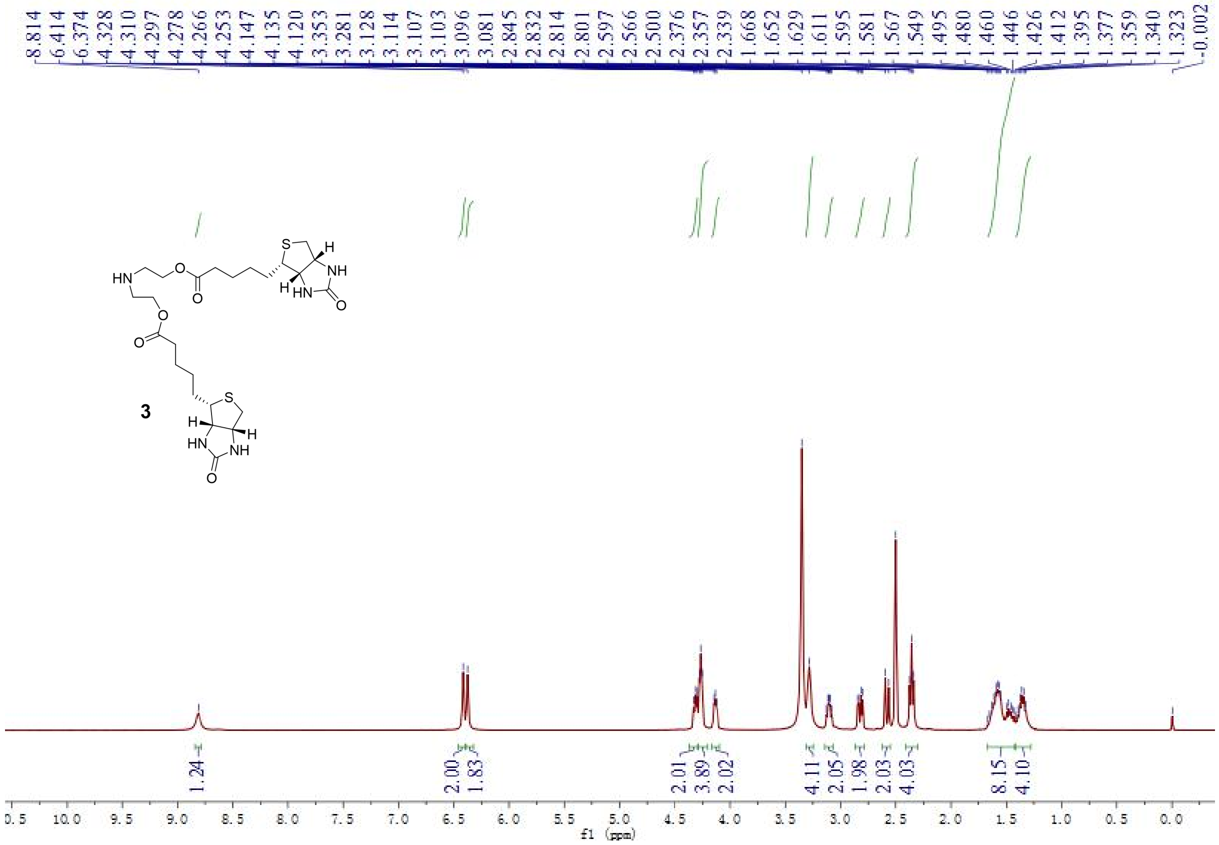

Figure S13. ^{13}C-NMR of compound **3**

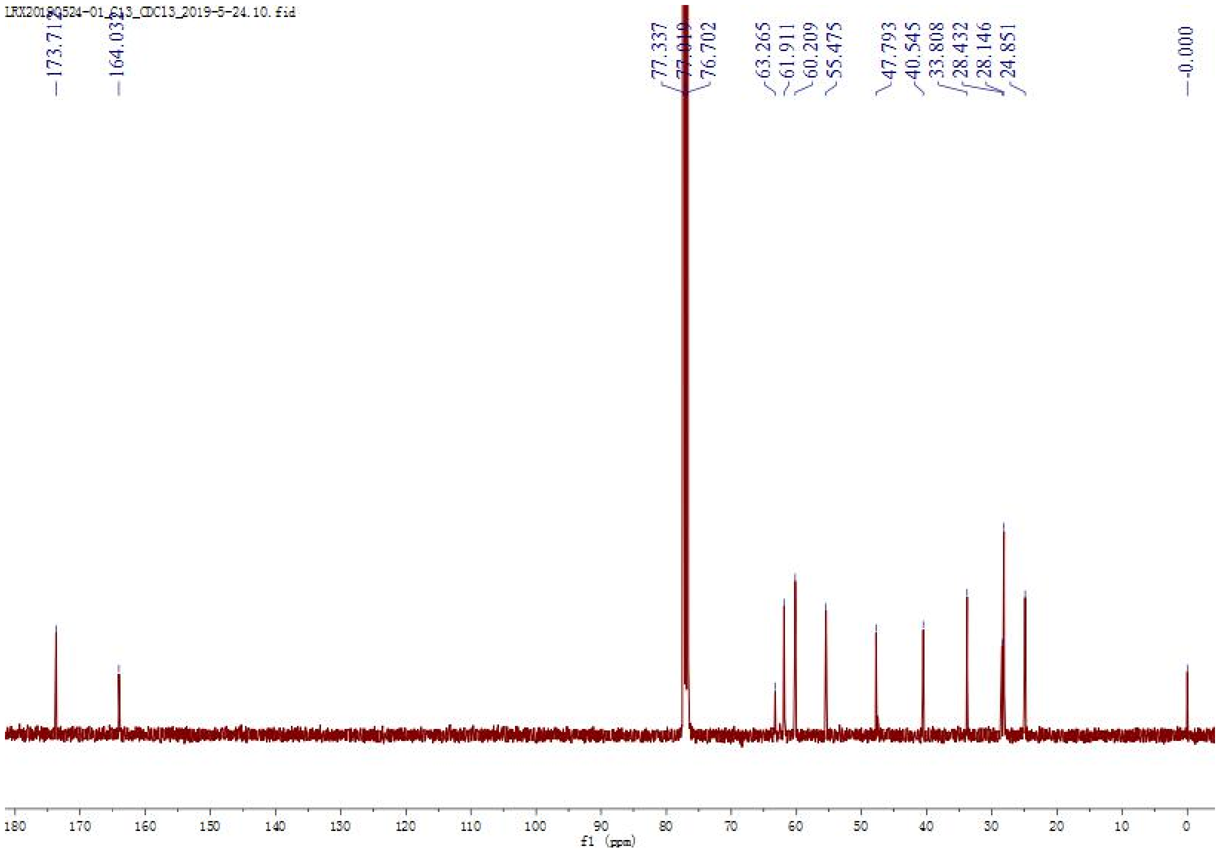

Figure S14. ^{1}H-NMR of compound **4**

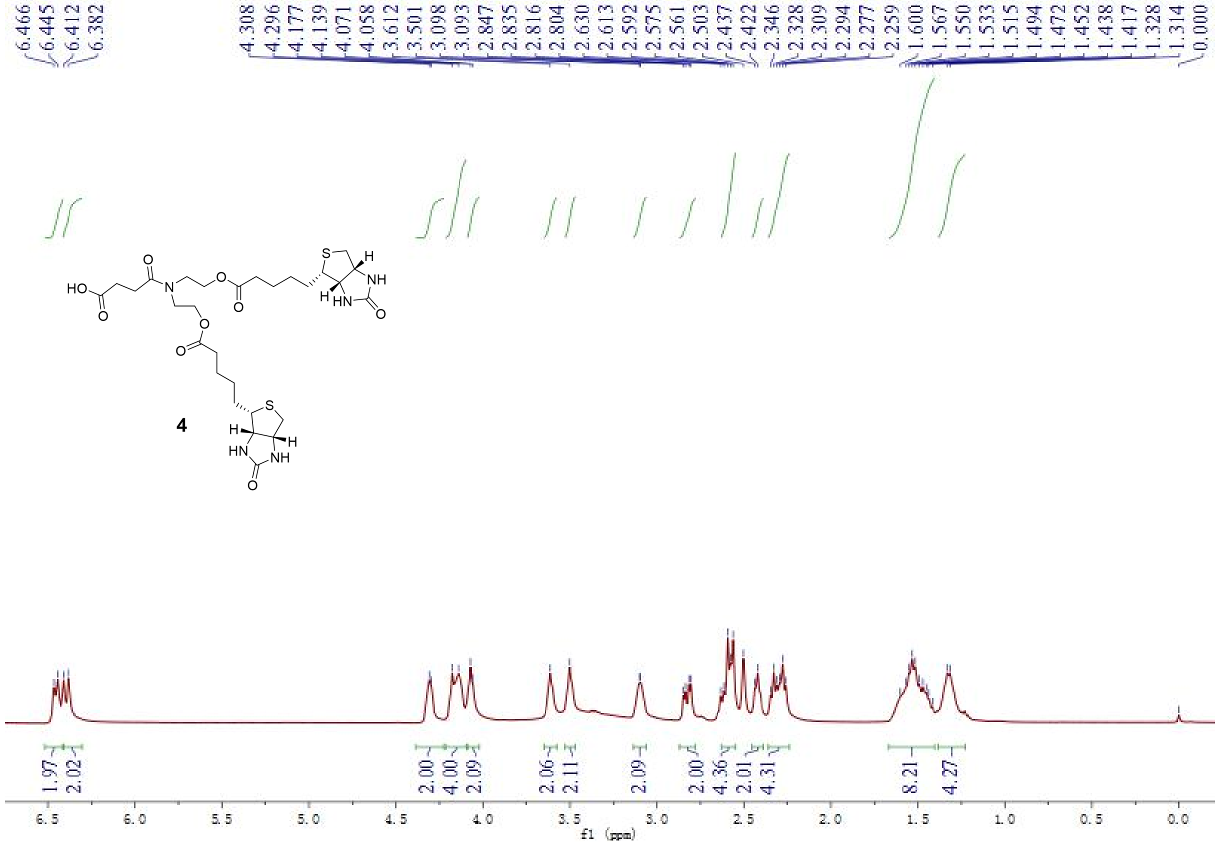

Figure S15. LR-MS of compound **4**

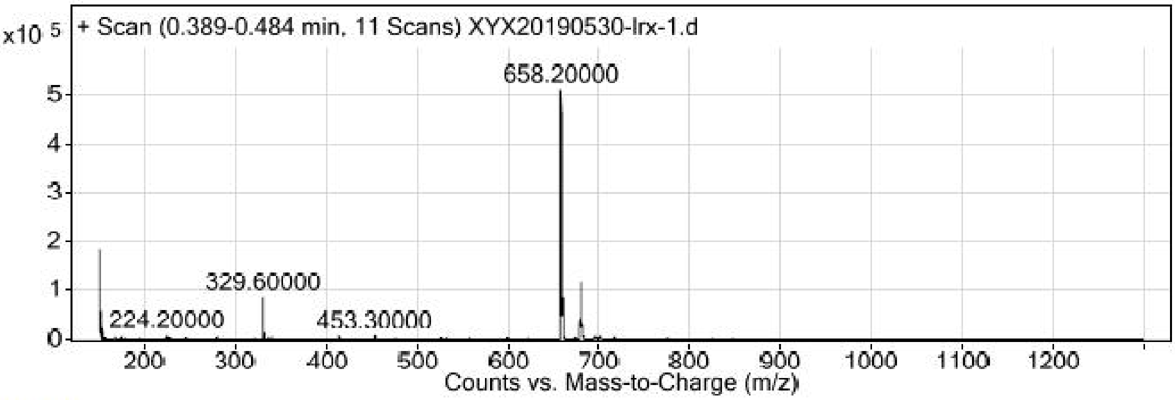

Peak List

m/z	z	Abund
151.1		14190.49
152.1		59583.64
329.6		85823.64
658.2	1	512577.19
659.2	1	181955.58
660.2	1	84332.59
661.2	1	20563.41
680.2	1	116413.38
681.1	1	37163.34
682.2	1	18638.59

Figure S16. ^{1}H-NMR of compound **6**

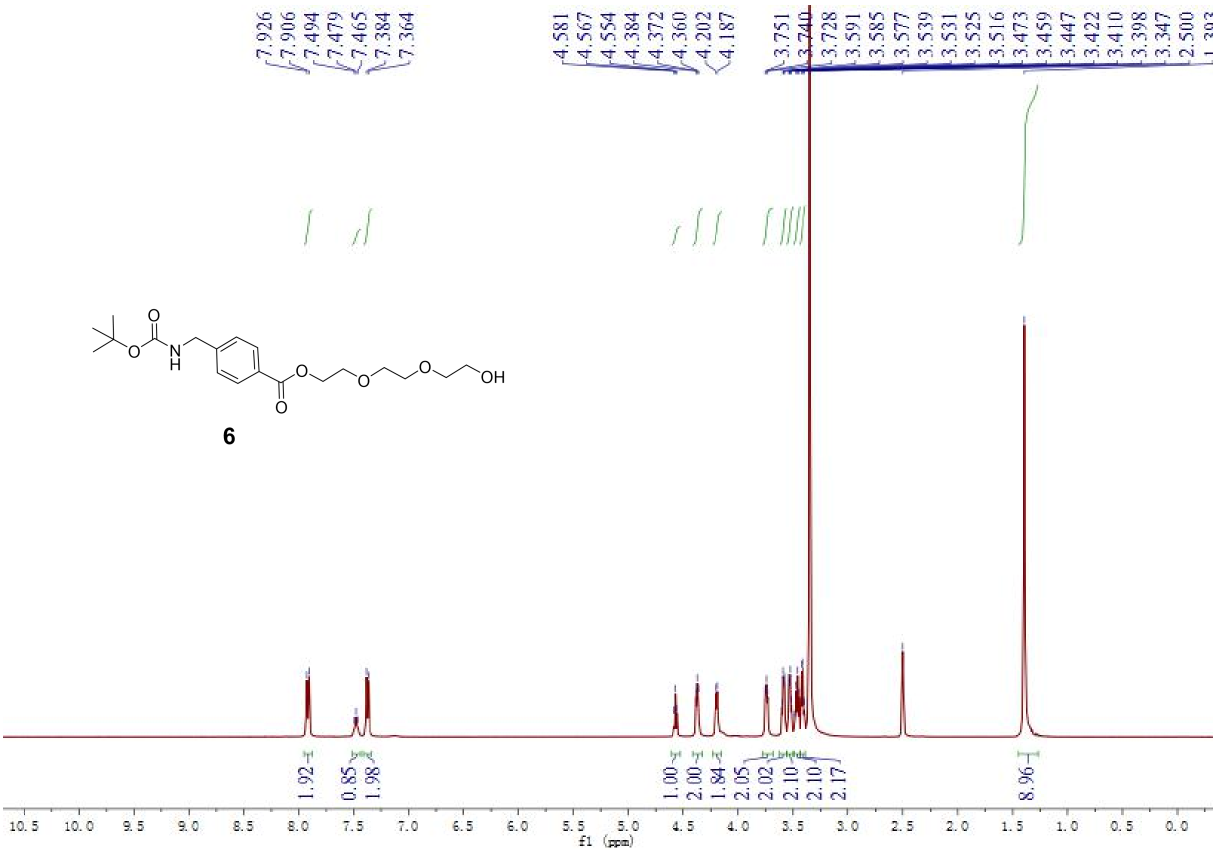

Figure S17. ^{1}H-NMR of compound **8**

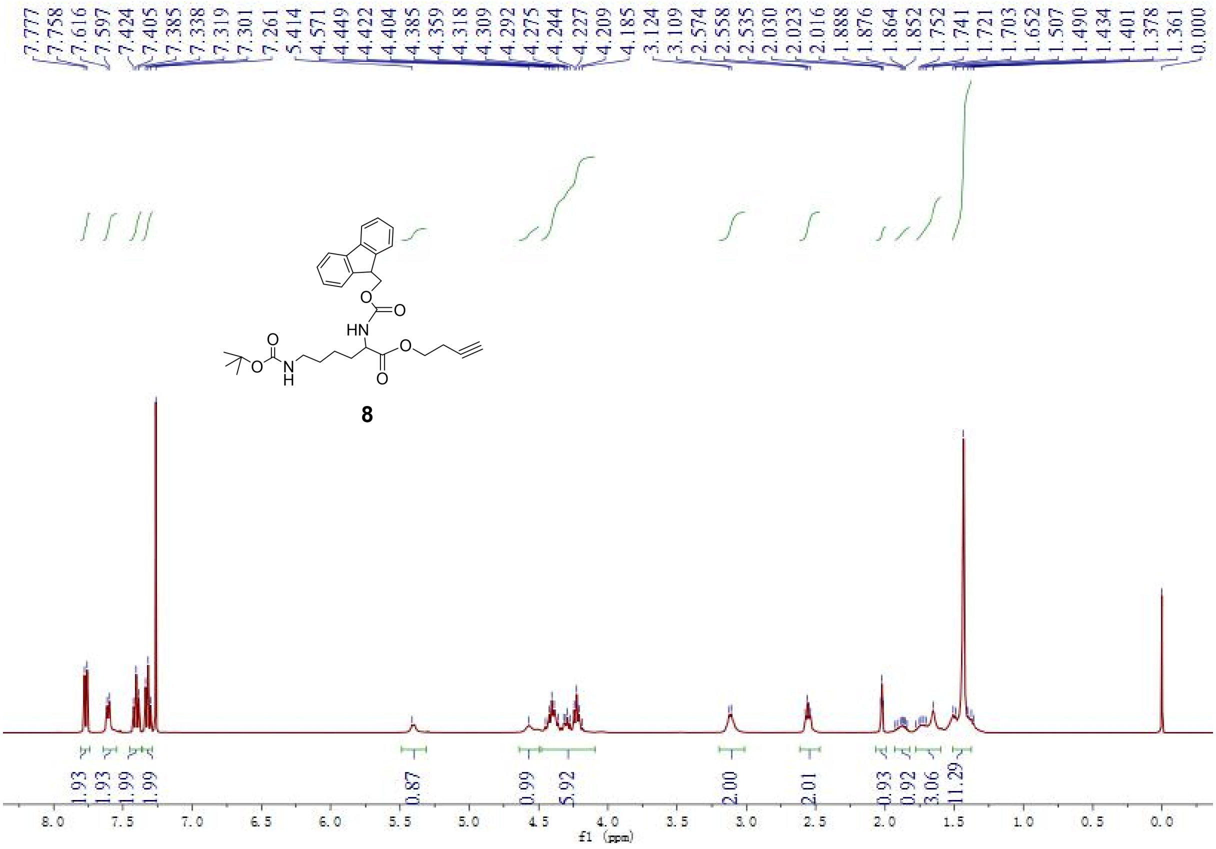

Figure S18. ^{13}C-NMR of compound **8**

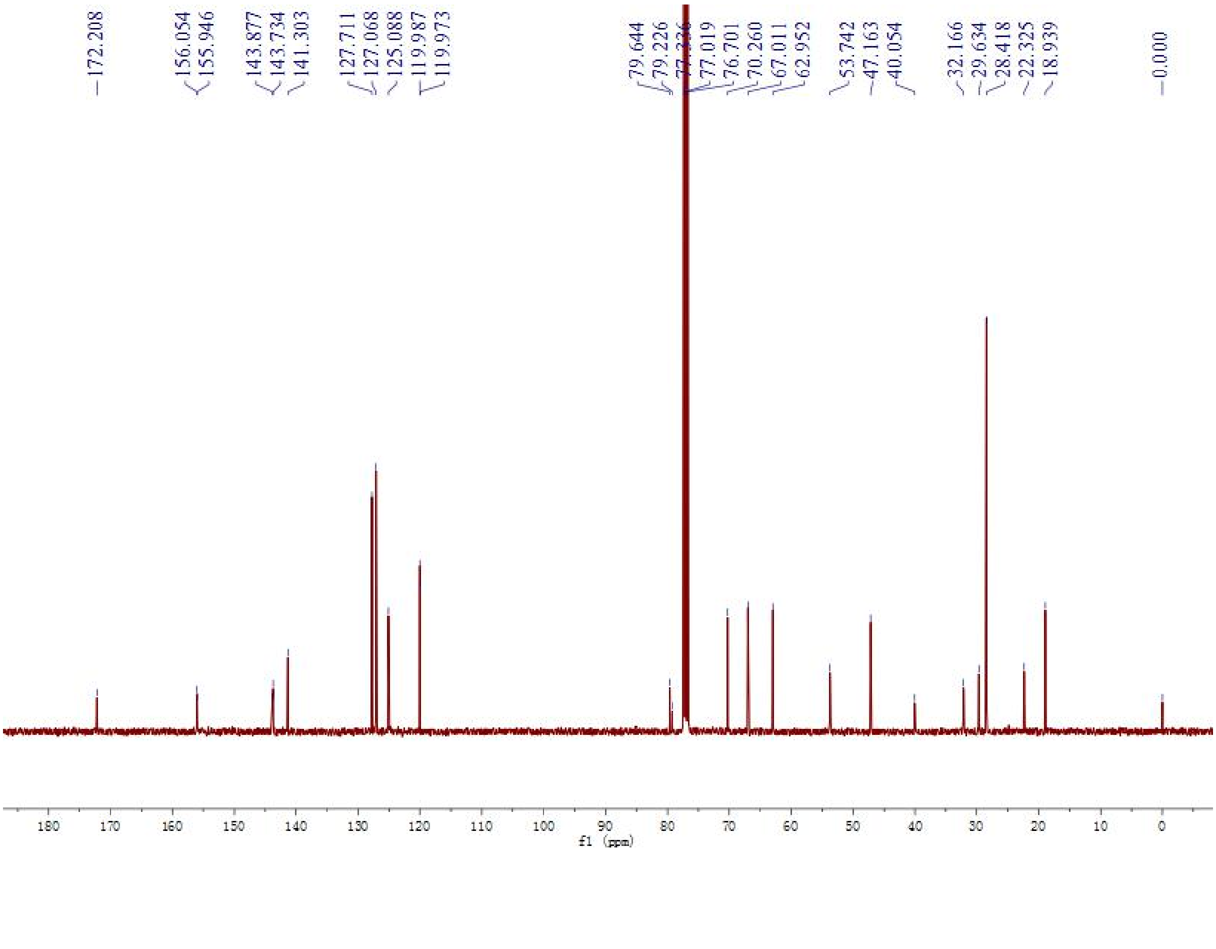

Figure S19. LR-MS of compound **8**

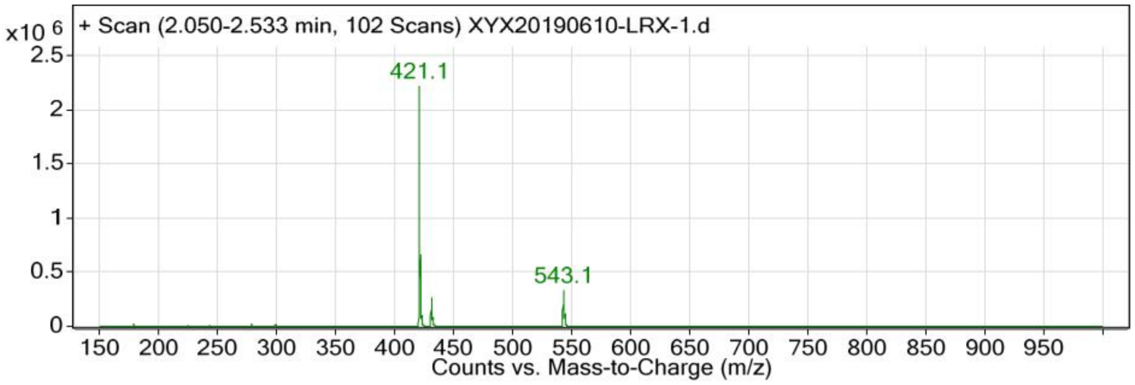

Peak List

m/z	z	Abund
421.1	1	2225940.75
422.1	1	670074.25
423.1	1	105492.27
431.3	1	262617.22
432.3	1	78380.63
543.1	1	338587.53
544.2	1	113391.56
545.1	1	22344.17

Figure S20. ^{1}H-NMR of compound **9**

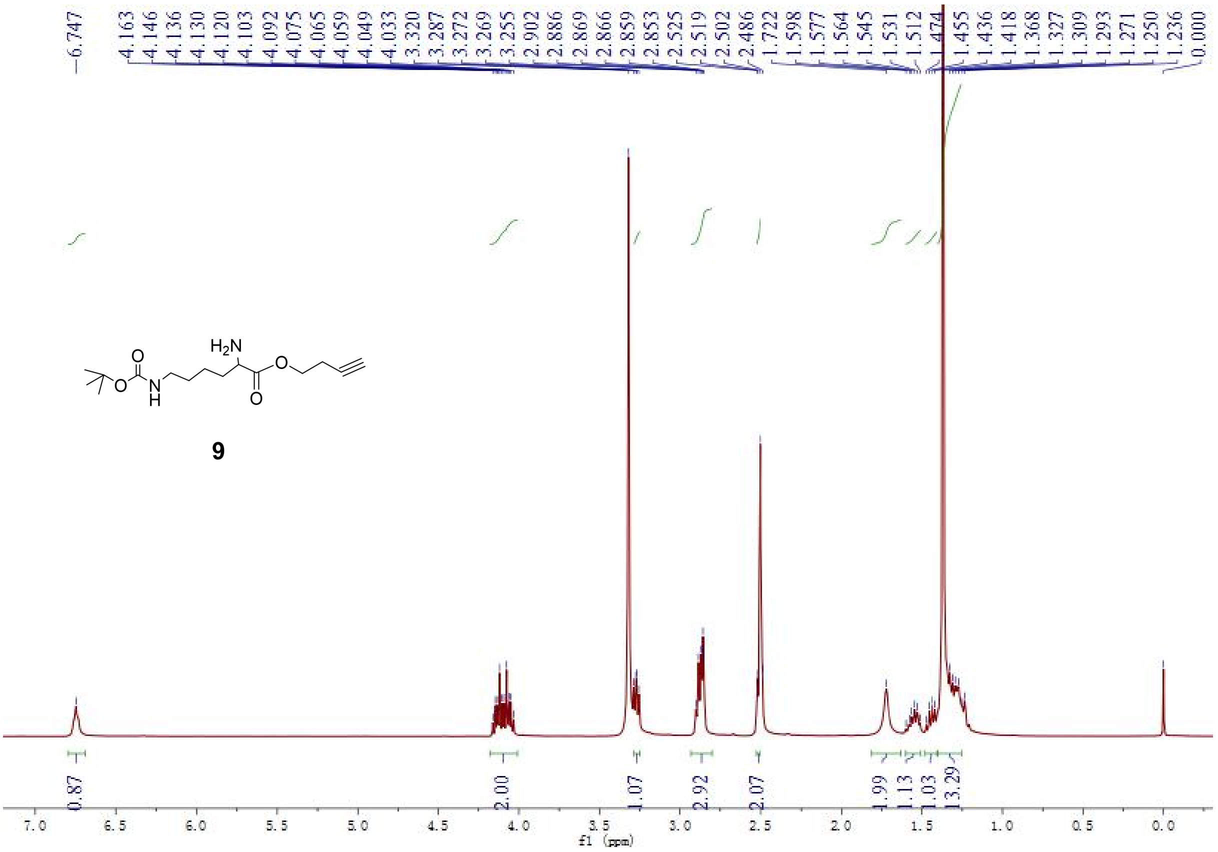

Figure S21. ^{13}C-NMR of compound **9**

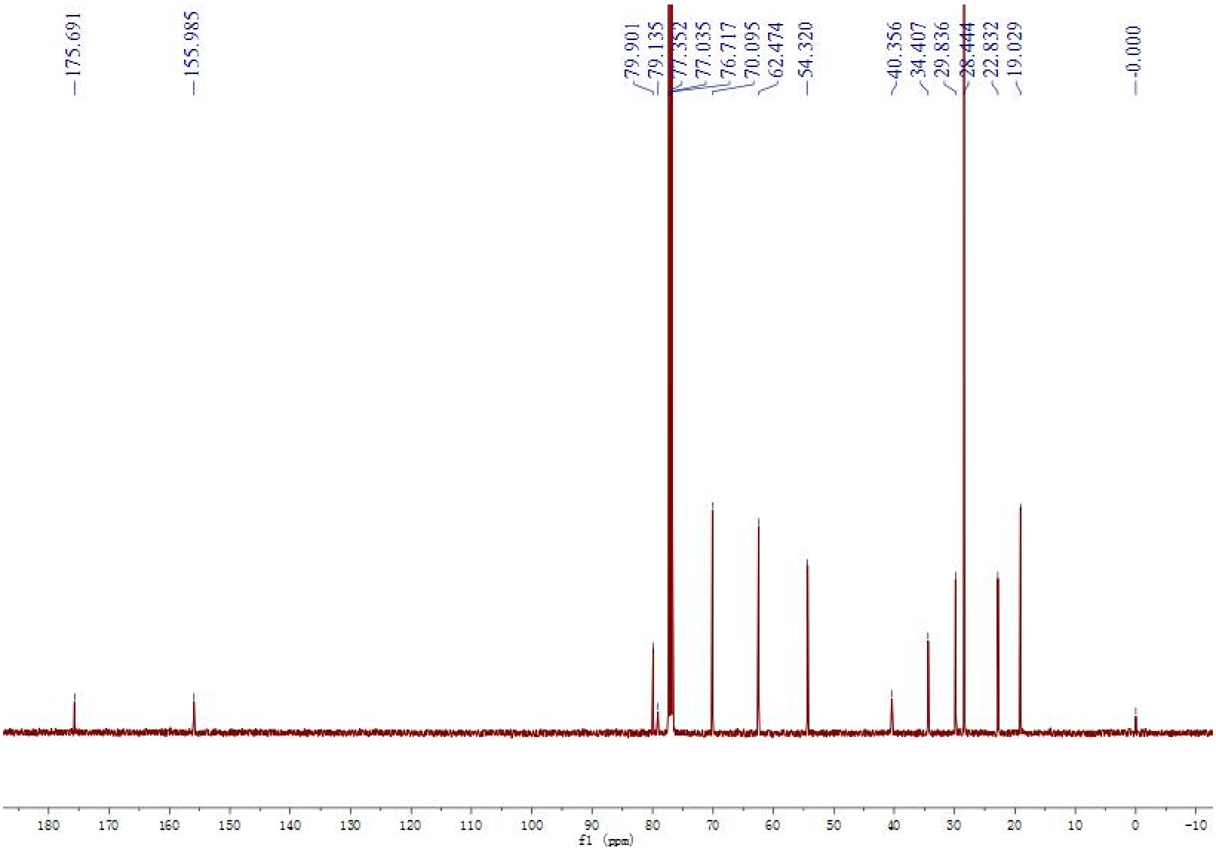

Figure S22. LR-MS of compound **9**

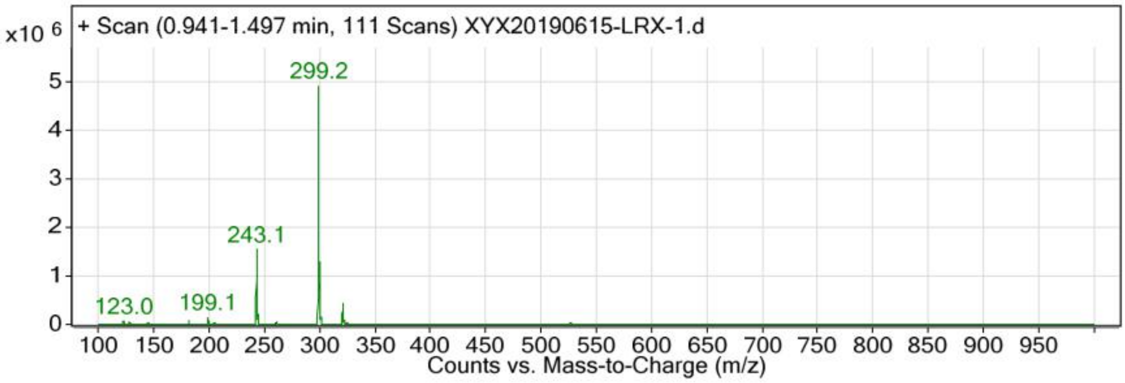

Peak List

m/z	z	Abund
123		72831.79
182.1		85045.86
199.1		146494.2
243.1	1	1542899.75
244.1	1	195290.11
299.2	1	4904974.5
300.2	1	1298051
301.2	1	158265.67
321.1	1	442616.47
322.1	1	75269.27

Figure S23. ^{1}H-NMR of compound **10**

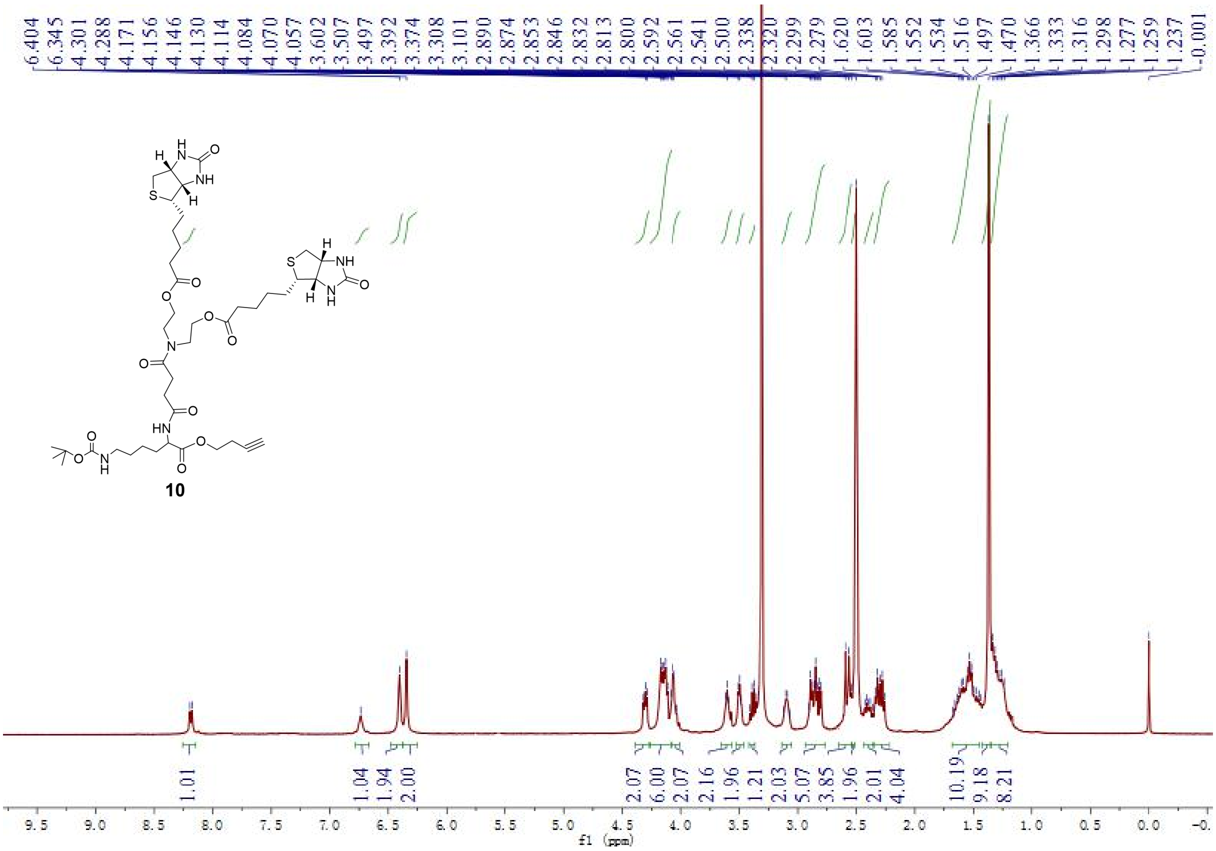

Figure S24. LR-MS of compound **10**

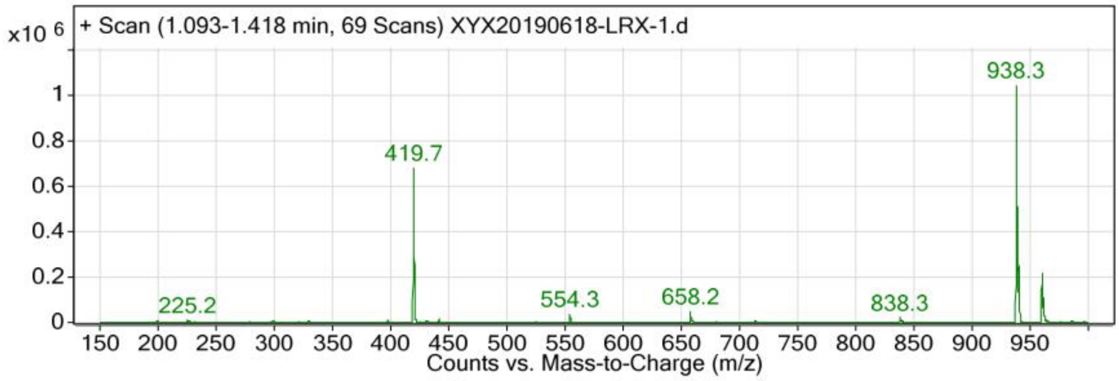

Peak List

m/z	z	Abund
419.7		682705.75
554.3		35297.46
658.2	1	48296.85
938.3	1	1044226.06
939.3	1	512137.16
940.3	1	241601.31
941.3	1	78373.3
960.3	1	218829.7
961.3	1	111051.05
962.3	1	53541.01

Figure S25. ^{1}H-NMR of compound **11**

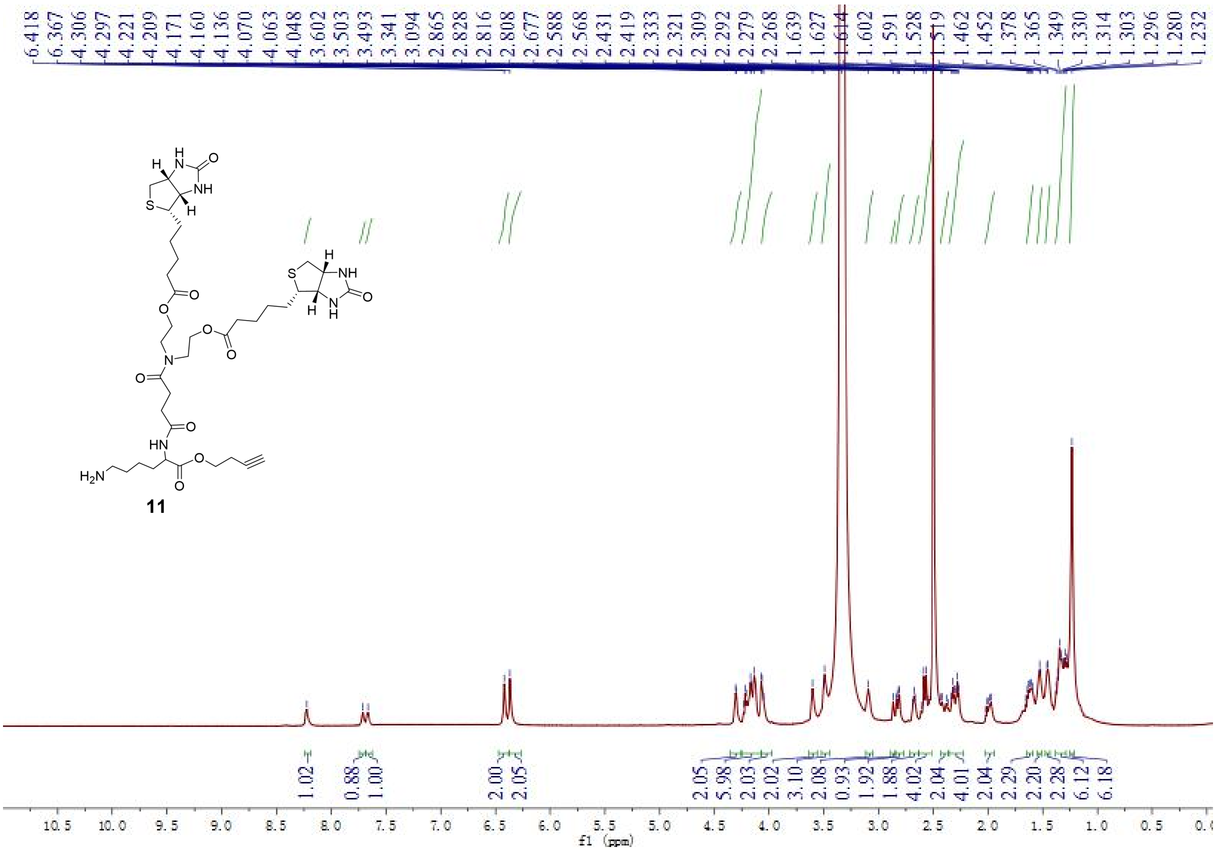

Figure S26. LR-MS of compound **11**

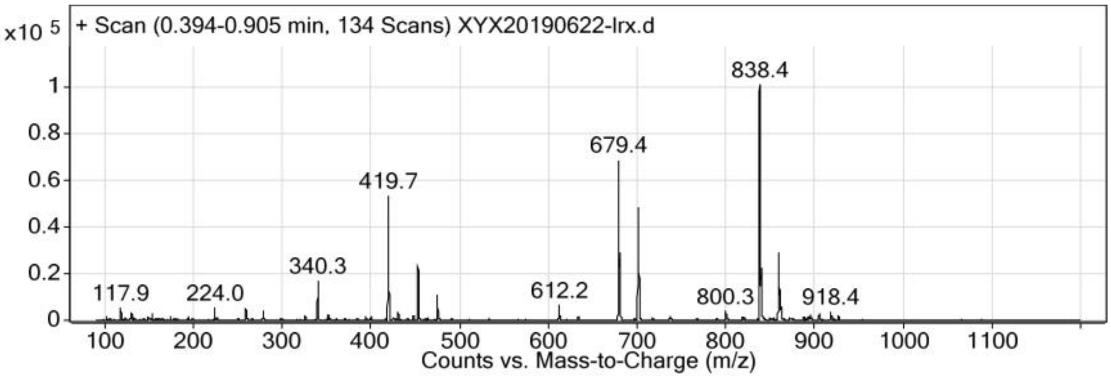

Peak List

m/z	z	Abund
419.7		53441.05
453.3		22896.9
679.4	1	68915.17
680.4	1	28992.39
701.4	1	48483.71
702.4	1	20859.02
838.4	1	101512.99
839.3	1	48560.41
840.3	1	22298.57
860.3	1	28946.87

Figure S27. ^{1}H-NMR of compound **12**

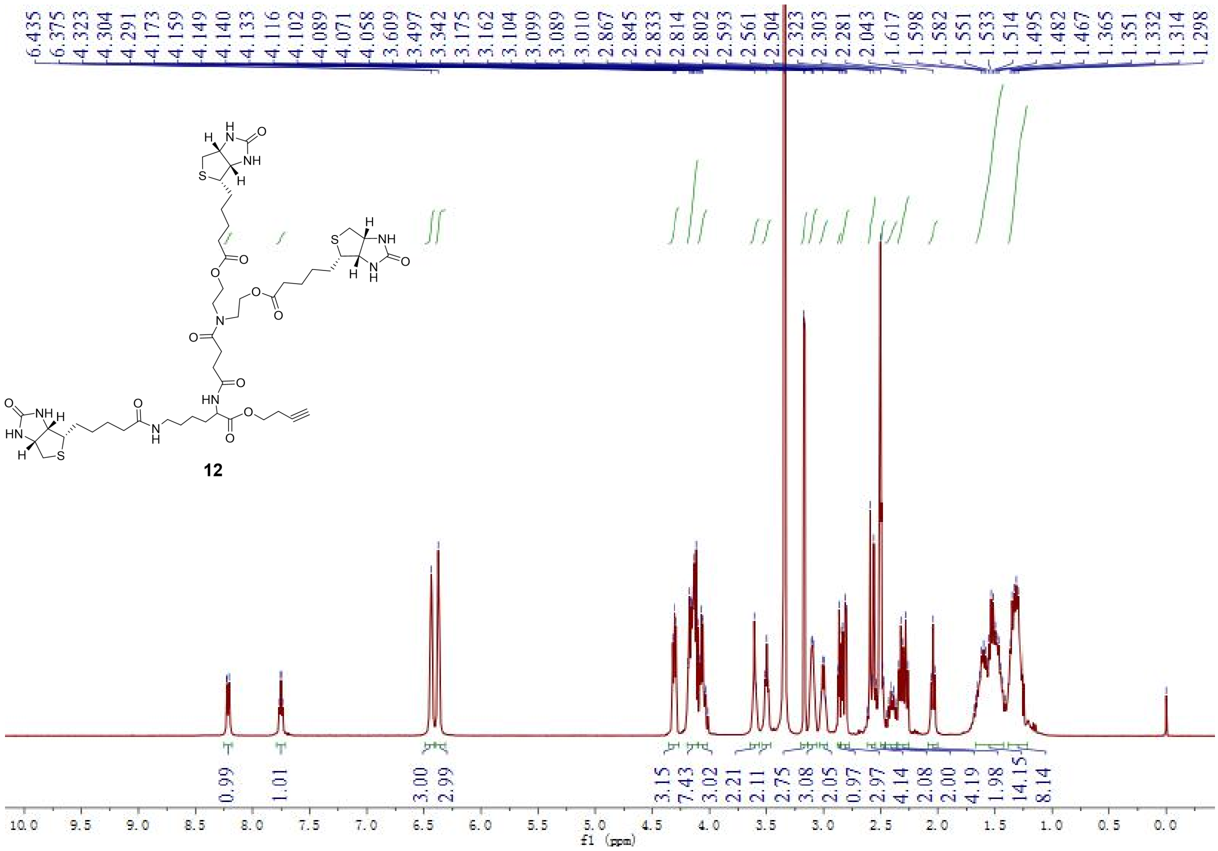

Figure S28. LR-MS of compound **12**

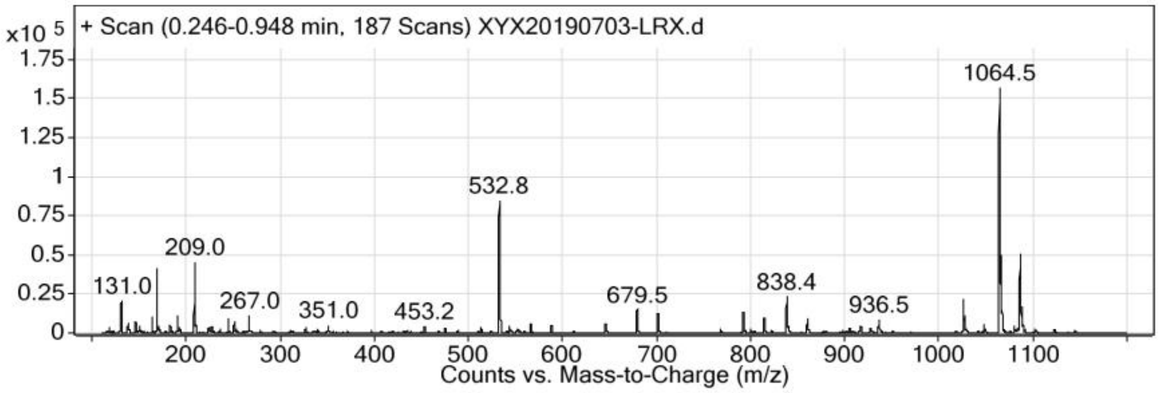

Peak List

m/z	z	Abund
169	1	41305.77
209	1	45059.38
532.8		84788.55
838.4	1	23079.39
1026.5	1	21716.68
1064.5	1	157180.53
1065.5	1	91468
1066.5	1	50182.81
1086.5	1	51115.81
1087.5	1	31133.76

Figure S29. ^{1}H-NMR of compound **14**

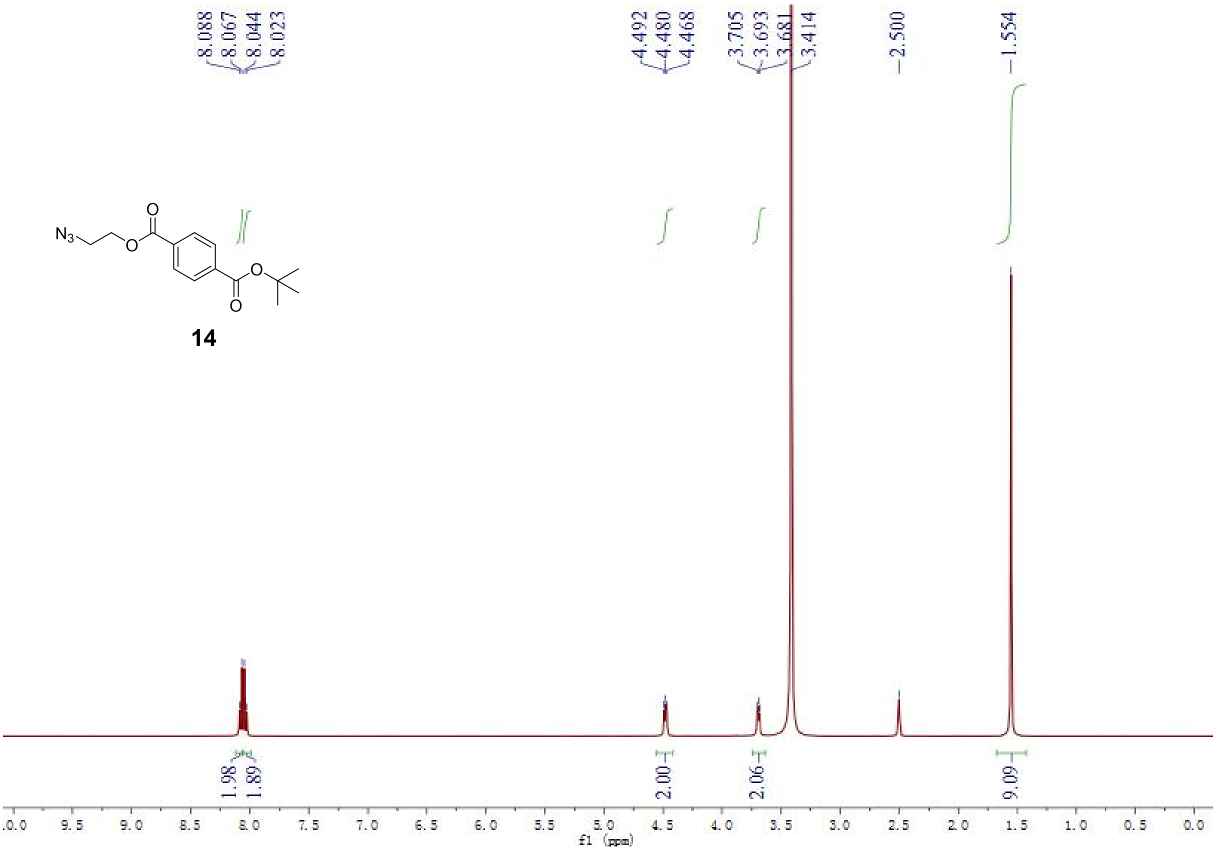

Figure S30. ^{1}H-NMR of compound **15**

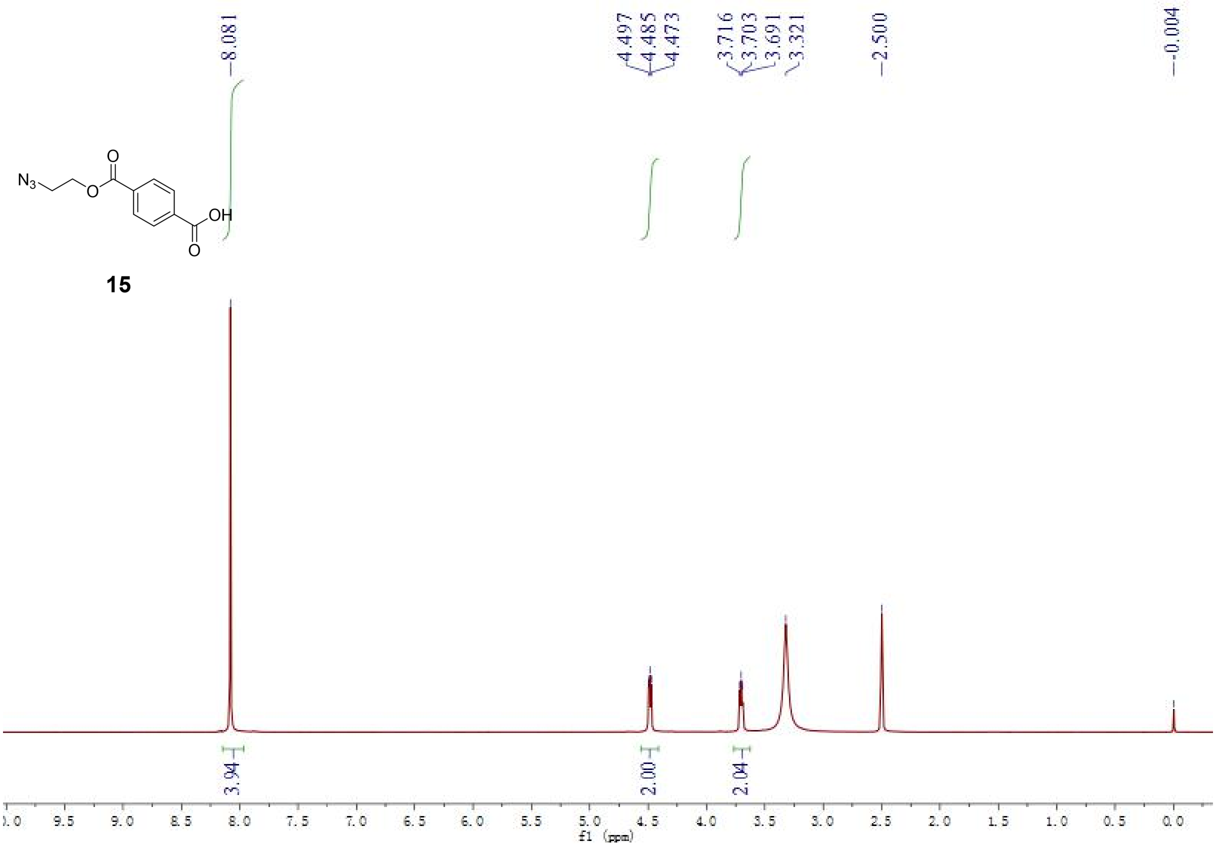

Figure S31. ^{13}C-NMR of compound **15**

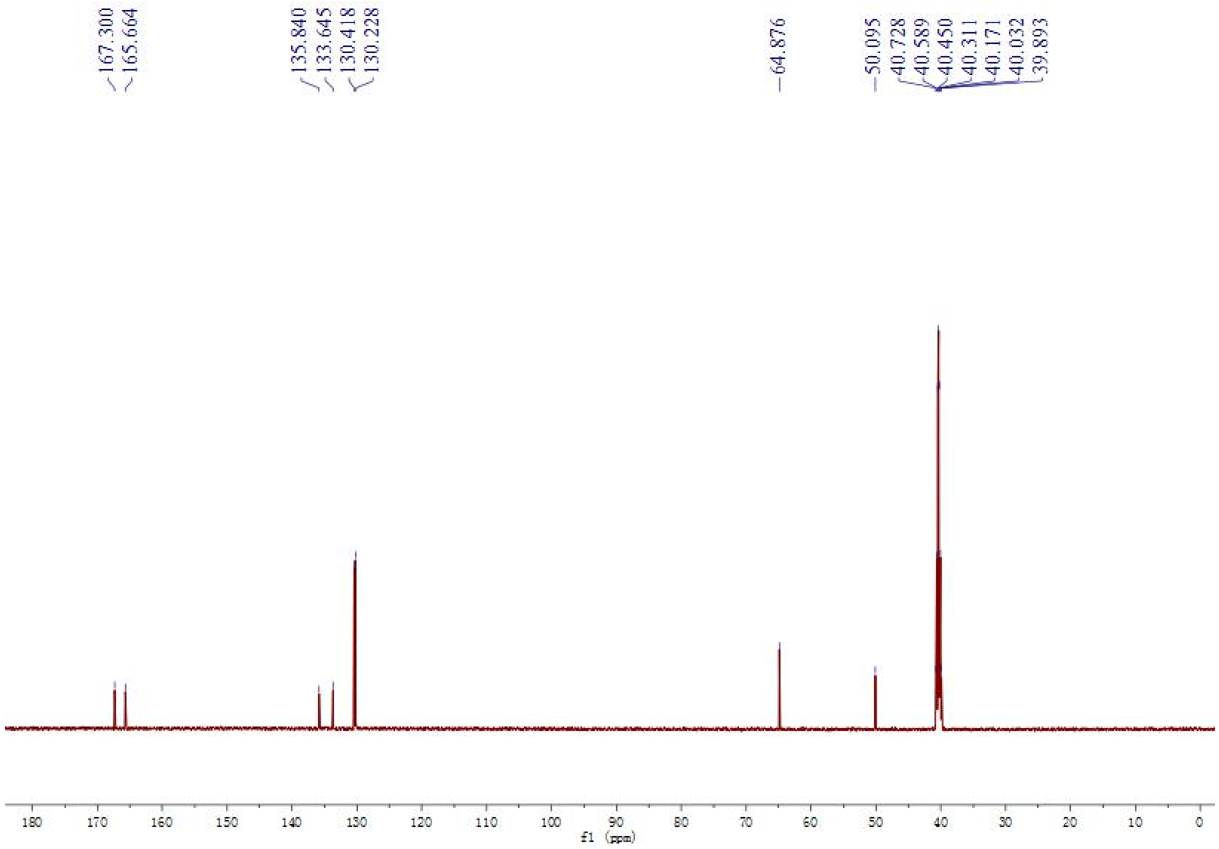

Figure S32. ^{1}H-NMR of compound **16**

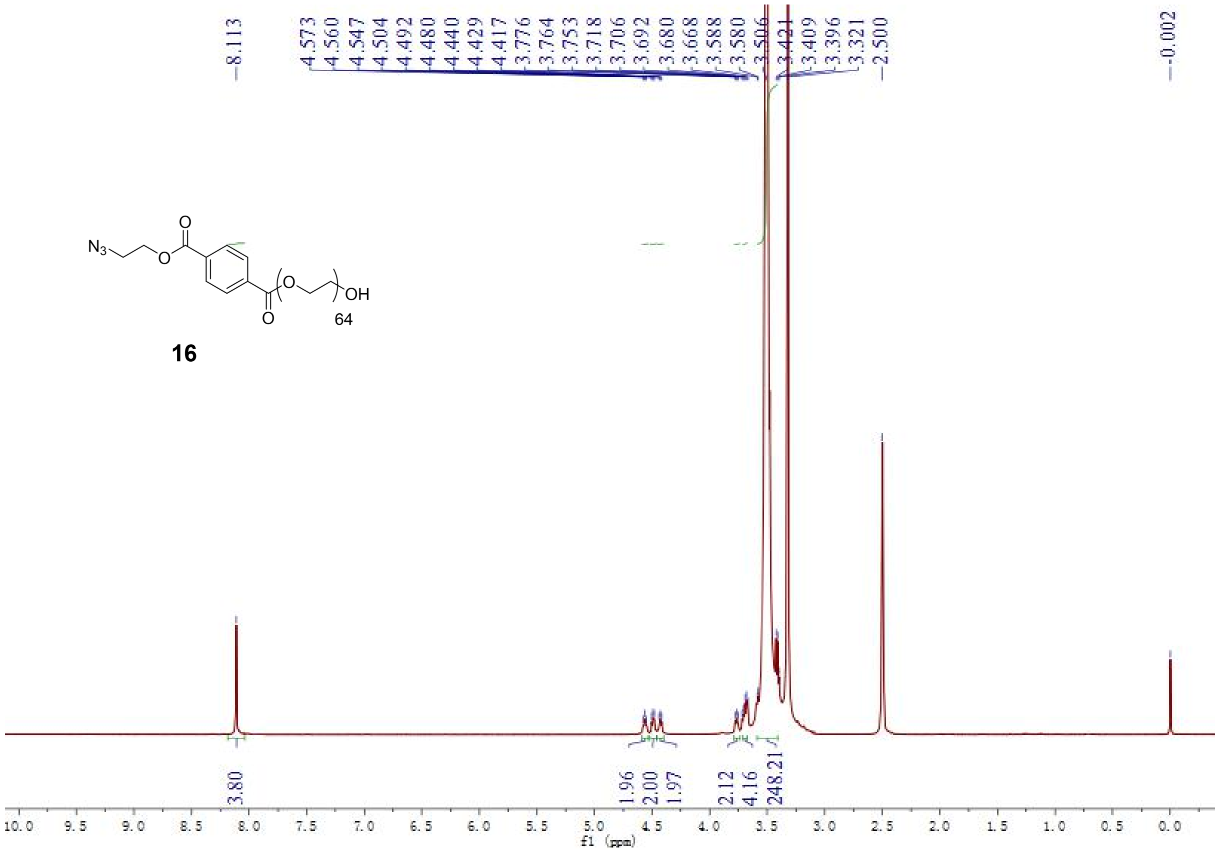

Figure S33. ^{1}H-NMR of compound **17**

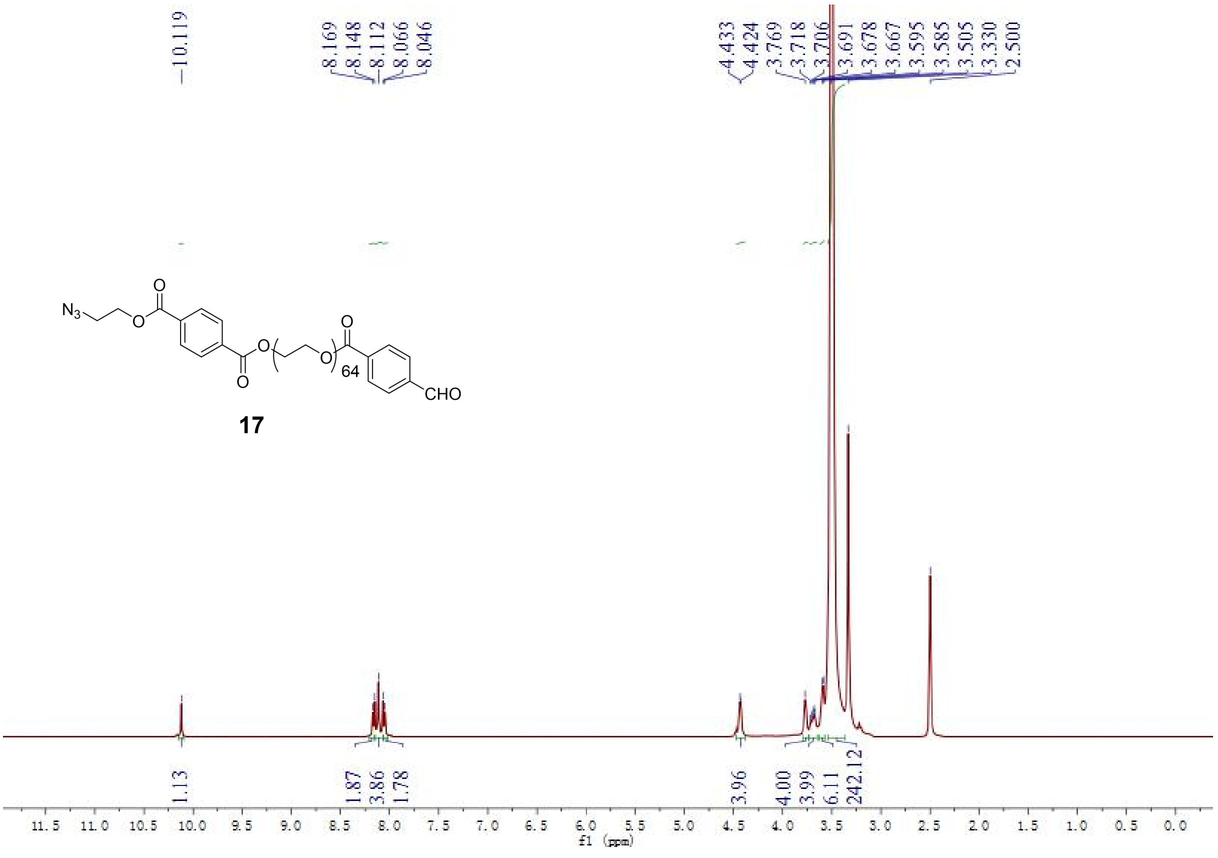

Figure S34. ^{1}H-NMR of compound **18**

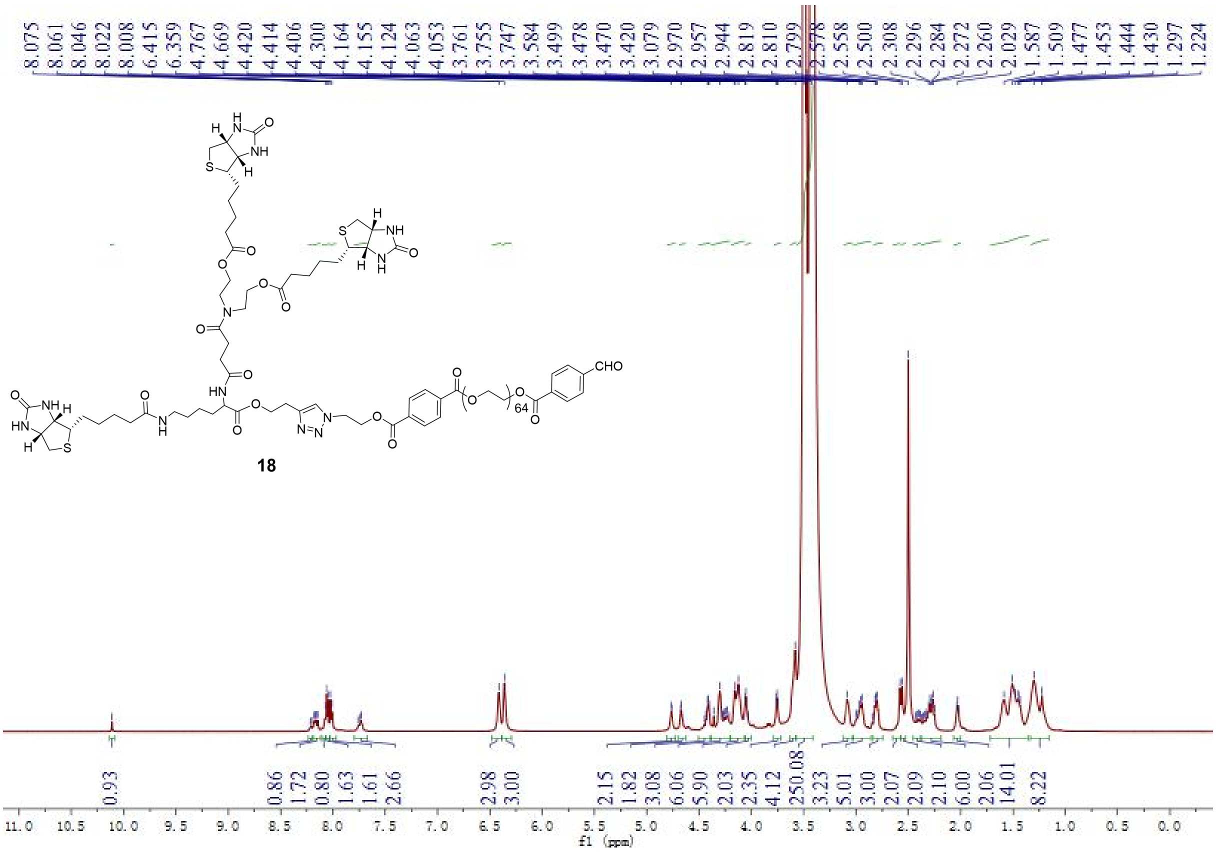

Figure S35. ^{1}H-NMR of compound **20**

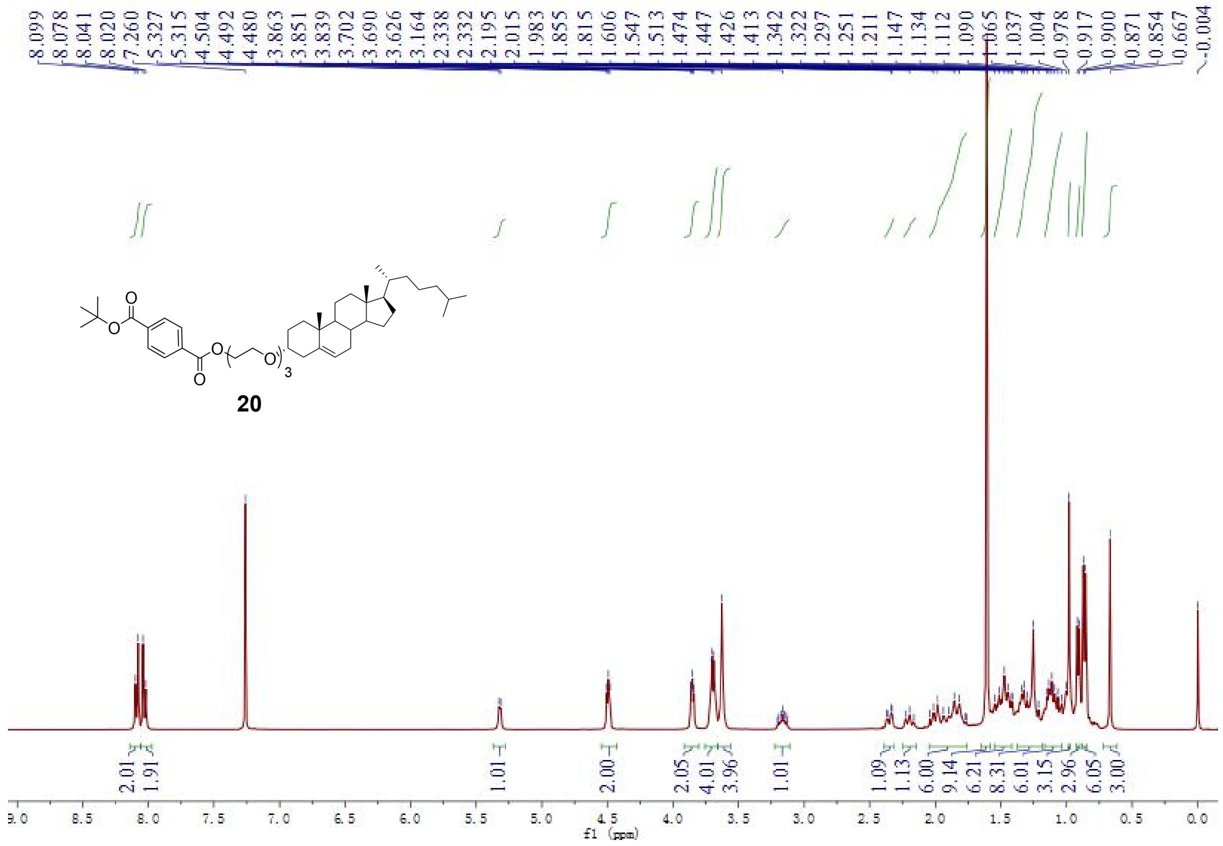

Figure S36. ^{1}H-NMR of compound **21**

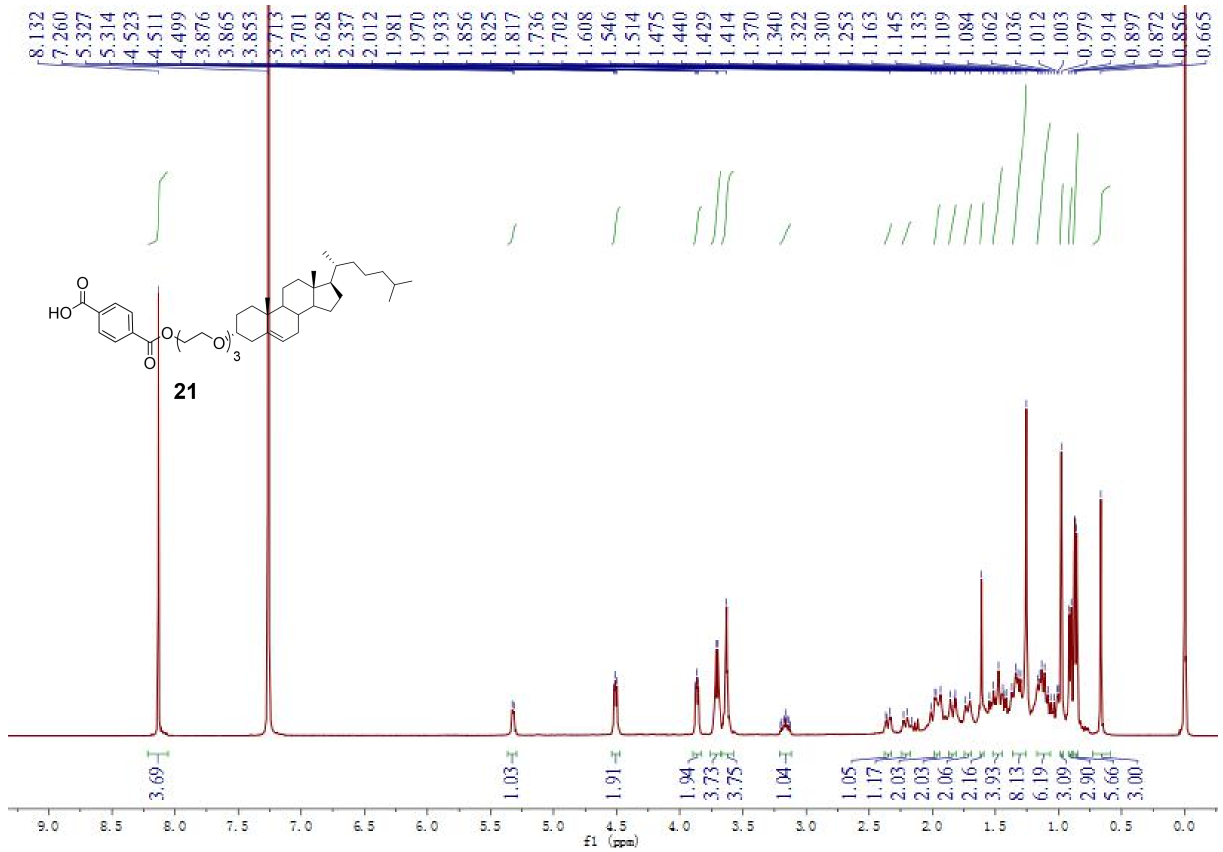

Figure S37. ^{1}H-NMR of compound **22**

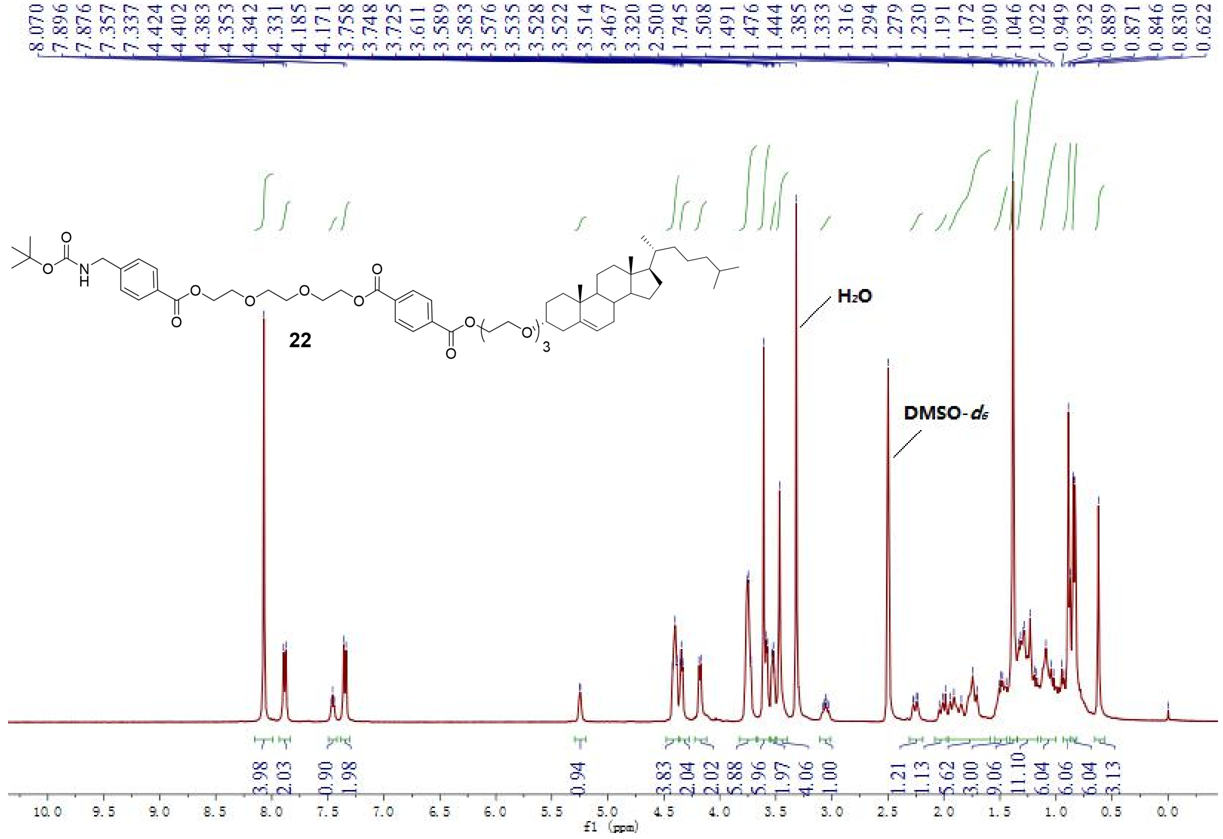

Figure S38. ^{1}H-NMR of compound **23**

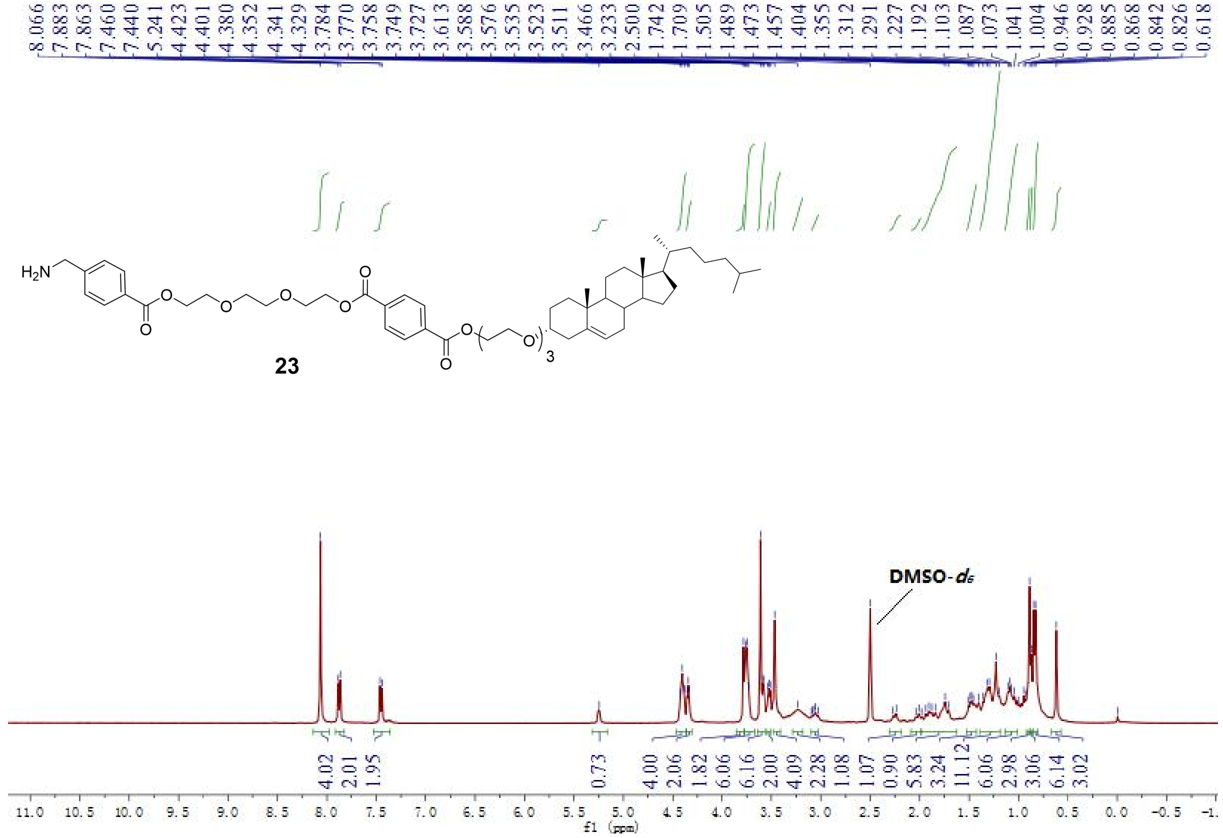

Figure S39. ^{1}H-NMR of ligand **Bio$_3$-PEG-BIm**

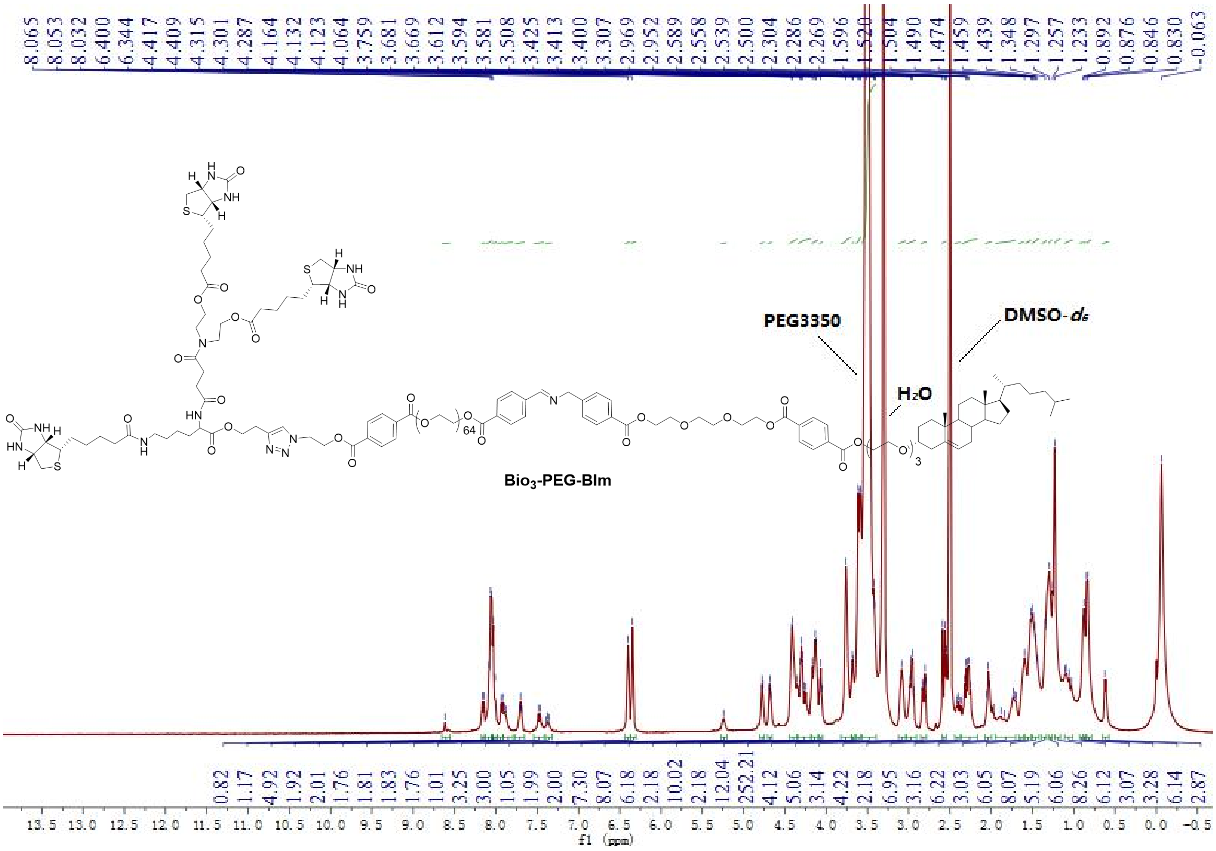

Figure S40. HR-MS of ligand **Bio$_3$-PEG-BIm**

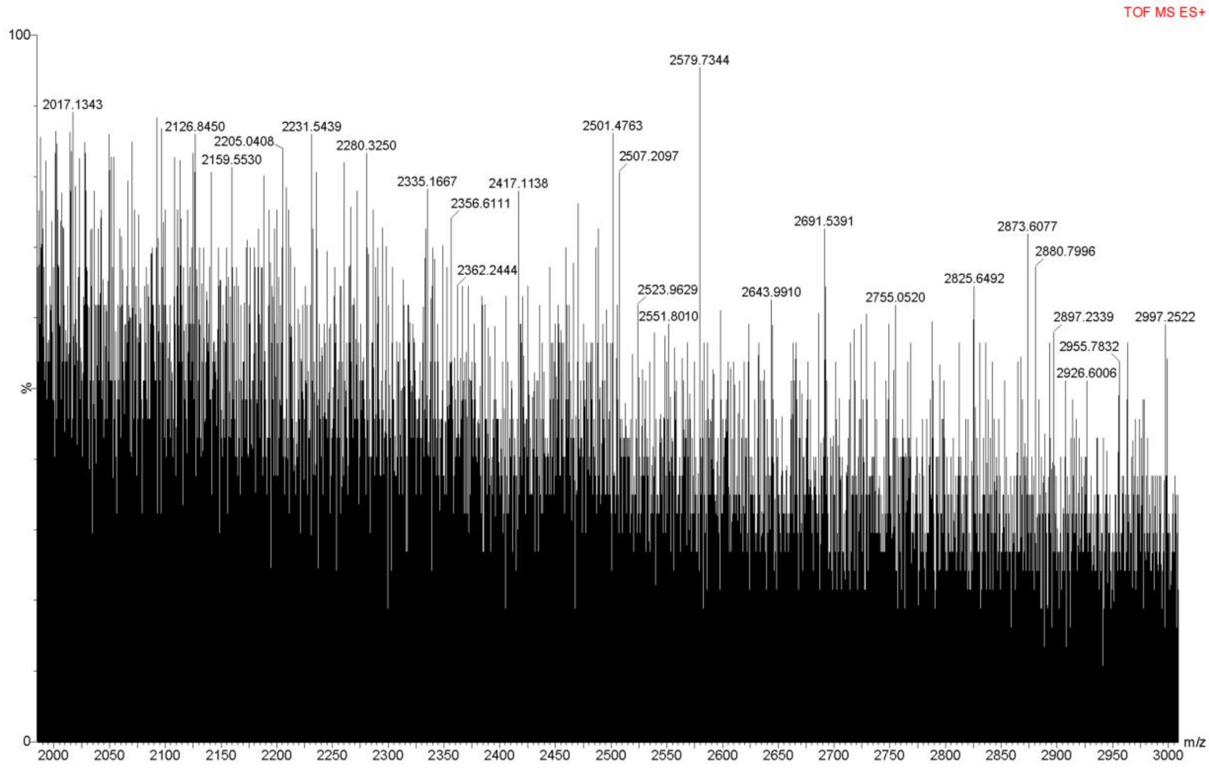

Figure S41. ^{1}H-NMR of compound **25**

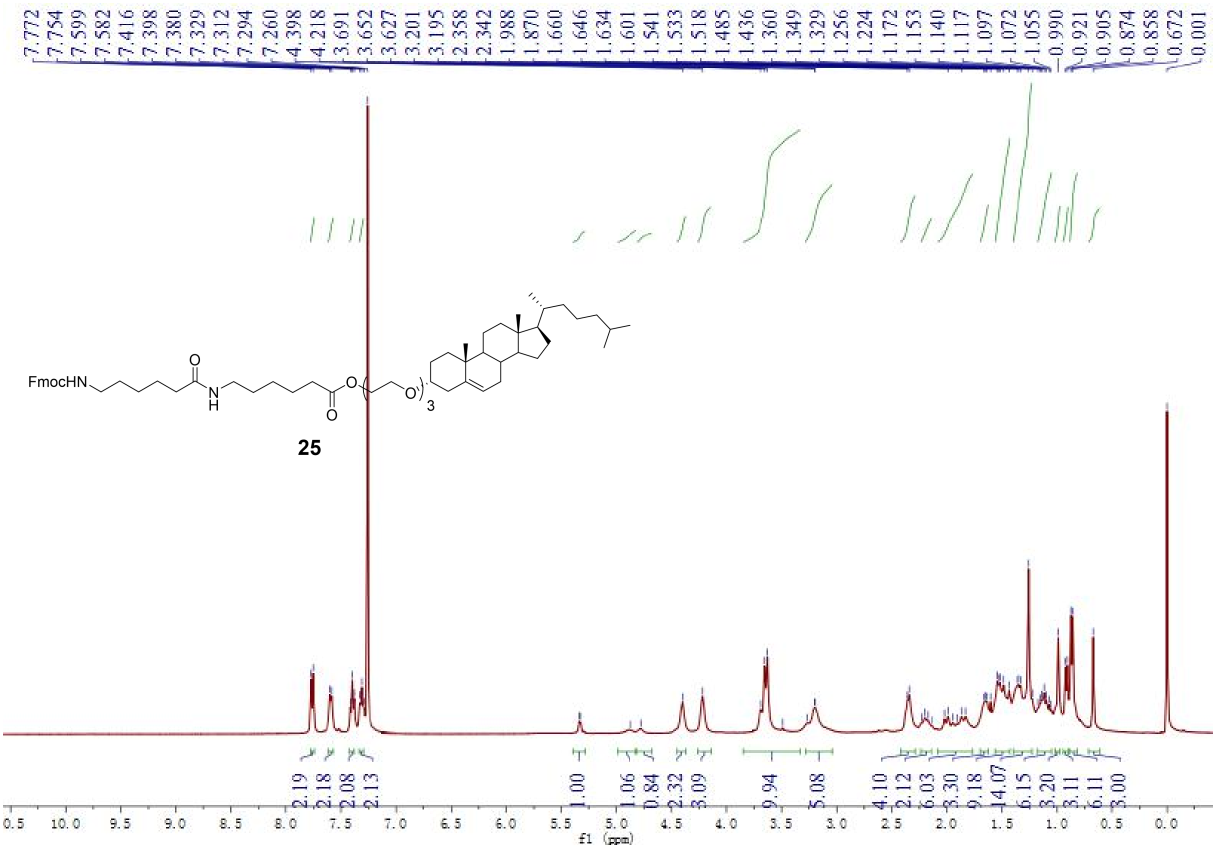

Figure S42. ^{13}C-NMR of compound **25**

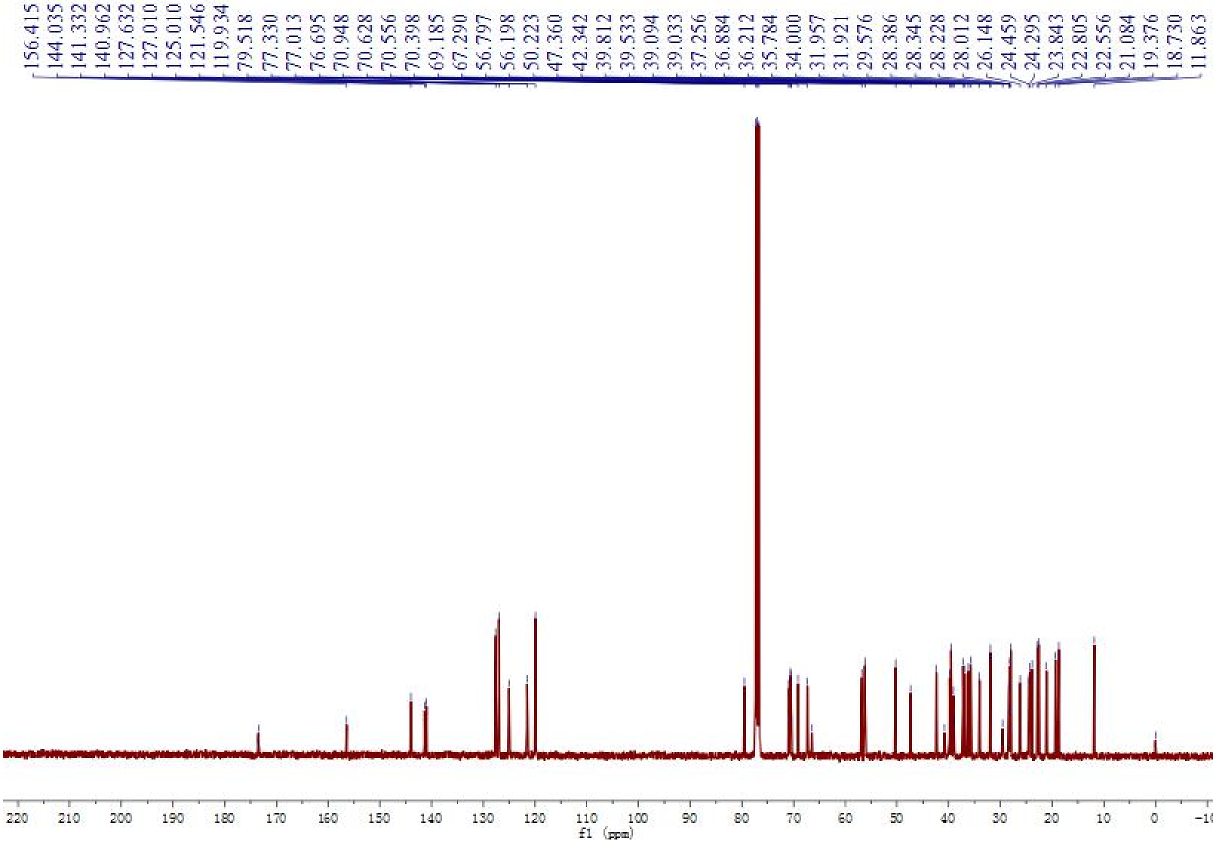

Figure S43. ^{1}H-NMR of ligand **Bio$_3$-PEG-AIm**

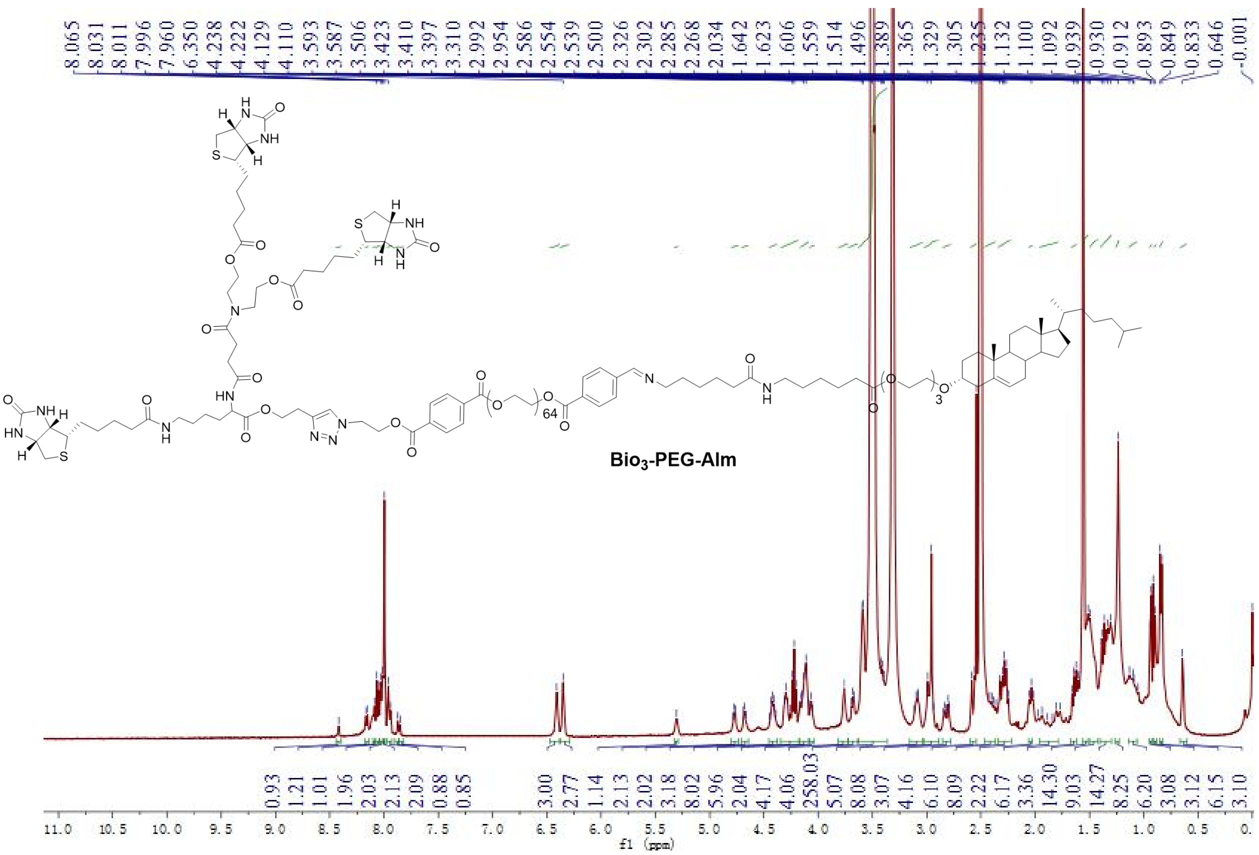

Figure S44. ^{1}H-NMR of compound **28**

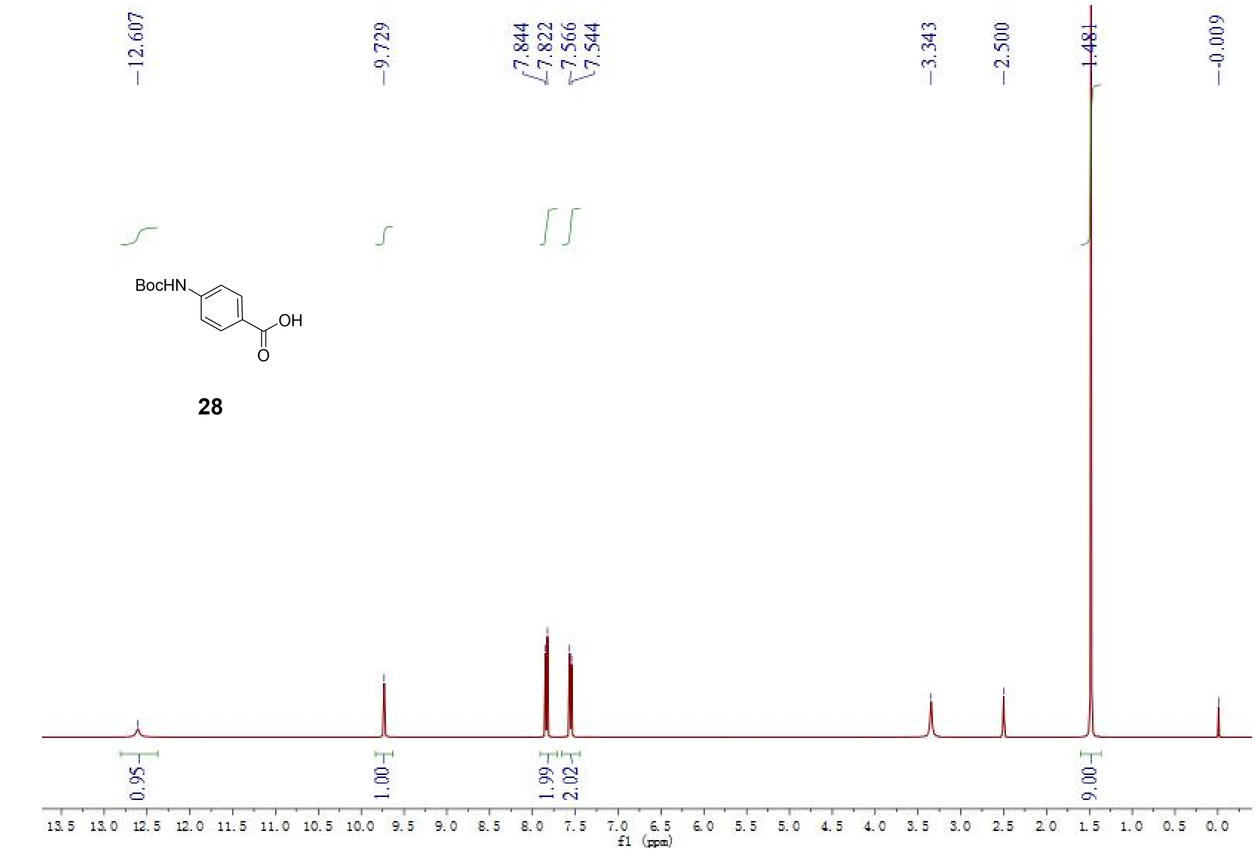

Figure S45. ^{1}H-NMR of compound **29**

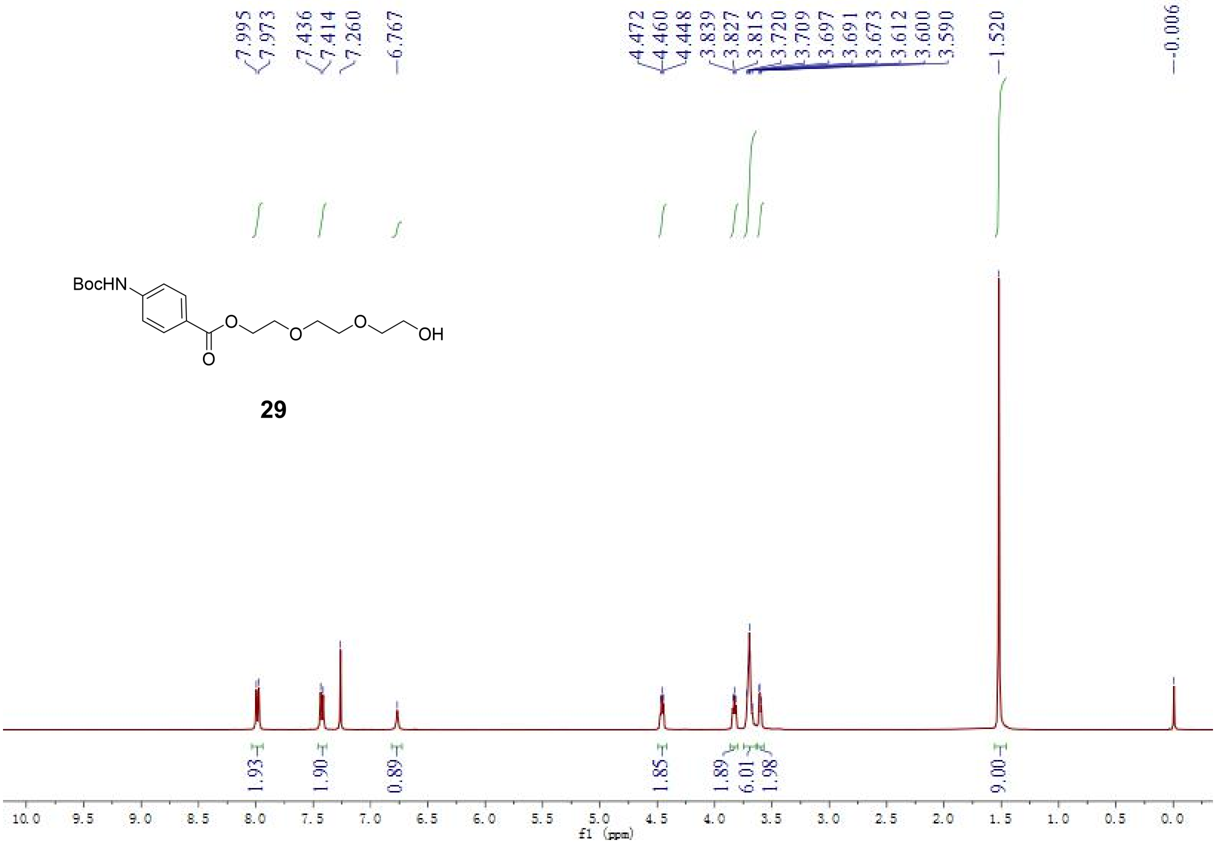

Figure S46. ^{1}H-NMR of compound **30**

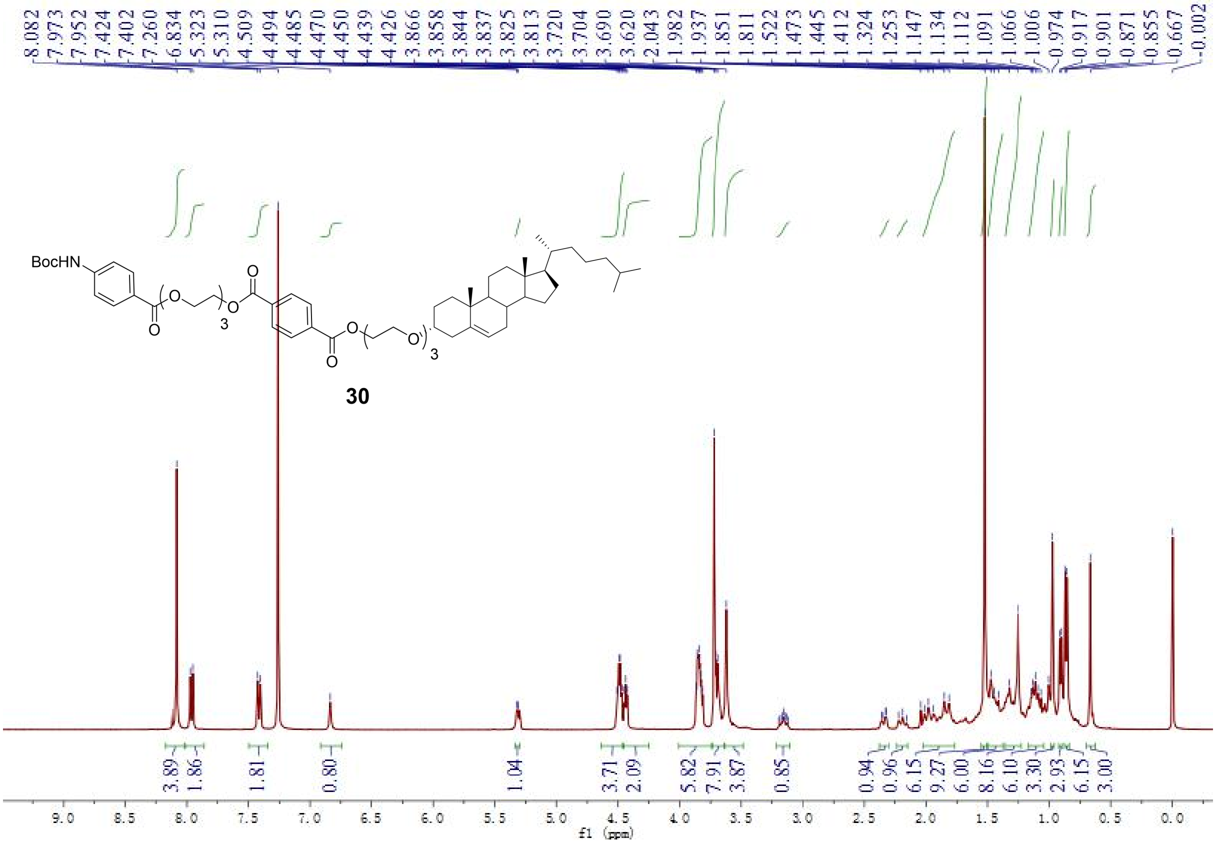

Figure S47. ^{1}H-NMR of compound **31**

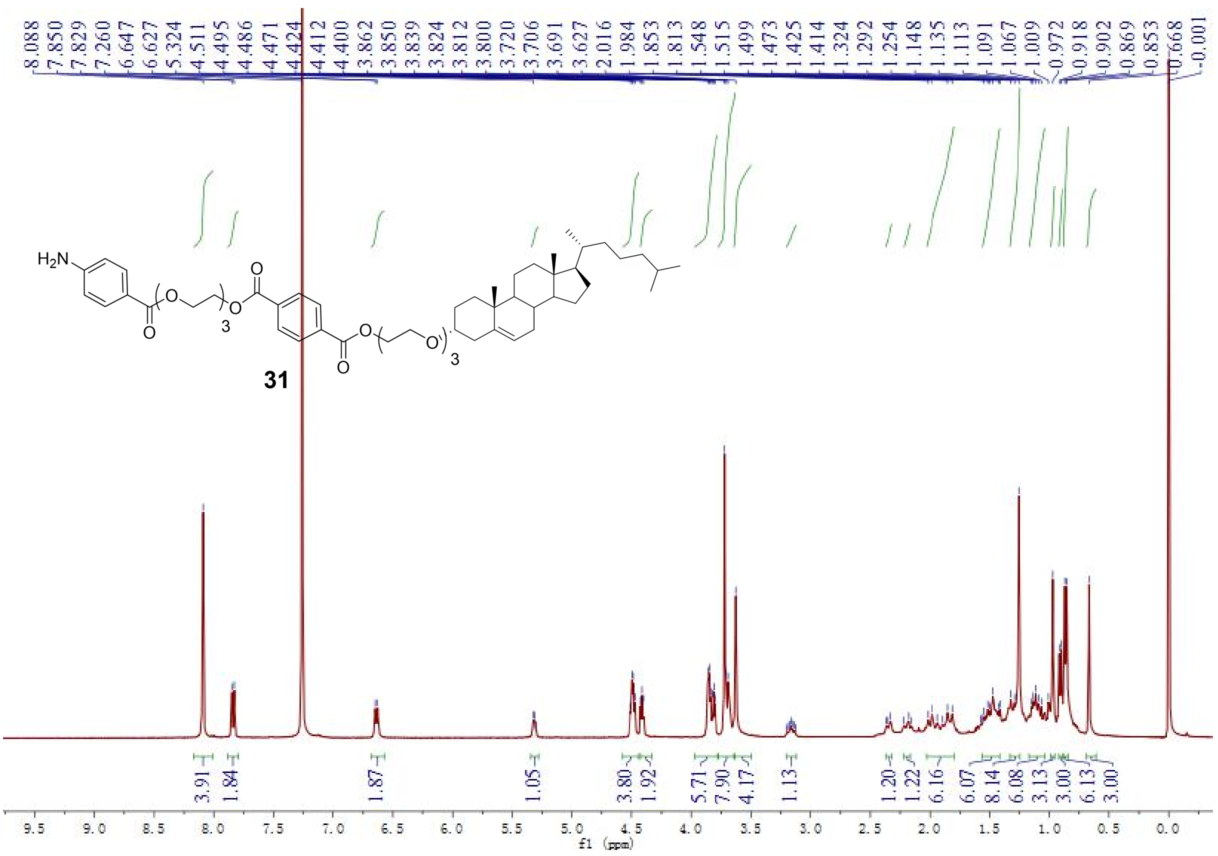

Figure S48. ^{1}H-NMR of ligand **Bio$_3$-PEG-PIm**

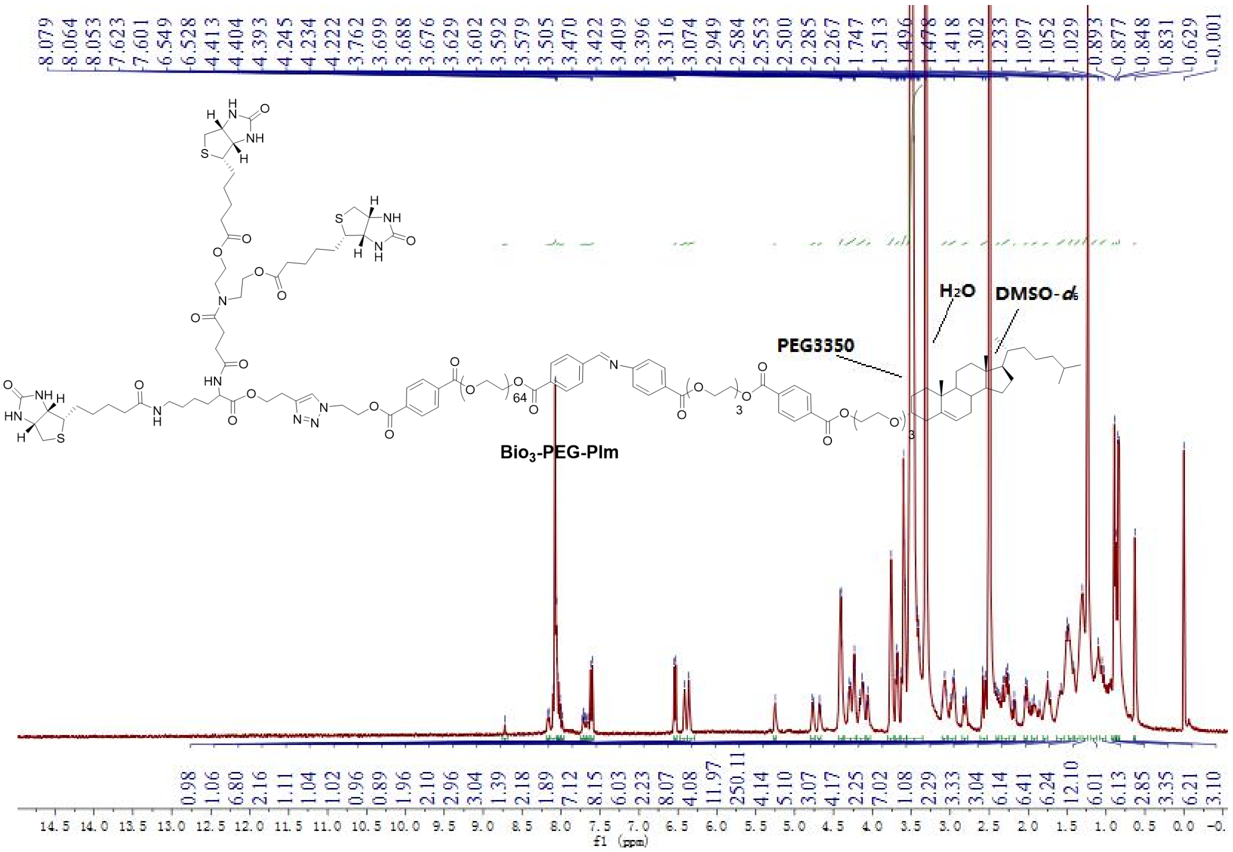

Figure S49. ^{1}H-NMR of compound **32**

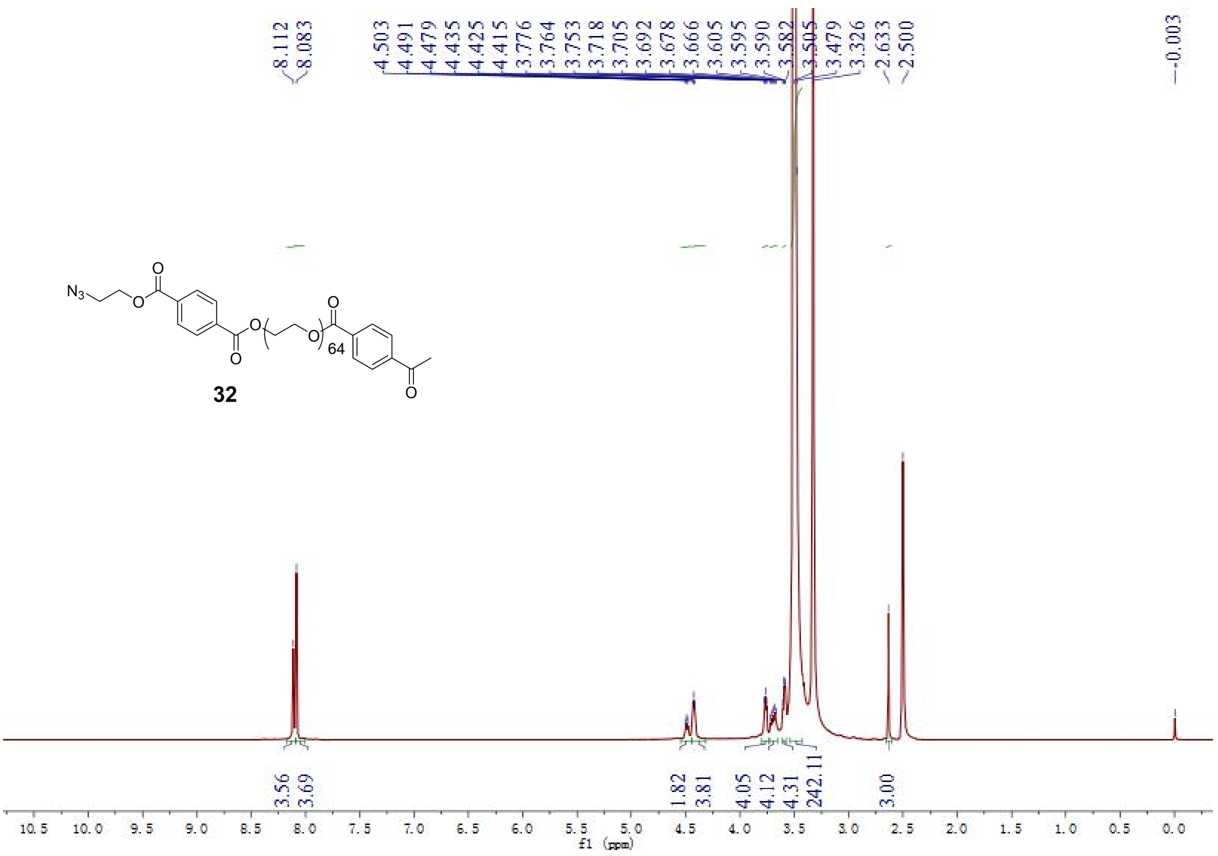

Figure S50. ^{1}H-NMR of compound **33**

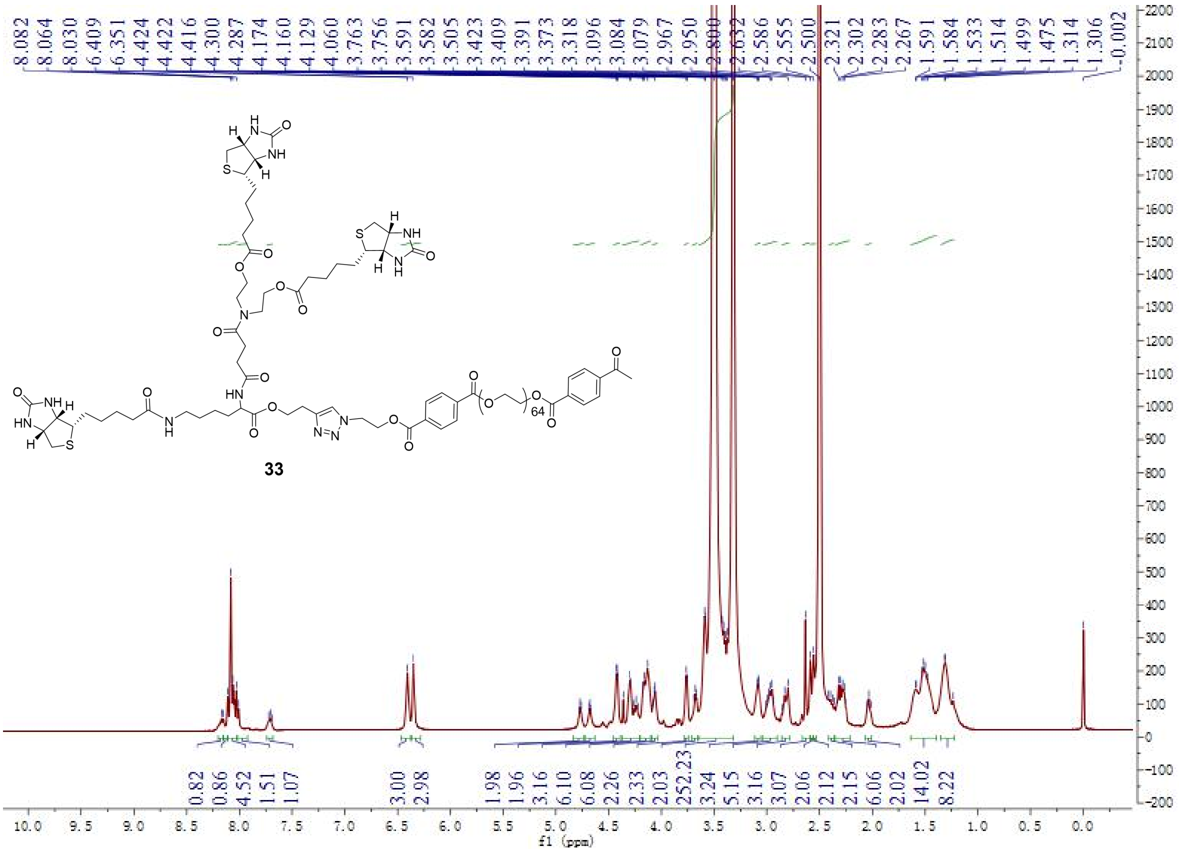

Figure S51. ^{1}H-NMR of ligand **Bio$_3$-PEG-Hz**

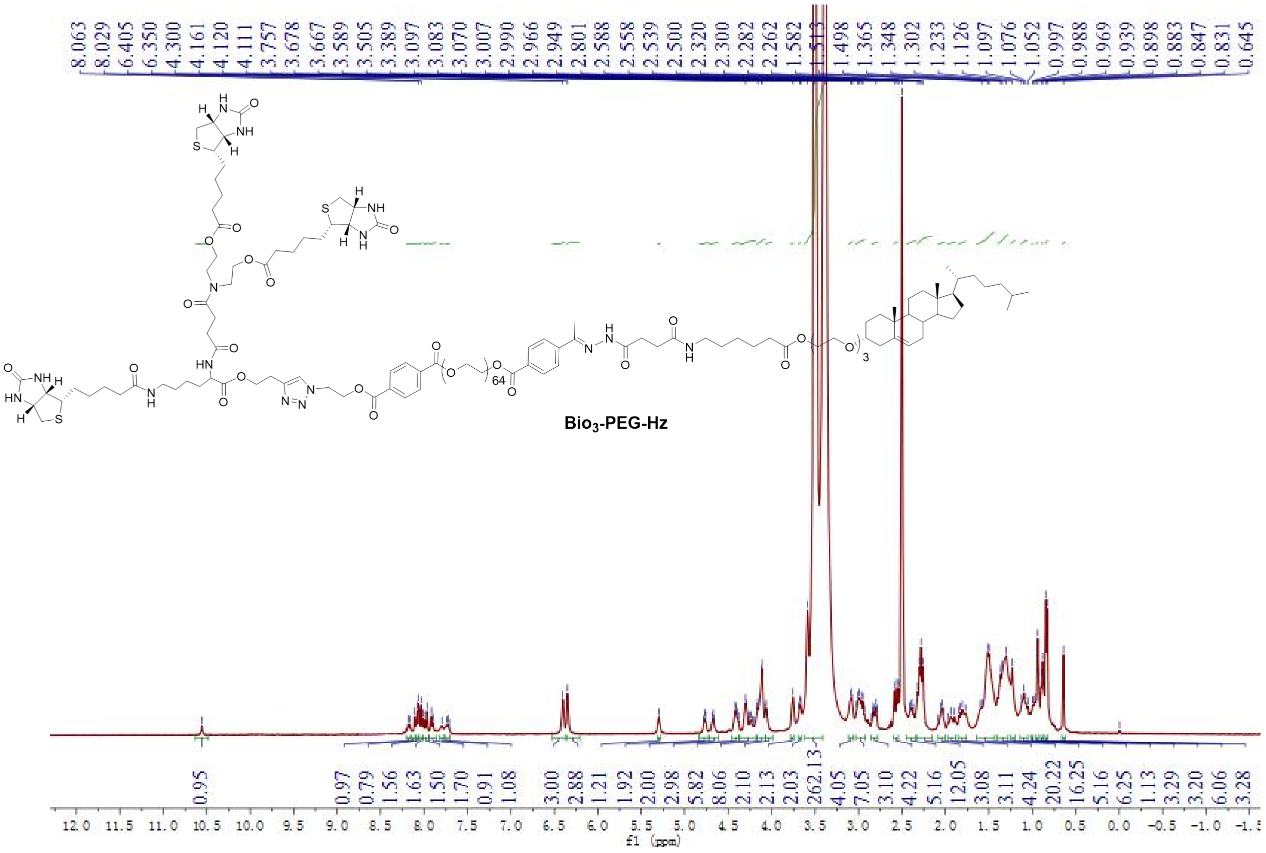

Figure S52. ^{1}H-NMR of ligand **Bio$_3$-PEG-BSa**

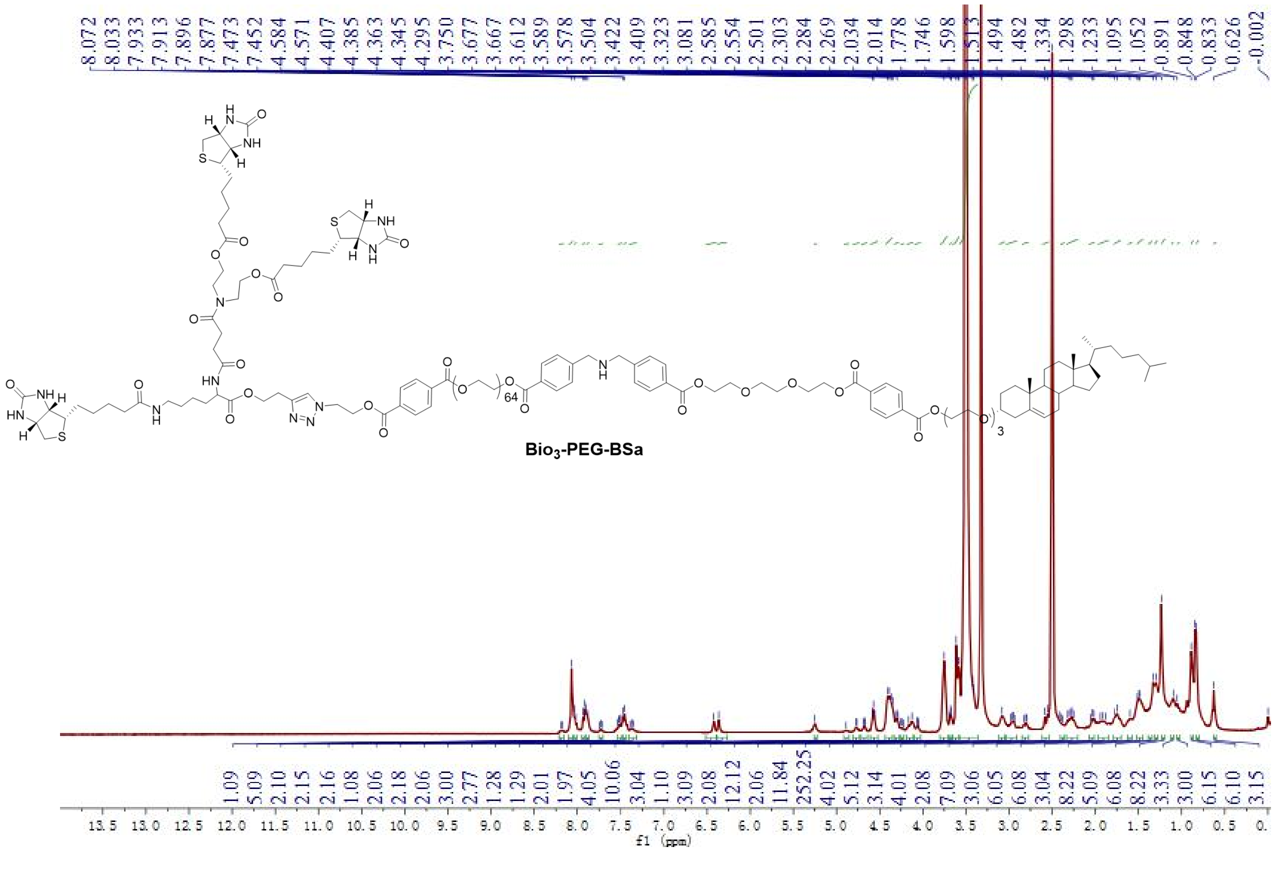

Supplement: Supplementary file 1 — Supplementary methods, figures and tables. [file thnov12p5299s1.pdf]
